# Reliable enteric methane prediction from the cattle (*Bos taurus*) rumen microbiome

**DOI:** 10.1038/s42003-026-10048-8

**Published:** 2026-04-13

**Authors:** Boris J. Sepulveda, Oscar González-Recio, Amanda J. Chamberlain, Ruidong Xiang, Benjamin G. Cocks, Jianghui Wang, Claire P. Prowse-Wilkins, Leah C. Marett, S. Richard O. Williams, Joe L. Jacobs, Aser García-Rodríguez, Jose A. Jiménez-Montero, Jennie E. Pryce

**Affiliations:** 1 Agriculture Victoria Research, AgriBio, Centre for AgriBioscience, Bundoora, VIC Australia; 2 School of Applied Systems Biology, La Trobe University, Bundoora, VIC Australia; 3 Departamento de Mejora Genética Animal, Instituto Nacional de Investigación y Tecnología Agraria y Alimentaria (CSIC), Madrid, Spain; 4 Department of Animal Science, University of California, Davis, CA USA; 5 Agriculture Victoria Research, Ellinbank, Bundoora, VIC Australia; 6 Department of Animal Production, NEIKER-BRTA, Granja Modelo de Arkaute, Vitoria-Gasteiz, Spain; 7 Spanish Holstein Association (CONAFE), CTRA, Madrid, Spain

**Keywords:** Computational models, Agricultural genetics, Agriculture, Metagenomics

## Abstract

The production of methane, a potent greenhouse gas, by ruminants during feed digestion is designated enteric methane emissions (EME) and is mainly produced by the rumen microbiome. Reliably recording EME in large populations is currently cost-prohibitive, hampering farming decisions aimed at reducing EME. Here, we perform comprehensive analyses on host genetics, KEGG orthology groups (KOs) from the rumen metagenome, and EME of more than 800 cows from Australia and Spain. We report that the rumen microbiome explains up to 34% of the EME variance, and when combined with the host genome, the variance explained is up to 59% with prediction accuracies of up to 0.40. The results support a recursive model, where both the host genome and rumen metagenome explain EME. The isometric log-ratio transformation of KOs may potentially better capture relationships between host genetics and the rumen microbiome than the centered log-ratio transformation, and BayesR yielded slightly higher microbe‑explained EME variance than best linear unbiased prediction. A forward simulation estimated to reach 90% of EME prediction accuracy with 6,000 animals with rumen microbiomes and host genomes, which could open opportunities for developing strategies to reduce EME. Our study contributes to the foundation for reducing EME, supporting global warming mitigation.

## Abstract (web summary)

Ruminal microbial genes and cattle (*Bos taurus*) genetics predict about 60% of enteric methane emission variation between animals, supporting strategies aimed at reducing these emissions.

## Introduction

Methane is a greenhouse gas with a warming potential 28 to 34 times greater than carbon dioxide over a century^1^ and has been the most significant contributor to global warming since the pre-industrial era^2^. Enteric fermentation of ruminants contributes to approximately 40% of global methane emissions^3^ and sums up 40% of total greenhouse gas emissions from livestock^4^. Beyond its environmental impact, methane also represents a loss of 2 to 12% of an animal’s net energy intake, directly tying it to feed efficiency^5,6^. Both genetic selection on EME and feed additives are feasible methods for mitigating enteric methane^6,7^. However, since there are 4 billion cattle worldwide, recording methane emissions at scale is a major bottleneck for its mitigation. Therefore, proxies of methane phenotypes are of great interest.

Enteric methane is generated during the acquisition of nutrients in ruminants, which include cattle and sheep. The plant material enters the rumen complex (a specialized forestomach in ruminants) and is subjected to microbial fermentation. Microbial fermentation primarily produces short-chain fatty acids, with methane as a by-product due to the existence of methanogens^8,9^. The host genome and the rumen microbiome are both reported to affect methane emissions^10^. Saborío-Montero et al.^11^ proposed a recursive model where both the host and rumen microbiome affect EME, with additional links between the host genetics via interactions between the host and the rumen microbiome. However, the extent to which the rumen microbiome predicts EME with the presence of host genetics remains to be determined.

A common approach for estimating the predictability of EME involves using linear mixed models that partition the variance in EME across the rumen microbiome, host genome and their interactions^10^. When using mixed models, an animal relationship matrix is constructed using the abundance of microbiome features together with other relationship matrices^10^. These groups of ruminal features are known as ‘ruminal microbiome cores’^12^ and are usually composed of taxonomic features^10^. However, few rumen microbial taxa are measurable in all animals^13^ and methods dealing with missing taxa remain debatable^6^. Additionally, the accuracy of the prediction of EME from the rumen microbiome has usually been evaluated as a one-time correlation between the predictor and the phenotype, which may be less reliable compared to the predictor evaluated using cross-validation approaches.

Here, we introduce a method using non-taxonomic ruminal features, avoiding imputation of missing data, and applying different statistical approaches to reveal that the rumen microbiome reliably predicts the phenotypic variance in EME. By partitioning variance in EME across the effects of host genetics, rumen microbiome and their interactions, we show that the EME mediated by the rumen microbiome remains independent from the host genome effect on EME. A forward simulation shows that EME can be predicted accurately using both host genome and rumen microbiome data measured on about six thousand animals.

## Results

**Fig. 1:**
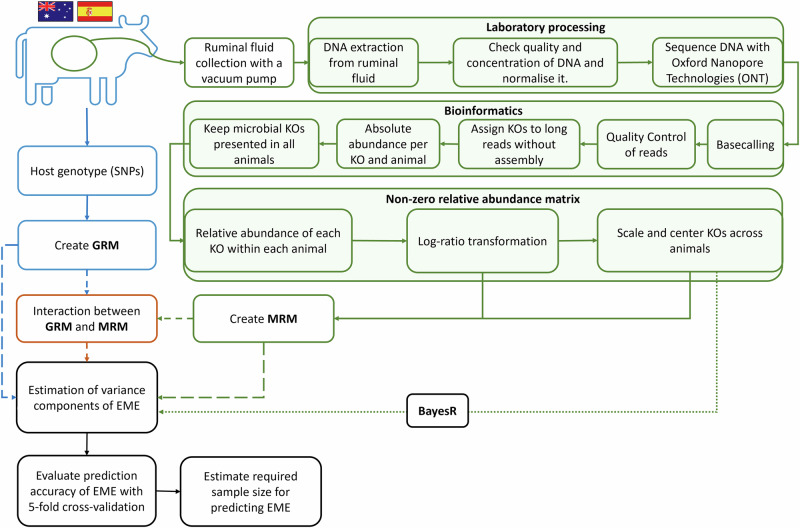
Study design of variance in enteric methane emissions (EME) using host genomics, ruminal metagenomics, and their interaction from 403 Australian and 426 Spanish dairy cattle. SNP Single nucleotide polymorphisms, KOs Kyoto Encyclopaedia of Genes and Genomes (KEGG) orthology groups. GRM Genomic relationship matrix, MRM Rumen microbiome relationship matrix. Two approaches were used to estimate the variance components: (1) Best Linear Unbiased Predictions (BLUP; dashed lines) and (2) BayesR (dotted lines).

We first estimated a rumen core microbiome of Kyoto Encyclopaedia of Genes and Genomes (KEGG) orthology groups (KOs)^14^ in 403 genotyped Australian and 426 genotyped Spanish cattle. In each dataset, we used a rumen microbiome core to build an animal microbiome relationship matrix (MRM), which was analysed together with the genomic relationship matrix (GRM) in linear mixed models to estimate their effect on EME. In this modelling, we evaluated the following scenarios: (1) GRM only, (2) MRM only, (3) GRM + MRM without interactions, and 4) GRM + MRM + the interaction between them. We also evaluated the KOs and the host SNPs as predictors of EME in BayesR3^15^. In addition, we conducted forward simulations where we estimated the sample size required for accurate prediction of EME. The steps taken are shown visually in Fig. 1.

### Description of the ruminal core

**Fig. 2:**
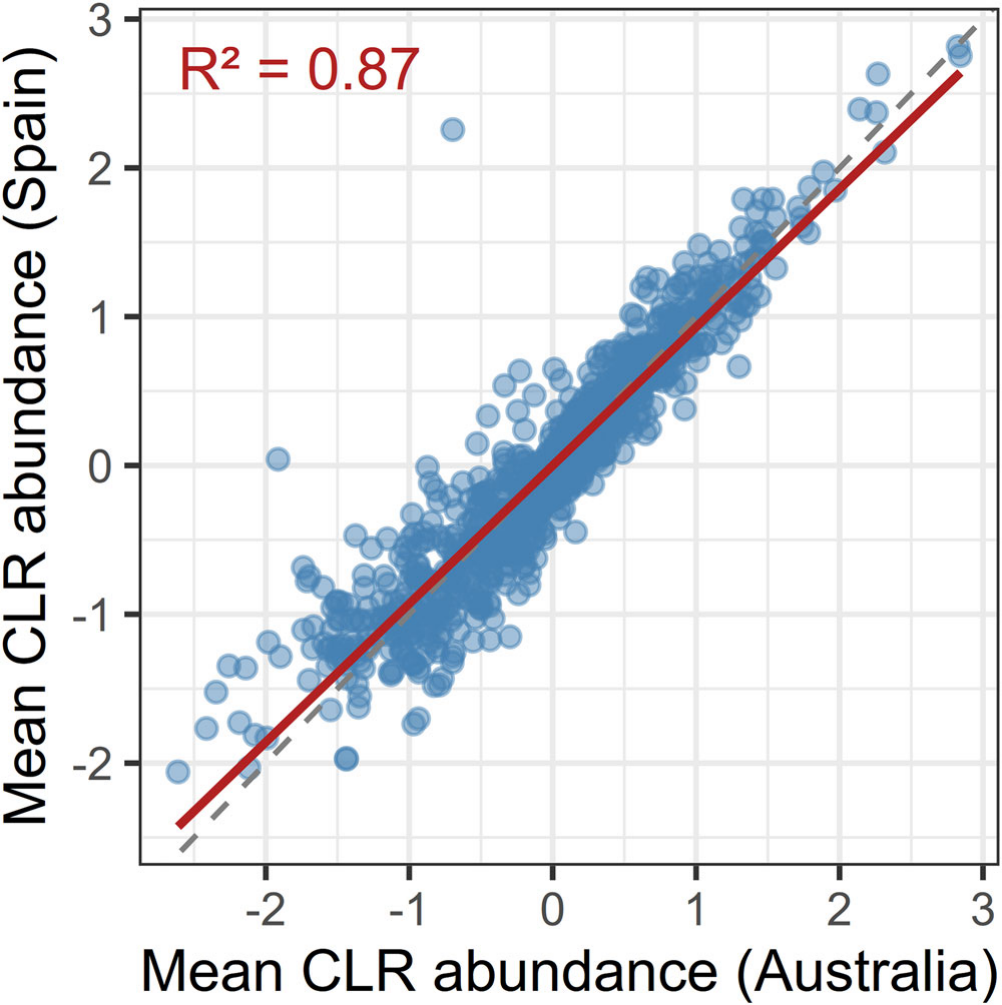
Regression between the mean-centered log-ratio-transformed relative abundances of a core of 1032 KEGG orthologs (KO) from the rumen metagenome of dairy cattle from Australia and Spain. R²: coefficient of determination. Solid line: the regression line. Dotted line: identity line.

A common core of 1032 KOs was identified in all animals of both Australian and Spanish populations, i.e., these KOs showed a prevalence of 100%. The centered log-ratio (CLR)-transformed abundance of 903 KOs, which represent 88% of the KOs analysed, differed between Australia and Spain (*P*-value < 0.05; Supplementary Data 1). According to the KEGG database, 10 of the core KOs are involved in methane metabolism, 386 in metabolism (other than methane), 71 in genetic information processes, 67 in environmental information processing, 52 in cellular processes, and 3 in organismal systems. Some KOs have been reported as involved in more than one of these classifications. The regression of the CLR-transformed KO abundances in Spain on those in Australia had a coefficient of determination (R²) of 0.87 (Fig. 2).

### Estimation of variance components and prediction accuracy

EME in Australia was approximately normally distributed (Shapiro-Wilk *P*-value = 0.11; Kolmogorov–Smirnov *P*-value = 0.65; Supplementary Fig. 1). EME in Spain showed deviations from normality with Shapiro–Wilk (*p* = 5.7 × 10⁻⁷), but the Kolmogorov–Smirnov test did not reject normality (*p* = 0.16). The within-farm coefficient of variation for EME was approximately 0.18 in Australia and ranged between approximately 0.20 and 0.40 in Spain (Supplementary Fig. 2). Daily enteric methane production in the Australian population measured as grams per day (MeP_Australia_; g/d) was significantly influenced by cohort (*P* < 0.001), dry matter intake (DMI; *P* < 0.001), and live weight (*P* < 0.05), with days in milk (DIM) showing a tendency toward significance (*P* < 0.1; Supplementary Table 1). Individual methane concentration in Spain measured as parts per million (MeC_Spain_; ppm) was significantly influenced by the stage of lactation (*P* < 0.05; Supplementary Table 1). All fixed effects influenced the rumen metagenome features (KOs), with farm showing a particularly strong effect in Spain: 89% of KOs had a *P*-value below 0.05 and 80% below 0.01 for the farm effect on their log-transformed relative abundance.

**Fig. 3:**
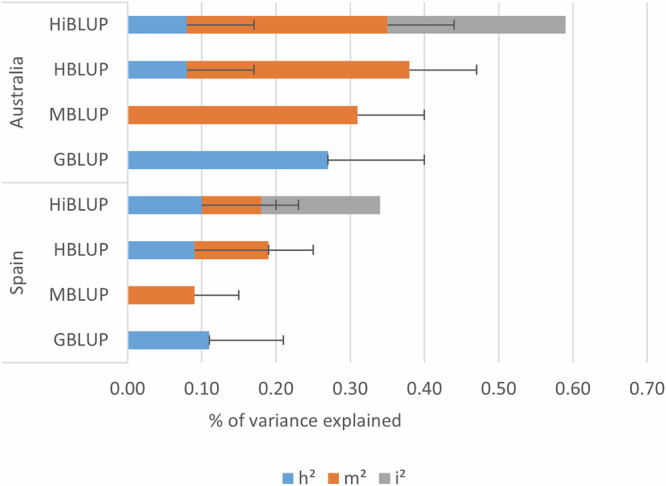
Quantifying the contribution of host genetics (blue), rumen microbiome (orange), and the interaction between host genetics and rumen microbiome (grey) to the variance of enteric methane emission using best linear unbiased predictions (BLUP). GBLUP is genomic BLUP, which includes the host genetics by fitting a relationship matrix (GRM). MBLUP is microbiomic BLUP, fitting a microbiome relationship matrix (MRM) constructed with isometric log-ratio transformed relative abundance of KEGG orthology groups scaled across animals, where only the microbiome was analysed. HBLUP is hologenomic BLUP, where both the GRM and MRM were fitted. HiBLUP is a hologenomic BLUP with interaction where GRM, MRM and their interaction were fitted. h²: heritability; m²: microbiability; i²: portion of the phenotypic variance explained by the interaction between the host additive genetic effect and the ruminal microbiota effect; ho²: holobiability. The standard error is represented by the black lines. The number of cattle in each population was higher than 400.

We first quantified the contribution of the genomic relationship matrix or GRM, microbiomic relationship matrix or MRM, and their interaction to the phenotypic variance in EME using best linear unbiased prediction (BLUP) models (Fig. 3). The heritability (h²), microbiability (m²), and holobiability (ho²) represent the proportions of phenotypic variance in EME explained by different biological components. Specifically, h² reflects the additive genetic contribution of the host, m² captures the contribution of the rumen microbiome, and ho² corresponds to the total variance explained by the host genome and microbiome together. To estimate these components, we used four BLUP models. GBLUP models the additive genetic effects of the host using the GRM. MBLUP models the effect of the rumen metagenome with the MRM. HBLUP combines both matrices to jointly estimate host genetic and microbial contributions to the phenotype. HiBLUP further extends HBLUP by including the interaction between the host genome and the microbiome. Additionally, MRMs were constructed using KOs transformed with either isometric log-ratio (ILR) or CLR, and the results obtained from these two types of matrices were compared to assess how different approaches for constructing microbiome relationship matrices influence the resulting estimates.

In Australia, EME heritability was estimated as 0.27 ± 0.13 (SE) in GBLUP and 0.08 ± 0.09 (SE) in HBLUP and HiBLUP. In Spain, heritability estimates were 0.11 ± 0.10 (SE) in GBLUP, 0.09 ± 0.10 (SE) in HBLUP, and 0.10 ± 0.10 (SE) in HiBLUP. Using ILR-based microbiome relationship matrices (MRMs), microbiability ranged from 0.27 to 0.31 in Australia and from 0.08 to 0.10 in Spain. The GRM×MRM interaction variance (i²) was 0.24 ± 0.13 (SE) in Australia, and 0.16 ± 0.13 (SE) in Spain, and holobiability was 0.59 ± 0.15 (SE) in Australia and 0.34 ± 0.16 (SE) in Spain. Relative to MRMs constructed with ILR, MRMs constructed with CLR yielded microbiability estimates 3 to 4% lower in Australia (Supplementary Table 2). In Australia, the heritability from HBLUP and HiBLUP was 5 to 6% lower with CLR than ILR. Of the 100 KOs with the largest effects on EME in the MBLUP analysis, 48 were shared between Australia and Spain (Supplementary Data 2). Besides the BLUP method, we also estimated the microbiability with a Bayesian (BayesR) model implemented in BayesR3^15^ to confirm the results obtained with BLUP. The microbiability obtained with BayesR3 was 0.34 with both CLR and ILR in Australia (95% Highest Posterior Density [HDP]: 0.11 to 0.58) and 0.16 with CLR and 0.17 with ILR in Spain (HDP: 0.00 to 0.46). Regarding the heritability of the core KOs, although most of them were not heritable (h^2^ close to zero), approximately 100 KOs showed heritability of at least 0.20 in both countries, with maximum estimates approaching 0.50 (Supplementary Fig. 3).

**Fig. 4:**
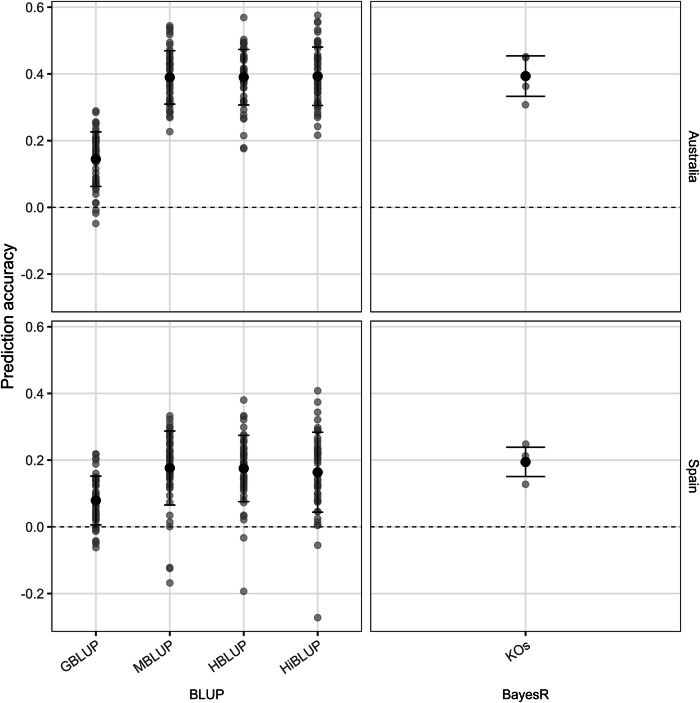
Cross-validation prediction accuracy on enteric methane emissions (EME) obtained with best linear unbiased predictions (BLUP) and BayesR in two populations of dairy cattle located in Australia and Spain. GBLUP: genomic BLUP, which includes the host genetics by fitting a relationship matrix (GRM). MBLUP is microbiomic BLUP, fitting a microbiome relationship matrix (MRM) constructed with isometric log-ratio transformed relative abundance of KEGG orthology (KO) groups scaled across animals, where only the microbiome was analysed. HBLUP is hologenomic BLUP, where both the GRM and MRM were fitted. HiBLUP is a hologenomic BLUP with interaction, where GRM, MRM, and their interaction were fitted. BayesR model fitting KOs as predictors. Grey dots: correlation between predicted and observed EME in the validation set. Black dots and bars: mean and standard deviations of these correlations. The number of validation sets (n) in BLUP models is 50 and in BayesR is 5.

An important question is whether host genetics and rumen microbiome can be used to predict EME. To answer this, we calculated the prediction accuracy with a 5-fold cross-validation correlation (10 repetitions in the BLUP models) using independent reference and validation datasets in each Australian and Spanish dataset separately. The prediction accuracy of GBLUP was 0.15 ± 0.11 (SE) in Australia and 0.07 ± 0.09 (SE) in Spain (Fig. 4**;** Supplementary Table 2). When using the MRMs constructed with ILR, the prediction accuracy of MBLUP, HBLUP and HiBLUP was between 0.39 ± 0.08 (SE) and 0.40 ± 0.09 (SE) in Australia and between 0.17 ± 0.12 (SE) and 0.18 ± 0.12 (SE) in Spain. The predictions obtained with CLR were 11% lower in Australia and between 6 and 20% lower in Spain. Similar prediction accuracies were obtained with BLUP and BayesR, being 0.39 ± 0.07 (SE) with ILR in Australia and 0.20 ± 0.04 with ILR in Spain (Fig. 4). The prediction accuracy was similar with CLR using BayesR: 0.40 ± 0.13 in Australia and 0.17 ± 0.12 with CLR in Spain. Additionally, the regression slope from adjusted EME on the estimated microbial values(b_EME, EMV_) was 0.77 ± 0.20 (SE) in Australia and 0.90 ± 0.18 (SE) in Spain using ILR, indicating possible overfitting. The b_EME,EMV_ was 0.84 ± 0.40 (SE) in Australia and 0.93 ± 0.75 (SE) in Spain using CLR, which also indicates a possible overfitting.

### KEGG enrichment analysis

Forty KEGG pathways were identified as significantly represented by the 1032 KOs used to estimate the EME variance (Benjamini–Hochberg adjusted *p* < 0.001) (Supplementary Fig. 4; Supplementary Data 3). These 40 pathways were involved in global and overview maps, glycan biosynthesis and metabolism, replication and repair, amino acid metabolism, nucleotide metabolism, energy metabolism, metabolism of cofactors and vitamins, carbohydrate metabolism, cell growth and death, drug resistance, folding, sorting and degradation, metabolism of terpenoids and polyketides and biofilm formation. Estimation of Reference Population Size.

**Fig. 5:**
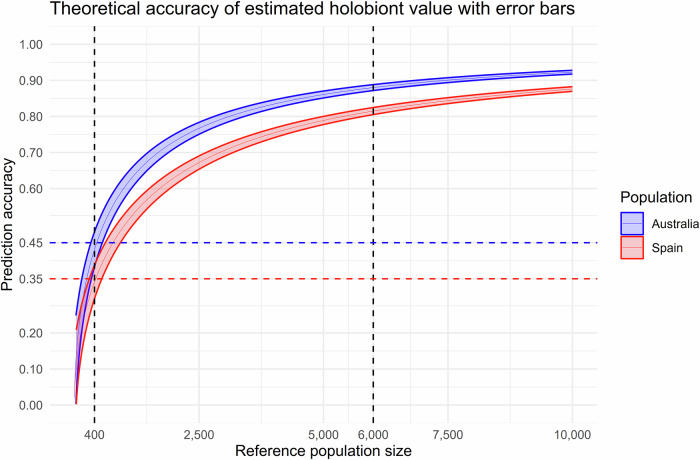
Estimated prediction accuracy of enteric methane emissions in relation to hypothetical reference population size of dairy cattle, incorporating host genomics and ruminal metagenomics (KEGG ortholog groups scaled across animals). The estimations are based on the variance components obtained in this study. The shaded areas indicate the 95% confidence intervals based on the standard error of the predictions. Dotted blue and red lines indicate the approximate prediction accuracy with 400 animals based on the holobiont estimations in Australia and Spain, respectively.

We used a forward simulation approach by extending the methods from Ross and Hayes^16^ to estimate the sample size needed to accurately predict EME after accounting for the host genetic effects (see methods). With this prediction method, an accuracy of between 0.80 and 0.90 would be reached with about 6000 animals, and an accuracy of about 0.90 would be achieved with 10,000 animals (Fig. 5). The accuracy predicted with 400 animals in Australia is approximately 0.45, which is between the range of the accuracy obtained with HiBLUP (0.41) and BayesR models (0.62). When applying the simulation to the Spanish dataset, the estimated accuracy is approximately 0.35, higher than the one obtained with HiBLUP (~0.18) and lower than the one obtained with BayesR (0.39).

## Discussion

Using data from two countries (Australia and Spain), we have evaluated the predictability of EME using host genetics and a core group of KOs from the rumen microbiome using BLUP and BayesR models and calculated the prediction accuracy with a 5-fold cross-validation. Although 903 KOs (88% of the core) showed statistically significant differences in mean CLR abundance between Australia and Spain (*P* < 0.05; Supplementary Data 1), the overall KO abundance profile was consistent between the two populations, as reflected by the strong correlation between mean CLR-transformed abundances of both countries (R² = 0.87; Fig. 2). Using ILR rather than CLR to construct the MRM led to higher microbiability estimates with up to 11% higher prediction accuracy (Supplementary Table 2), suggesting that ILR may provide a more informative representation of the compositional structure of the metagenome. When we fitted the ILR-based MRM jointly with the GRM and the GRM×MRM interaction, the prediction accuracy of HiBLUP was 11% superior in Australia and 20% superior in Spain compared with the CLR-based model. This pattern suggests that the ILR transformation may better reveal relationships between host genetics and the microbiome than CLR.

The EME variance explained by the rumen metagenome (microbiability) was up to 0.34 in Australia and 0.17 in Spain (results from BayesR). This result is comparable to previously reported values using various rumen microbiome sequence data and statistical methods. Zetouni et al.^17^ included an MRM constructed with Amplicon Sequence Variants (single DNA sequences) from the rumen microbiome in an HBLUP and reported a microbiability of zero. Difford et al. reported the microbiability of 0.13 for methane production, estimated with an HBLUP using an MRM matrix constructed with archaea and bacteria abundances estimated with 16S rRNA gene amplification, and sequencing^18^. In the same dataset from Difford et al.^18^ but using a Bayesian method to construct the MRM, Zhang et al.^19^ found a microbiability of 0.07. Ruminal genera and KOs were analysed together in a co-abundance network, grouped in clusters, and these clusters explained up to 57% of the EME variation in beef cattle^20^. In sheep, the reported microbiability of EME varies between 0.19 and 0.86, depending on how it was measured^21–23^. The wide range of results described here suggests the necessity of developing large reference populations and standard methodologies so that the EME variance explained by the rumen microbiome is comparable between studies. We used similar methodologies to compare two dairy cattle populations with respect to the effect of the ruminal microbiota on EME. We used the same laboratory technology (Oxford Nanopore Technologies long read metagenome sequencing), bioinformatic pipeline, and statistical approach in more than 800 dairy cows from Australia and Spain.

Apart from the higher EME microbiability in Australia compared to Spain previously discussed, other differences were found between populations. The EME predicted by the host genetics and the rumen microbiome (holobiability) was up to 0.59 in Australia and 0.34 in Spain (Fig. 3; Supplementary Table 2). The EME prediction accuracy of the models that used the rumen microbiome as a predictor was approximately 0.40 in Australia and between 0.17 and 0.20 in Spain (Fig. 4; Supplementary Table 2; BayesR results). Three reasons could explain these differences. First, EME from Spain were not adjusted for feed intake and body weight, which are highly associated with EME^24^. Second, possible variable number of sequencing reads, and discrepancies introduced by sample preparation and sequencing techniques^25^. Third, a higher environmental effect in the Spanish dataset could not have been captured by the model, where an average of 30 animals were analysed in each farm (426 animals in 14 farms), while all animals in Australia ( > 400) were from the same farm under the same productive system and similar feeding conditions. Supporting this third reason, the effect of the farm was significant in the abundance of most KOs (p-value < 0.05; Supplementary Table 1) and the coefficient of variation was higher within the Spanish farms compared to the Australian farm (Supplementary Fig. 2). Based on this analysis, further studies should include feed intake and animal weight into the EME prediction models, to use similar metagenome sequencing depth between batches, and to include as many animals as possible from each environment.

The EME heritability decreased in the Australian BLUP models when the microbiota was included in the prediction model, passing from 0.27 to ~0.13 when using CLR and 0.08 when using IRL (Fig. 3; Supplementary Table 2). These heritability reductions could suggest that part of the EME heritability was captured by the heritability of the microbiota features (Supplementary Fig. 3). Additionally, part of the EME variance remains independently explained by the host genome and the rumen metagenome in both approaches. These results are consistent with previous studies^10,18^ and support a recursive model, where both the host genetics and the microbiota exert influence on EME, and the host genetics also indirectly affect EME by modulating the microbiota^11^. This recursive model would explain why it is expected that breeding programs that simultaneously select for lower emissions and heritable rumen metagenome features genetically correlated with EME could achieve greater reductions in emissions compared to selecting for lower emissions alone^26,27^.

Although BayesR and BLUP produced similar mean prediction accuracies, BayesR exhibited slightly lower standard errors of these accuracies, indicating more stable performance across cross-validation folds (Fig. 4**;** Supplementary Table 2). BayesR also explained more of the EME variance attributable to the rumen microbiome, with microbiability estimates of 0.34 in Australia and 0.17 in Spain, compared with approximately 0.30 in Australia and 0.10 in Spain using BLUP. Unlike REML-based methods such as BLUP, BayesR does not assume normally distributed residuals or homoscedasticity, making it more flexible for modelling complex, potentially non-Gaussian relationships between the rumen microbiome and EME. This flexibility likely contributes to its improved performance, particularly given that there are KOs with large effects on EME^26,28,29^. More flexible models, such as those using machine learning, could possibly increase the EME microbiability and improve the EME prediction accuracy from the rumen microbiome. The same Australian dataset used here, but complemented with more metagenomic features, was used by Hsieh et al.^30^ to predict EME with machine learning and the cross-validated prediction accuracies ranged from 0.38 to 0.51, higher than those we obtained.

Variance components in the BLUP models were estimated using REML-based linear mixed models, which assume normally distributed and homoscedastic residuals. EME in Australia was approximately normally distributed (Shapiro–Wilk *P*-value = 0.11; Kolmogorov–Smirnov *P*-value = 0.65; Supplementary Fig. 1). EME in Spain showed deviations from normality with Shapiro–Wilk (*p* = 5.7 × 10⁻⁷), but the Kolmogorov–Smirnov test did not reject normality (*p* = 0.16). Although the confidence intervals for most variance components in Spain include zero (Supplementary Table 2), meaning that we cannot rule out the absence of these effects, the point estimates are positive. This suggests that there may be contributions from host genetics, the rumen metagenome, and their interactions to EME; however, these findings should be interpreted cautiously and warrant confirmation in larger datasets.

To complement REML, we also fitted BayesR, a Bayesian mixture method with more flexibility than REML, and the overall trend of the results from BayesR was similar to the results from REML, in terms of microbiability, prediction accuracy and a possible model overfitting. Future work could assess distribution-free approaches such as Rao’s MINQUE/MINQE^31^ for variance component estimation of EME. To address near-singularity in the relationship matrices, we applied ridge regularization by adding a small constant to the diagonal elements. Alternative methods, such as applying singular value decomposition (SVD)^32^ followed by Winsorizing the smallest singular value, could be tested. Using the same metagenomic dataset, the first two principal components of KO abundance within each population were correlated with EME^27^, suggesting that the dominant axes of variation in the KO matrix are biologically informative. This supports the idea that SVD-based dimensionality reduction can retain relevant biological signal, and such approaches may warrant further exploration of EME variance explained by the KOs. Additionally, future approaches that retain more microbial KOs and combine them with other taxonomic and functional metagenomic features may better explain EME variance. Even though we used data from 2 different countries that feed and manage cows in different ways, validation of our findings across larger datasets that span diverse environmental and management conditions is essential to assess the generalizability of our results.

The most significant enriched pathway, biosynthesis of amino acids (ko01230; Supplementary Fig. 4**;** Supplementary Data 3), could be associated with EME because amino-acid metabolism influences hydrogen and formate flows that regulate methanogenesis^33^. The second most significant enriched pathway was biosynthesis of cofactors (ko01240), which includes genes involved in the synthesis of cofactors for methanogenesis, such as Coenzymes M, F420, F430, methanopterin, methanofuran, and methanophenazine^34,35^. Other enriched pathways reveal less direct but biologically plausible links. The connection between peptidoglycan biosynthesis (ko00550) and EME could be explained by the fact that many methanogenic archaea possess pseudomurein, a structural analogue of peptidoglycan^36^, and archaeal phage lysins capable of hydrolyzing this polymer have been shown to inhibit methanogen growth and methane production^37^. Pyrimidine metabolism (ko00240) is involved in the valine, leucine, and isoleucine biosynthesis^14^, which are precursors for the formation of branched-chain volatile fatty acids (BCVFAs) in the rumen, which in turn are known for having an inhibitor effect on methanogens^38,39^. 2-Oxocarboxylic acid metabolism (ko01210) is involved in methane production via coenzymes M and B biosynthesis^14,40^. Martínez-Álvaro et al.^26^ also reported associations between EME and rumen metagenome KOs ko01210 and ko00240 in cattle. Biofilm formation pathways (ko02025, ko02026, ko05111) are closely linked to EME, as biofilms provide structured microbial consortia where methanogenesis regulates hydrogen pressure to sustain fermentation^41^.

Several enriched functions are closely tied to central carbon and amino acid metabolism (ko00010, ko00620, ko00020, ko01200, ko00500, ko00051, ko00052, ko00040, ko00290, ko00250, ko00260, ko00270, ko00300, ko00340, ko00400, ko00240, ko01230, ko01210, and ko00900; Supplementary Fig. 4**;** Supplementary Data 3). Increased activity in these pathways is expected to enhance the production of reducing equivalents such as hydrogen and formate, which are substrates for methanogens^42,43^. Shifts toward alternative electron sinks, such as succinate and propionate formation, can reduce methane production by competing for reducing equivalents^44^. Pathways involved in cell envelope structure and biosynthesis (ko00520, ko00540, ko00552, ko00550, ko00541, ko01250, and ko01502) may not directly affect methane production but could possibly reflect structural and community compositional shifts. Because these pathways are central to bacterial cell wall and envelope maintenance, they may indirectly influence microbial stability, which in turn can modulate microbial interactions (Leahy et al., 2013). Redox and cofactor-related pathways, such as ko00740, may influence the rumen microbial growth^45^. Pathways related to maintenance, replication, and regulation were also enriched (ko03030, ko03430, ko03440, ko01232, ko03060, ko04112), and although they do not contribute directly to methanogenesis, their activity likely reflects microbial growth dynamics, DNA repair capacity, and community turnover.

Methane production in the rumen is a consequence of complex microbial interactions that determine the fate of hydrogen and other reduced substrates generated during fermentation. Although archaeal methanogenesis is the terminal pathway, multiple upstream bacterial and archaeal processes are implicated in the availability of substrates and cofactors required for methane formation (Ungerfeld, 2020; Janssen and Kirs, 2008). The enrichment of several KEGG pathways suggests both direct and indirect functional connections between these pathways and EME. This suggests that EME are not solely determined by the abundance of methanogenesis genes, but also by the broader metabolic context of the rumen microbiome. From an applied perspective, this evidence supports targeting not only methanogenesis genes themselves, but also upstream microbial processes and community-level interactions as part of integrated methane mitigation strategies. Selective breeding, dietary interventions, or microbiome modulation strategies that reduce hydrogen availability or alter cofactor biosynthesis could yield methane reductions.

Currently, large-scale phenotyping of EME is very expensive and, therefore, requires careful experimental design for predicting/estimating methane. This will require estimating the sample size given the accuracy to be achieved. We provide assistance to such a task by estimating the reference sample size for using host genetics and rumen microbiome to predict methane and have made the code available to the public to guide future experiments. The simulated accuracy estimated for a reference population of 400 animals ranged between approximately 0.35 and 0.45 (Fig. 5). This range is consistent with the cross‑validation prediction accuracy obtained from the real dataset in Australia (approximately 0.40; Fig. 4) and is higher than that observed in Spain (approximately 0.20). Based on the simulations and the results from Australia, the prediction accuracy of EME could reach approximately 0.80 to 0.90 with a reference population of around 6000 animals with both ruminal metagenome and host genotype data (Fig. 5). As previously discussed, higher prediction accuracies are expected for animals with similar genetics and under similar environmental conditions. These estimates are consistent with Rothschild et al.^46^, who, using more than 30,000 metagenome samples from humans, found a plateau in the prediction accuracy of the body mass index and haemoglobin A1C with approximately 4000 metagenome samples. The highly accurate predictions of EME derived from ruminal metagenomic data can lead to more informed farming decisions. For instance, removing animals with high-emission rumen profiles from the herd can reduce overall farm emissions or breeding animals with a natural predisposition to inherit a rumen microbiome profile associated with lower EME^26,27^. Another example is providing feed additives designed to reduce emissions only on higher-emitting animals rather than the entire herd, which could make the use of these additives more cost-effective.

In conclusion, our results suggest that rumen metagenome functional features accurately predict up to about 34% of the variance of enteric methane emissions. We found that the host genome and rumen metagenome are complementary in explaining up to 59% of enteric methane emissions variance and could be used together in strategies aimed at reducing these emissions. We provide guidance on the methods and the sample size for building a reference population to make accurate EME predictions. Our work provides fundamental knowledge, data and code to support large-scale prediction and mitigation of methane emissions in animal agriculture.

## Methods

Data were collated from experiments completed in Australia and Spain, where samples of ruminal fluid, estimates of EME, and host genotypes were available for 834 Holstein dairy cows. These data sets were used as input in a common workflow to estimate the percentage of the EME variance explained by the ruminal microbiota, the host genetics, and the interaction between these two (Fig. 1).

### Ethical statement

The experiments in Australia included in this study were approved and undertaken in accordance with the Australian Code of Practice for the Care and Use of Animals for Scientific Purposes (NHMRC, 2013). Approval to proceed was granted by the Agricultural Research and Extension Animal Ethics Committee of the Department of Energy, Environment and Climate Action (application number 2013-14 was approved on August 22nd, 2013, and application number 2016-12 was approved on August 22nd, 2016). The experiments in Spain included in this study were conducted in accordance with Spanish Royal Decree 53/2013 for the protection of animals used for experimental and other scientific purposes and were approved by the Basque Institute for Agricultural Research and Development Ethics Committee (Neiker-OEBA-2017-004) on March 28, 2017. We have complied with all relevant ethical regulations for animal use.

### Animals, environment, and host genotypes

The Australian population included 403 Holstein lactating adult female cattle (cows) located at the Ellinbank SmartFarm (Ellinbank, Victoria, Australia). These cows were measured for intake and EME in 11 cohorts between 2013 and 2017. At the beginning of the study, they averaged 110 ± 19.4 (mean ± SD) days in milk, 2.5 ± 1.25 (SD) lactations, and weighed 539 ± 69.8 (SD) kg. Over a 32-day period in an experimental facility, they had continuous access to feed, water, and a bare paddock (loafing area) for rest. The cows were outside except for twice-daily milking. Cows were fed with the diet described by Moate et al.^47^ and feed intake was measured using feed bins equipped with load cells and electronic monitoring linked to individual cow identification (Gallagher Animal Management Systems, Hamilton, New Zealand). Each cow’s daily dry matter intake was recorded over the 32 days. MeP_Australia_ was obtained with the sulphur hexafluoride (SF_6_) tracer method described by Deighton et al.^48^. Further details of the environment of the Australian dairy cattle population are provided by Moate et al.^47^.

The Spanish population included 426 Holstein cows, either in their first or second lactation, from 14 commercial farms across four Northern Spanish regions (Cantabria, País Vasco, Navarra, and Gerona). Following the methodology of Rey et al.^49^, EME were measured using a non-dispersive infrared methane detector (The Guardian® NG) from Edinburgh Sensors (Livingston, Scotland, UK), also termed “sniffer”, installed in the feed bin of an automatic milking system. MeC_Spain_ was recorded for each cow during milking over a period of two to three weeks. The recorded eructation peaks were averaged to obtain a single record per cow. The Spanish population was under commercial milk recording schemes consistent with ICAR-accredited recording protocols.

The cattle genotypic data were generated in previous studies and reused in this work. The animals located in Australia were genotyped with SNP arrays including custom genotyping-by-sequencing (GBS) and selected SNP (Illumina XT) panels (approximately 8800 SNPs of which at least 6900 overlapped with the BovineSNP50 BeadChip, Illumina, San Diego, California, USA) and then imputed to the Bovine 50 K SNP chip panel using the software FImpute^50^ as described by Haile-Mariam, et al.^51^. The animals located in Spain were genotyped with the EURO12K SNP chip (Illumina, San Diego, California, USA) and then imputed to 54,609 SNPs using BEAGLE software^52^ as described in Jiménez-Montero, et al.^53^ using the Spanish reference population provided by the Spanish Holstein Association (CONAFE, Madrid, Spain) containing more than 200,000 genotypes. A panel with 39,058 (40 K) SNPs shared by both populations and with a minor allele frequency greater than 0.05 was selected for analyses. The sample size in this study represents the maximum number of animals that could be included within the financial and animal ethical constraints of the project. Details of the host genotyping procedures, SNP‑calling pipelines, and imputation methods for the Australian and Spanish datasets are described in Haile-Mariam et al.^51^ and Jiménez-Montero et al.^53^, respectively.

### Ruminal sample collection

Ruminal fluid samples from Australian cows were collected via an oesophageal probe placed into the rumen via the mouth. An oro-ruminal sampling probe, similar to the one described by Geishauser^54^, and a vacuum pump were used to collect samples^55^. The oesophageal probe was a smooth-polished stainless-steel device comprising two parts: (1) a 170 mm long bolus of 42 mm diameter with a 2 mm screen and (2) a 4-metre-long flexible tube of 20 mm diameter. The collected samples were allowed to drain freely through a cheesecloth layer, separating rumen solids from the ruminal fluid, and then frozen at −80 °C. In Spain, a custom-built mechanical device was used to elevate the cow’s snout and approximately 100 ml of ruminal content from each cow was extracted as described by Saborío-Montero et al.^10^ by inserting a tube connected to a mechanical pump (Vacubrand ME 2SI, Wertheim, Germany) through the oesophagus. The obtained samples were then secured in sterilized containers. The hose and equipment were washed after each use. The samples were filtered using four layers of sterile cheesecloth to separate the solid elements, and the liquid portion was immediately frozen using liquid nitrogen vapours. These frozen samples were then conveyed to the lab in containers filled with liquid nitrogen and kept at -80 °C until analysis. The number of animals with rumen samples was 410 in Australia and 432 in Spain.

### Laboratory processing of ruminal samples

In Australia, microbial genomic DNA was extracted from 300 µl of ruminal fluid using a ZymoBIOMICS DNA miniprep kit (Zymo Research, Irvine, California, USA) according to the manufacturer’s instructions, with beadbeating performed with a multi-speed vortex (Biofen MSV-3500) at 3500 rpm for 40 minutes. DNA quality and concentration were determined using a Nanodrop and Qubit and normalised to the same concentration. In Spain, the samples were thawed and homogenised, and the DNA was extracted from 250 µl of ruminal fluid using the DNeasy Power Soil Kit (QIAGEN, Valencia, California, USA), and DNA concentration and purity were measured using a fluorometer and spectrophotometer.

Long-read sequencing with Oxford Nanopore Technologies (ONT) was used for metagenome sequencing in Australian and Spanish populations. Australian DNA samples underwent end repair with NEBNext end repair kit (New England Biolabs, Notting Hill, Victoria, Australia), and then libraries were prepared using the Native Barcoding expansion kit (SQK-NBD112.96, Oxford Nanopore Technologies, Oxford, United Kingdom) according to the manufacturer’s instructions. Libraries were then sequenced using R9.4.1 flow cells on a PromethION24 sequencer (Oxford Nanopore Technologies, Oxford, United Kingdom) according to the manufacturer’s instructions. In Spain, one microgram of DNA per sample was used to prepare libraries using the ligation sequencing kit (SQK-LSK109, Oxford Nanopore Technologies, Oxford, United Kingdom) according to the manufacturer’s instructions. Twelve samples were multiplexed in each run, and the barcoded samples were pooled for adapter ligation and sequencing using an R9.4.1 flow cell on a MinION sequencer (Oxford Nanopore Technologies, Oxford, United Kingdom). Further information regarding sampling, DNA extraction and sequencing of Spanish samples is provided by Saborío-Montero et al.^11^ and Saborío-Montero et al.^56^.

### Bioinformatics

Basecalling for both the Australian and Spanish populations was conducted using the software Guppy (Oxford Nanopore Technologies, Oxford, United Kingdom) with high accuracy mode (HAC) using versions 5.0.16 and 4.2.2, respectively. The reads were quality-controlled, ensuring a Quality Score greater than seven and a sequence length greater than 150 base pairs. After applying this quality control, the tool NanoPlot^57^ was used with the options *fastq*, *N50* and *loglength* to characterise the reads. The long reads were aligned to the KEGG database^14^ for KO identification using the script SQM_longreads.pl of the SqueezeMeta pipeline (version 1.4)^58^ using the *euk* option to yield more eukaryotic annotations. This script produces taxonomic and functional assignments directly on the raw reads, not using an assembly and assuming that more than one open reading frame (ORF) can be found in the read^58^. SqueezeMeta performs Blastx with Diamond^59^ against taxonomic and functional databases, and then identifies ORFs by collapsing the hits in the same region of the read^58^. In Australia, the mean read length was 5.0 × 103 ± 9.6 × 102 (SD) bases and the mean read quality (Q-score) 1.5 × 101 ± 4.6 × 10; the number of reads range between 1.5 × 102 and 1.0 × 106 (2.3 × 105 ± 1.2 × 105; SD), the N50 read length between 3.2 × 103 and 1.3 × 104 (7.1 × 103 ± 1.6 × 103; SD), and the total bases between 5.6 ×105 and 5.2 × 109 (1.1 × 109 ± 5.5 × 108; SD). In Spain, 5.0 × 107 reads were processed with SqueezeMeta and 2.6 × 107 of them (51.79%) were mapped to the KEGG database^29^.

### Selection of rumen metagenomic features

Ideally, a microbiome relationship matrix (MRM) should be created with metagenomic features that are in all animals because this matrix is created after logarithmic transformations, which requires non-zero (non-null) values^60^. The null values can be imputed to non-zeros^10,21,61^, but how best to deal with these zeros remains an open research topic^6^. Additionally, from a breeding viewpoint, excluding features with null values is ideal, as it allows for comparing all selection candidates with the same microbial features. The KOs meet these criteria. There are more KOs than taxonomic features with non-zero abundances, which facilitates the construction of an MRM. More than 3,000 KOs from the bovine rumen and pig gut had shown a prevalence of 100%, notably higher than the less than 100 Phylum, Genus, and Species present in all animals^13,62^. This larger number of KOs could make the models' fitting MRM easier to converge. Additionally, the KOs could be more consistently associated with EME than taxonomic features. Some KO groups have been reported as associated with EME^26,28,29^. Different cattle populations may have the same ruminal microorganism but with different microbial genes, which could cause this microorganism to have different associations with EME^16^. In contrast, the same microbial gene, or gene function (KO), could be consistently associated with EME across cattle populations.

We excluded the KOs that included genes from *Bos taurus* (cow). To identify these KOs, we used an in-house bash script that fetched the KEGG ID of *Bos taurus* (“BTA:”) from the KEGG database and removed these KOs from the dataset. After this filter, 1,032 KOs remained, all of which were present in all animals of both populations. We classified these KOs based on their associated microbial biological processes, as delineated in the KEGG database consulted in May 2024 (Supplementary Data 1). These classifications encompassed a range of processes: (1) Methane metabolism (KEGG pathway ko00680); (2) Metabolism under the KEGG category 09100 exclusive of methane-related activities (termed as ‘Metabolism (other than methane)’); (3) Genetic information processing (KEGG category 09120); (4) Environmental information processing (09130); (5) Cellular processes (09140); and (6) Organismal systems (09150). This categorization was facilitated by an in-house bash script utilising the curl command to fetch the KEGG identifiers for the mentioned processes from the URL of the KEGG database. The difference between the RA of the KOs was calculated as the absolute value of the difference between the RA in each population.

### Data transformation

The 1032 KOs previously mentioned were used to construct two absolute abundance matrices, one per population, with dimensions animals times KOs and populated with the number of reads assigned to each KO in each animal. Then, a relative abundance (RA) matrix was created from each population as the proportion of each KO’s absolute abundance compared to the total abundances in the same animal. The resulting matrix (${{{\bf{B}}}}$) was a non-zero relative abundance matrix with animals in the rows and KOs in the columns. As matrix ${{{\bf{B}}}}$ was compositional^60^, it was normalised to allow the comparison between animals. The normalization was performed with two methods, the centered log-ratio (CLR) transformation and the isometric log-ratio transformation (ILR)^63^. The CLR transformation was implemented with the unweighted option of the *CLR* function of the easyCODA R package^64^ as:

$${{{\bf{CLR}}}}[{B}_{{ij}}]=\log \left(\frac{{B}_{{ij}}}{{{{{\rm{g}}}}}_{({{{{\bf{B}}}}}_{i})}}\right)\,$$

where ${{{\bf{CLR}}}}$ represents the CLR transformed matrix, ${B}_{{ij}}$ is the entry for animal $i$ and KO $j$ in the matrix ${{{\bf{B}}}}$, and ${{{{\rm{g}}}}}_{({{{{\bf{B}}}}}_{i})}$ is the geometric mean of the vector of KOs within the animal $i$ defined as follows, and ${{{\rm{p}}}}$ is the number of KOs:

$${{{{\rm{g}}}}}_{({{{{\bf{B}}}}}_{i})}={\left({\prod }_{{{{\rm{j}}}}=1}^{{{{\rm{p}}}}}{B}_{{ij}}\right)}^{\frac{1}{{{{\rm{p}}}}}}$$

The **ILR** transformation was implemented with the function *ilr* of the compositions R package (v 2.0-8)^65^ as follows:

$${{{\bf{ILR}}}}\left[{B}_{{ij}}\right]={{{{\bf{V}}}}}^{t}{{{\bf{CLR}}}}[{B}_{{ij}}]$$

where ${{{{\bf{V}}}}}^{t}$ is the transpose of the orthonormal basis matrix ${{{\bf{V}}}}$, which projects the CLR-transformed data onto the ILR coordinates.

### Statistics and Reproducibility

#### Best linear unbiased predictions

A genomic relationship matrix (GRM) was created with genotypes of the SNP markers shared by both populations, utilising the *Gmatrix* function from the R package *AGHmatrix*^66^ following the approach of Yang, et al.^67^. Additionally, we constructed two MRM with the log-ratio transformed relative abundance of KOs (one with CLR and one with ILR) to estimate the proportion of the EME variance explained by the rumen microbiota, as follows:

$${{{\bf{M}}}}\,=\left(\frac{1}{{{{\rm{p}}}}}\right){{{{\bf{XX}}}}}^{{{{\rm{T}}}}}$$

Where ${{{\bf{X}}}}$ was constructed following previous studies in terms of scaling and centring the ruminal metagenome features (KOs in our case) across animals ^16,22,68^ using the function *scale* of the R programming language^69^. A small constant value (1 × 10^−8^) was added to each element of the main diagonal of the MRM matrices to circumvent singularity issues. This constant value was about 1,000 times smaller than the lowest KO’s average relative abundance. Then, GRM and MRM were inverted with the function *solve* of R^69^.

The variance components with the BLUP models were tested following the methodology of Saborío-Montero et al.^10^. EME of each country was included as the dependent variable (${{{\bf{y}}}}$) in univariate BLUP to estimate the proportion of the variance explained by the host additive genetic effect, the ruminal metagenomic effect, and the interaction between these two effects (Table 1).

**Table 1:** Prediction models to estimate the proportion of enteric methane emissions (${{{\rm{y}}}}$) explained by the fixed effects (${{{\rm{\beta }}}}$) additive genetic effect (${{{\rm{g}}}}$), the ruminal metagenomic effect (${{{\rm{m}}}}$), and their interaction (${{{\rm{g}}}}\times {{{\rm{m}}}}$) in dairy cattle from Australia and Spain

Model	Form
Genomic BLUP (GBLUP)	${{{\bf{y}}}}={{{{\bf{1}}}}}^{{\prime} }{{{\rm{\mu }}}}{{{\boldsymbol{+}}}}{{{\bf{X}}}}{{{\boldsymbol{\beta }}}}{{{\boldsymbol{+}}}}{{{\bf{Uh}}}}+\,{{{\bf{Zg}}}}+{{{\bf{e}}}}$
Microbiomic BLUP (MBLUP)	${{{\bf{y}}}}={{{{\bf{1}}}}}^{{\prime} }{{{\boldsymbol{\mu }}}}{{{\boldsymbol{+}}}}{{{\bf{X}}}}{{{\boldsymbol{\beta }}}}{{{\boldsymbol{+}}}}{{{\bf{Uh}}}}{{{\boldsymbol{+}}}}{{{\bf{Zg}}}}{{{\boldsymbol{+}}}}{{{\bf{Wm}}}}{{{\boldsymbol{+}}}}{{{\bf{e}}}}$
Hologenomic BLUP (HBLUP)	${{{\bf{y}}}}={{{{\bf{1}}}}}^{{\prime} }{{{\boldsymbol{\mu }}}}{{{\boldsymbol{+}}}}{{{\bf{X}}}}{{{\boldsymbol{\beta }}}}{{{\boldsymbol{+}}}}{{{\bf{Uh}}}}{{{\boldsymbol{+}}}}{{{\bf{Zg}}}}{{{\boldsymbol{+}}}}{{{\bf{Wm}}}}{{{\boldsymbol{+}}}}{{{\bf{e}}}}$
Hologenomic BLUP with interaction (HiBLUP)	${{{\bf{y}}}}={{{{\bf{1}}}}}^{{\prime} }{{{\boldsymbol{\mu }}}}{{{\boldsymbol{+}}}}{{{\bf{X}}}}{{{\boldsymbol{\beta }}}}{{{\boldsymbol{+}}}}{{{\bf{Uh}}}}{{{\boldsymbol{+}}}}{{{\bf{Zg}}}}{{{\boldsymbol{+}}}}{{{\bf{Wm}}}}{{{\boldsymbol{+}}}}{{{\bf{T}}}}\left({{{\bf{g}}}}\times {{{\bf{m}}}}\right){{{\boldsymbol{+}}}}{{{\bf{e}}}}$

${{{\rm{h}}}}$ is a random effect of robots used to measure emissions nested within farms (only used in Spain). ${{{\rm{X}}}}$, ${{{\rm{U}}}}$, ${{{\rm{Z}}}}$, ${{{\rm{W}}}}$, and ${{{\rm{T}}}}$ are incidence matrices; and $e$ is the residual.

In these models, ${{{\rm{\mu }}}}$ is the population mean; ${{{\bf{1}}}}$ is a vector of ones with the same length of ${{{\bf{y}}}}$; ${{{\boldsymbol{\beta }}}}$ is a vector of fixed effects; ${{{\bf{g}}}}$ is a vector of random additive genetic effects; ${{{\bf{m}}}}$ is a vector of random microbiota effects; ${{{\bf{g}}}}\times {{{\bf{m}}}}$ is the interaction between the additive genetic effect and the ruminal microbiota. ${{{\bf{X}}}}$, ${{{\bf{Z}}}}$, ${{{\bf{W}}}}$, and ${{{\bf{T}}}}$ are incidence matrices. The distribution of ${{{\bf{u}}}}$ is assumed ${{{\rm{N}}}}(0,{{{\rm{GRM}}}}{{{{\rm{\sigma }}}}}_{{{{\rm{g}}}}}^{2})$. The distribution of ${{{\bf{m}}}}$ is assumed ${{{\rm{N}}}}(0,{{{\rm{MRM}}}}{{{{\rm{\sigma }}}}}_{{{{\rm{m}}}}}^{2})$. The distribution of ${{{\bf{g}}}}\times {{{\bf{m}}}}$ is assumed ${{{\rm{N}}}}(0,{{{\rm{GRM\#MRM}}}}\,{{{{\rm{\sigma }}}}}_{{{{\rm{u}}}}\times {{{\rm{m}}}}}^{2})$ where # stands for the Hadamard product between GRM and MRM. Finally, ${{{\bf{e}}}}$ is a vector of random residuals distributed ${{{\rm{N}}}}(0,{{{{\rm{\sigma }}}}}_{{{{\rm{e}}}}}^{2})$. Following the methodology described by Saborío-Montero et al.^10^ four models were tested. These models were genomic BLUP (GBLUP) fitting GRM as a random effect; microbiomic BLUP (MBLUP) including MRM as a random effect; hologenomic BLUP (HBLUP) fitting both GRM and MRM as random effects; and hologenomic BLUP with interaction (HiBLUP), which included GRM, MRM, and their interaction as random effects.

MeP_Australia_ was used as EME in Australia, and the fixed effects were cohort (11 levels), dry matter intake, days in milk, energy corrected milk obtained with the methodology of Visscher et al.^70^, and daily body weight change during the experiment. The effect of the year in the Australian dataset was not significant, probably because the effect of the cohort captured it, as each cohort was confined to a single year. MeC_Spain_ was used as EME in Spain, the models included the robots used to measure emissions nested within farms (24 levels) as a random effect (represented as ${{{\bf{h}}}}$ with the incidence matrix ${{{\bf{U}}}}$**),** and lactation number (2 levels) and stage of lactation (3 levels) as fixed effects. To estimate the effects of the fixed effects on EME and the rumen metagenome features, we fitted linear models fitting EME and the KOs as the response variable, linear models explained by the same fixed effects used in the prediction models using the functions *asreml* and *wald.asreml* of the R package ASReml-R (version 3)^71^. Additionally, GBLUP models adjusted by the same effects were implemented to estimate the heritability of the ILR-transformed relative abundance of each of the 1032 KOs for each cattle population. Of the 410 rumen-sampled animals in Australia and the 432 in Spain, the BLUP models included 403 and 426 animals, respectively, because only these subsets had EME records and the additional effects required for model fitting.

The prediction models were conducted with the function *asreml* of the R package ASReml-R (version 3)^71^. The heritability (${h}^{2}$) was estimated with all models, except for MBLUP, as the proportion of the phenotypic variance explained by additive genetic variance. The microbiability (${m}^{2}$), a term introduced by Difford et al.^18^, was estimated with all models, except for GBLUP, as the proportion of the phenotypic variance explained by the ruminal microbiota. The portion of the phenotypic variance explained by the interaction between the host additive genetic effect and the ruminal microbiota effect (${i}^{2}$) was estimated with HiBLUP. The holobiability (${{ho}}^{2}$), a term introduced by Saborío-Montero et al.^10^, was estimated with HBLUP and with HiBLUP as the proportion of the phenotypic variance explained by the host additive genetics, the ruminal microbiota effect and the interaction between these two effects. Different estimated values (EV) were obtained. Analogously to the genomic estimated breeding value (GEBV) obtained from GRM, the estimated values from MRM were called estimated microbial value (EMV). When both GRM and MRM were included in the model, the estimated value from their interaction was called the estimated interaction value (EIV), and the estimated values from the whole model were called the estimated holobiont value (EHV). We calculated 95% confidence intervals using the standard normal approximation. This approach relies on the estimate and its standard error, assuming an approximately normal sampling distribution. The confidence interval defines the range within which the true value is expected to lie with 95% confidence. In cases where the lower bound of the interval was negative—due to sampling variability—the estimate was considered not statistically significant, as the percent variance explained is theoretically bounded at zero.

#### BayesR3 predictions

The EME microbiability was obtained with the BayesR approach to compare it with the microbiability obtained with the BLUP models. To do so, the microbiability was estimated from the variance explained by each relative-abundance CLR and ILR-transformed rumen metagenomic KO, scaled and centered across animals, using the program BayesR3^15^ (version released on May 28, 2025). The BayesR models were implemented without fitting a GRM. The log-ratio transformed KOs were centered and scaled across animals using the scale function in the R programming language^69^. The EME traits and fixed effects were the same as those previously mentioned in the BLUP models, except for the robot effects in Spain, which were included as fixed effects rather than random effects as in the BLUP models because the version of BayesR3 used does not allow it to be included it as an extra random effect. The MCMC procedure in BayesR3 estimated the number of KOs falling into four effect distributions: zero-effect $[N({{\mathrm{0,0.0}}}{{{{\rm{\sigma }}}}}_{{{{\rm{g}}}}}^{2})]$, small-effect$[N({{\mathrm{0,0.0001}}}{{{{\rm{\sigma }}}}}_{{{{\rm{g}}}}}^{2})]$, medium-effect $[N({{\mathrm{0,0.001}}}{{{{\rm{\sigma }}}}}_{{{{\rm{g}}}}}^{2})]$ and large-effect $[N({{\mathrm{0,0.01}}}{{{{\rm{\sigma }}}}}_{{{{\rm{g}}}}}^{2})]$. The initial (prior) proportions of variables assigned to each of the four effect components (zero-effect, small-effect, medium-effect, and large-effect) were 0.94, 0.049, 0.01, and 0.001, respectively. BayesR3 requires the definition of prior proportion of the phenotypic variance explained by the random predictors (the *-h* parameter), and we used the microbiability obtained in MBLUP of each population to define this prior. For variance component estimation, we used five chains and, following Martínez-Álvaro et al.^26^, we ran 1,000,000 iterations with a burn-in period of 200,000 iterations. The microbiability was obtained by dividing the variance from the KOs reported as ${V}_{a}$ (averaged across chains) by BayesR3 on the sum of this ${V}_{a}$ plus the error variance (${V}_{e}$; averaged across chains). Please note that the ${V}_{a}$ reported by BayesR3 in this case does not refer to the additive genetic variance as its name suggests.

#### Prediction accuracy

The accuracy of the estimated values was calculated with a 5-fold cross-validation approach within the population. In this accuracy estimation, the animals were initially randomly categorised into five groups. Each of the groups was used as a validation group by removing the phenotypes of the animals in that group and developing the prediction with the remaining four groups. In the BLUP models, the prediction accuracy was calculated as the correlation between the random coefficient regressors of the validation group and their EME corrected for fixed effects and the mean and standard deviation of the accuracies across the groups were calculated. For the BLUP models, this process was repeated 10 times, and the mean and standard deviations were averaged between repetitions to obtain the final accuracy mean and standard deviation.

The accuracy of the predictions obtained with BayesR was also estimated with a 5-fold cross-validation within population as the Pearson correlation between the estimated EMV of the validation set and their EME corrected for fixed effects, where the EMV was obtained by multiplying the CLR and ILR transformed relative abundance of each KO by its effect estimated in the reference sets. Additionally, the regression slope from adjusted EME on the estimated microbial values (b_EME, EMV_) was used to assess potential overfitting (b_EME, EMV_ < 1) or underfitting (b_EME, EMV_ > 1). These steps to calculate the accuracy and b_EME, EMV_ were repeated using each cohort as the validation set. The mean and standard deviation of the resulting accuracies and b_EME, EMV_ were reported. Due to computing limitations, this validation was performed using five chains with 50,000 iterations and a burn-in period of 25,000.

We also estimated the theoretical accuracy based on the number of animals with EME records, host genotypes, and ruminal metagenomics. According to Ross and Hayes^16^, the minimum accuracy of EMV obtained with MBLUP models could be estimated as follows:

$${r}_{{{{\rm{EMV}}}}}=\sqrt{\frac{{N}_{P}{m}^{2}}{{N}_{P}{m}^{2}+P}}$$

Where (${N}_{P}$) is the number of animals in the reference population with phenotype and metagenomics, ${m}^{2}$ is microbiability, and $P$ is the number of ruminal features (KOs). We extended this concept to estimate the accuracy of EHV obtained with HiBLUP. By doing this, we estimated the prediction accuracy according to the number of animals in the reference population that have EME records, host genomics, and ruminal metagenomics as follows:

$${r}_{{{{\rm{EHV}}}}}=\sqrt{\frac{{N}_{P}{{ho}}^{2}}{{N}_{P}{{ho}}^{2}+P}}$$

Where ${{ho}}^{2}$ is the holobiability obtained with HiBLUP. The standard error (SE) of this estimation was obtained as:

$${SE}=\sqrt{\frac{{r}_{{{{\rm{EHV}}}}}* (1-{r}_{{{{\rm{EHV}}}}})}{{Np}}}$$

This accuracy was estimated for a population size between 400 animals, which is approximately the current population size, and 10,000 animals.

### KEGG enrichment analysis

The KOs used in this study were included in a KEGG pathway analysis to identify overrepresented metabolic and functional categories. Enrichment analysis was performed using the enrichKEGG function of the clusterProfiler R package (v4.14.6)^72^ with the organism parameter set to “ko” to query against the full KEGG Orthology database. The background KO set was defined as the complete set of 14,385 KEGG Orthology terms available at the time of analysis (accessed on October 14, 2025)^14^. Statistical overrepresentation was evaluated using a hypergeometric test, which assesses whether the number of observed KOs in a given pathway exceeds the expected count by chance relative to the background, the obtained *p*-values were adjusted for multiple testing using the Benjamini–Hochberg method, and pathways with adjusted *p*-values (p.adjust) below 0.001 were considered statistically significant.

### Reporting summary

Further information on research design is available in the Nature Portfolio Reporting Summary linked to this article.

## Supplementary information

Transparent Peer Review file

Supplementary Information

Description of Additional Supplementary Files

Supplementary Data 1

Supplementary Data 2

Supplementary Data 3

Supplementary Data 4

Supplementary Data 5

Supplementary Data 6

Supplementary Code 1

Supplementary Code 2

Supplementary Code 3

Reporting Summary

## Data Availability

The rumen metagenome sequence reads and associated metadata of the Australian dataset are publicly available at the National Center for Biotechnology’s Sequence Read Archive, Bioproject accession PRJNA1162230. The data on enteric methane emissions, associated metadata, host genotypes, and other supplementary material from the Australian dairy cattle population were generated using animals from the Ellinbank Research Farm. However, these data were produced under formal agreements involving the State Government and dairy industry co‑investment. As a result, the data are subject to third‑party governance and cannot be made publicly available. Dairy Australia and Agriculture Victoria act as custodians of the data on behalf of the contributing parties. Reasonable requests for access for non‑commercial research purposes may be considered via Prof. Jennie E. Pryce, AgriBio, 5 Ring Road, Bundoora VIC 3083, Australia (jennie.pryce@agriculture.vic.gov.au), subject to approval by the custodians and execution of an appropriate Data Use Agreement. The rumen metagenome, methane measurements, and metadata for the Spanish population are available at the ENA under bioproject accession PRJNA789746 (https://www.ebi.ac.uk/ena/browser/view/PRJEB44278), the *GigaScience* database (https://gigadb.org/dataset/100950), and in López-García et al.^29^. The genotypes of the Spanish dairy cattle should be addressed to Dr. Óscar González-Recio, CSIC, Dpt Mejora Genética Animal, Crta. de La Coruña km 7.5, 28040 Madrid, Spain; E-mail: (gonzalez.oscar@inia.csic.es). Supplementary Data 4, 5, and **6** have the numerical values used to generate the graphs presented in Figs. 2, 3, and 4, respectively.

## Code availability

The code used to conduct the analyses and to generate the numerical source data required to reproduce the graphs and charts presented in the main figures is available at 10.5281/zenodo.19018891^73^. **Supplementary Code 1** has the code to generate descriptive figures, estimate variance components and prediction accuracy of enteric methane emissions with BLUP models, and estimation of reference population size. Due to commercial restrictions, the input data for **Supplementary Code 1** has the animal and sample IDs masked, and the genotypes, EME, metadata and fixed effects simulated; therefore, the results are not the same as those reported in the manuscript. **Supplementary Code 2** is the code to identify KOs that could be from *Bos taurus*. **Supplementary Code 3** is an example of the parameter file used as input in BayesR3^15^.
