# Supplementary material for: Reliable enteric methane prediction from the cattle (Bos taurus) rumen microbiome
Source: Commun Biol. 2026 Apr 13;9:810. doi: 10.1038/s42003-026-10048-8 (PMC13265817; doi:10.1038/s42003-026-10048-8)
Supplement: Supplementary file 2 — Supplementary Information [file 42003_2026_10048_MOESM2_ESM.pdf]

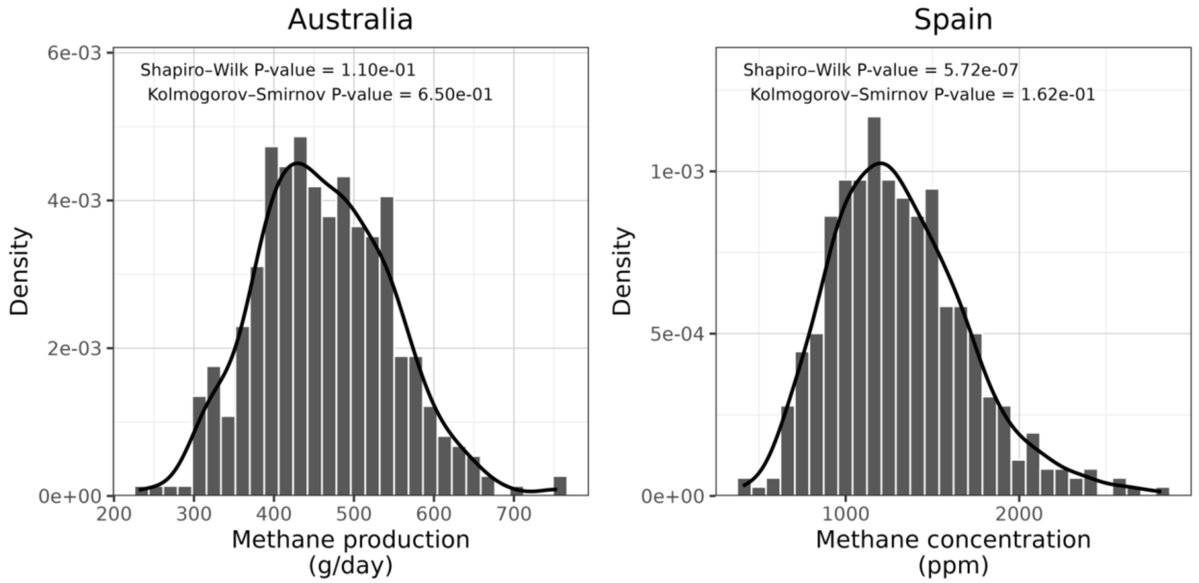

Supplementary Figure 1. Distribution and normality assessment of enteric methane emissions in two dairy cattle populations (Australia and Spain), based on histograms, density curves, and Shapiro–Wilk and Kolmogorov–Smirnov tests.

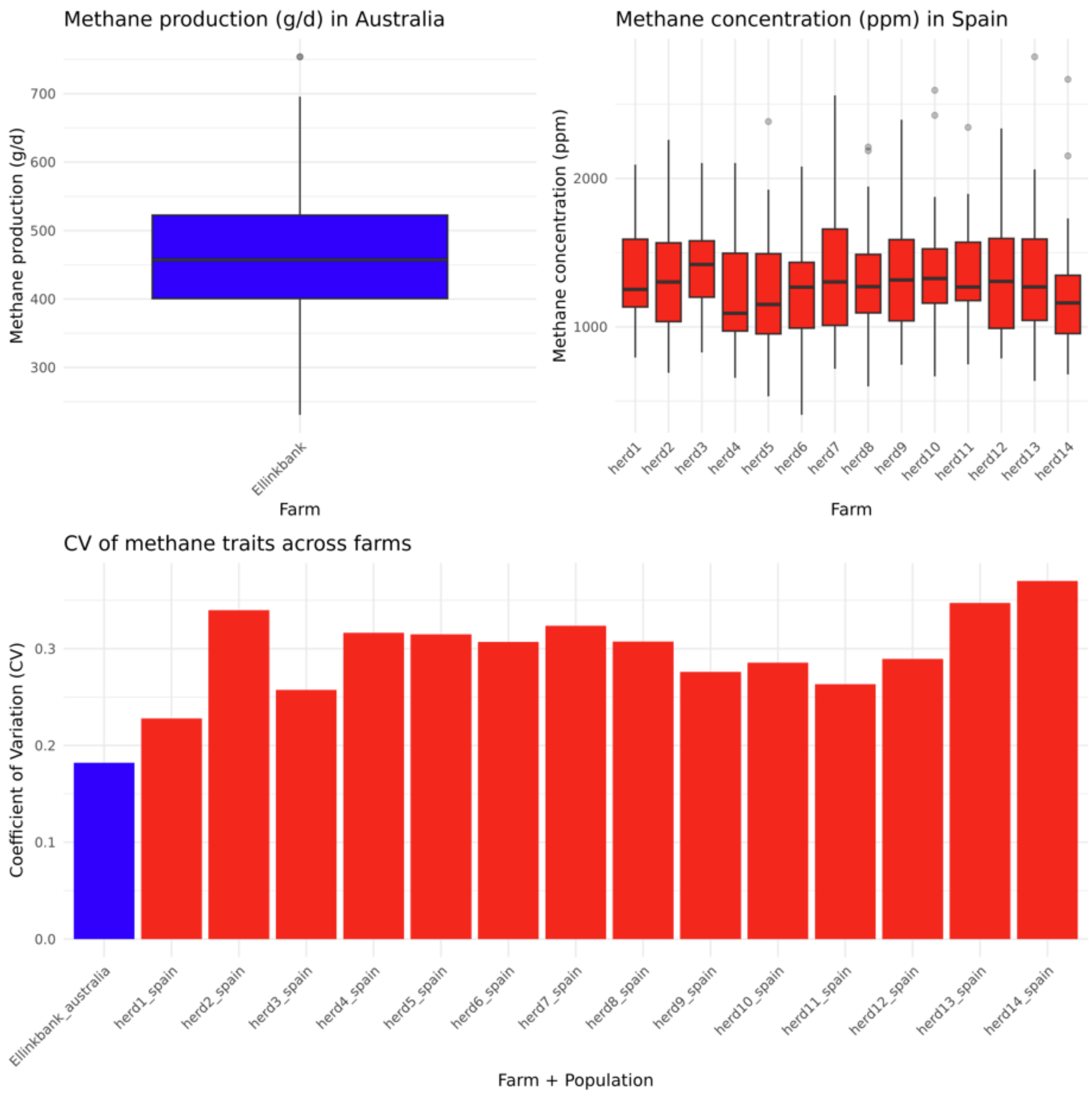

Supplementary Figure 2. Variation of methane emission traits across farms in two dairy cattle populations located in Australia and Spain. The farms are Ellinbank in Australia (blue) and herds 1 to 14 in Spain (red). The boxes of the boxplots represent interquartile ranges (IQR), horizontal lines indicate medians, whiskers extend to the smallest and largest values within 1.5×IQR, and points beyond whiskers denote outliers. The lower panel shows the coefficient of variation (CV) for enteric methane emissions across farms within each farm. Farm identifiers in the CV plot are labelled according to their respective populations (Australia or Spain) and coloured consistently with the top panels.

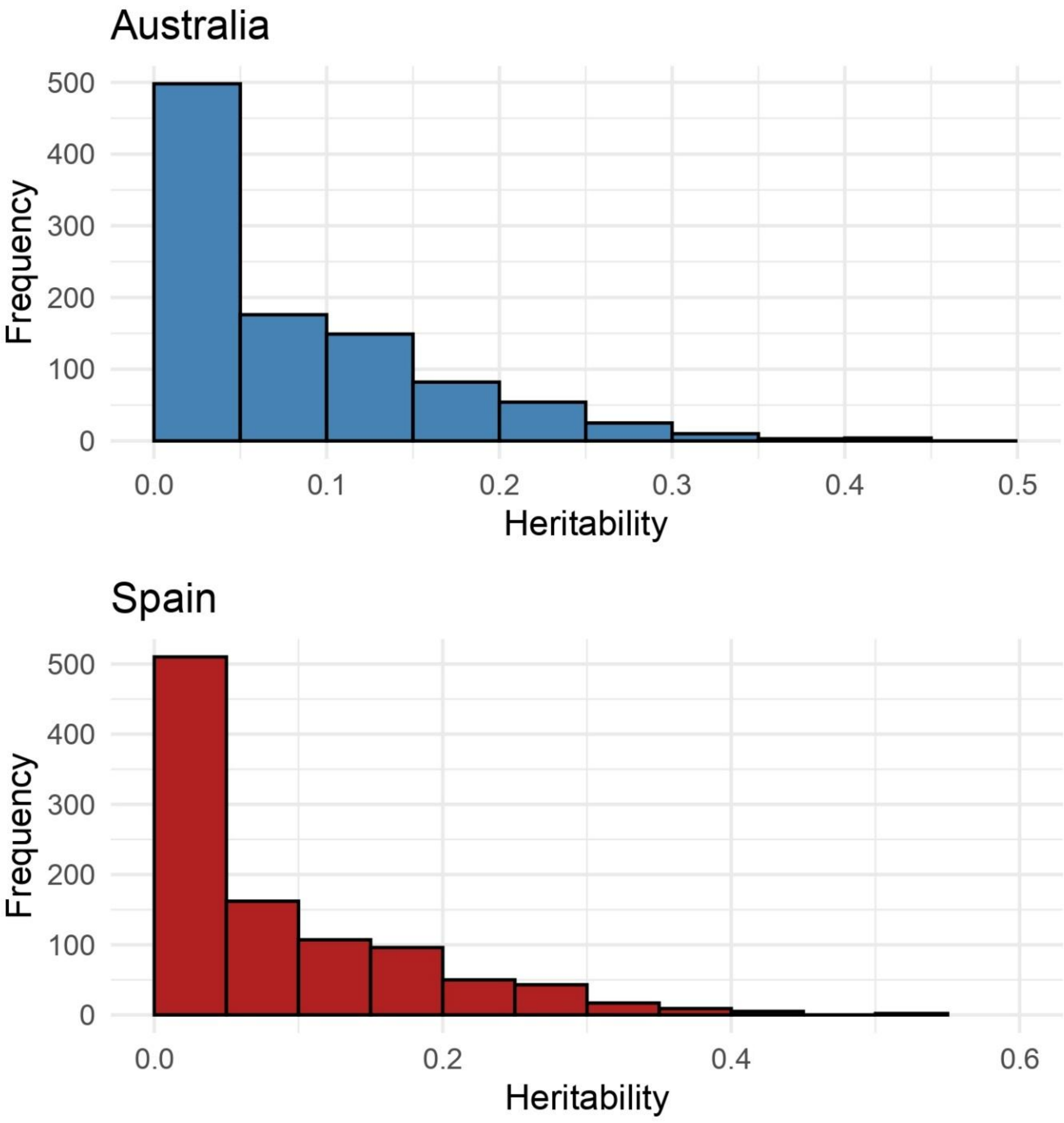

Supplementary Figure 3. Distribution of heritability of the core KEGG orthologs (KO) from the rumen metagenome of dairy cattle from Australia and Spain.

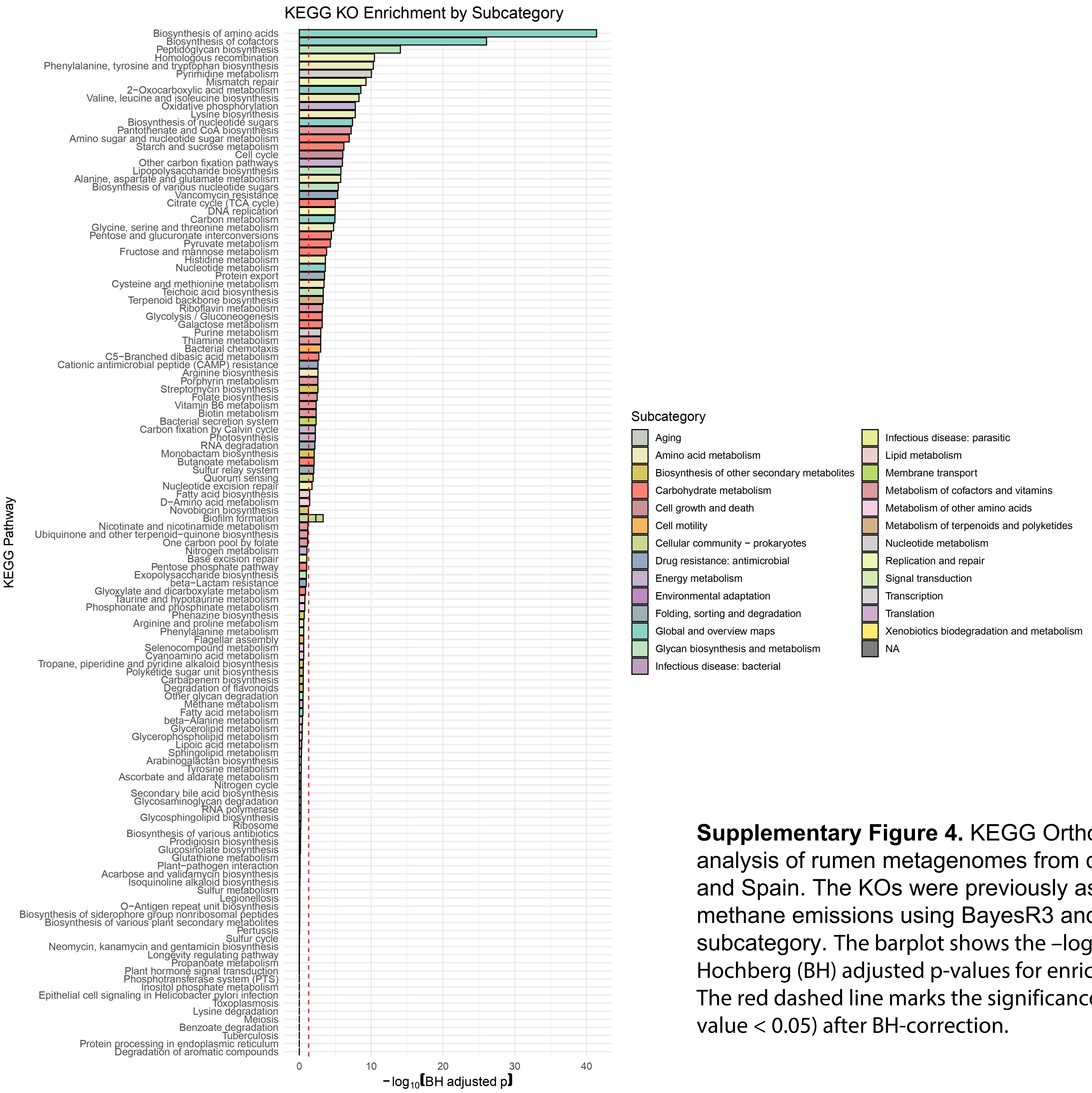

Supplementary Figure 4. KEGG Orthology (KO) enrichment analysis of rumen metagenomes from dairy cattle in Australia and Spain. The KOs were previously associated with enteric methane emissions using BayesR3 and grouped by functional subcategory. The barplot shows the $-\log_{10}$ of Benjamini–Hochberg (BH) adjusted p-values for enriched KEGG pathways. The red dashed line marks the significance threshold (adjusted p-value < 0.05) after BH-correction.

Supplementary Table 1. Wald tests for factors influencing methane production (g/day) in Australian and methane concentration (ppm) in the Spanish dairy cattle populations.

		Australia			
**Term**	**Df**	**Sum of Squares**	**Wald Statistic**	**P-value**	
Intercept	1	86268419	23019.8	< 2e-16	***
Cohort	10	903168	241	< 2e-16	***
Dry matter intake	1	479960	128.1	< 2e-16	***
Days in milk	1	12936	3.5	0.06319	.
Energy corrected milk	1	6452	1.7	0.18949	
daily body weight change	1	17607	4.7	0.03019	*
Residual		3748			
**Significance codes**: *** P ≤ 0.001; * P ≤ 0.05; . P ≤ 0.1					

		Spain			
**Term**	**Df**	**Sum of Squares**	**Wald Statistic**	**P-value**	
Intercept	1	741824021	4746.9	< 2e-16	***
Lactation number	1	225204	1.4	0.22997	
Stage of lactation	2	1268811	8.1	0.01726	*
Farm	13	1135604	7.3	0.88789	
Robot	4	710009	4.5	0.33745	
Residual		156277			
**Significance codes**: *** P ≤ 0.001; * P ≤ 0.05					

Supplementary Table 2. Variance components of enteric methane emission (EME) estimated with best linear unbiased predictions (BLUP) in two dairy cattle populations located in Australia and Spain along with the prediction accuracy of the models.

CLR												
Variance components of enteric methane emission											Cross-validation accuracy	
Country	Model	h^2	SE	m^2	SE	i^2	SE	ho^2	SE	Lower confidence bound	Upper confidence bound	mean	Standard deviation
Spain	GBLUP	0.11	0.10	0.00	0.00	0.00	0.00	0.00	0.00	0.00	0.31	0.07	0.09
Spain	MBLUP	0.00	0.00	0.08	0.06	0.00	0.00	0.00	0.00	0.00	0.20	0.16	0.12
Spain	HBLUP	0.10	0.10	0.09	0.06	0.00	0.00	0.19	0.11	0.00	0.41	0.15	0.09
Spain	HiBLUP	0.10	0.10	0.07	0.06	0.15	0.13	0.32	0.16	0.01	0.63	0.15	0.12
Australia	GBLUP	0.27	0.13	0.00	0.00	0.00	0.00	0.00	0.00	0.02	0.52	0.15	0.11
Australia	MBLUP	0.00	0.00	0.27	0.09	0.00	0.00	0.00	0.00	0.09	0.45	0.35	0.09
Australia	HBLUP	0.13	0.10	0.27	0.09	0.00	0.00	0.40	0.12	0.16	0.64	0.35	0.09
Australia	HiBLUP	0.14	0.10	0.24	0.09	0.17	0.12	0.55	0.15	0.26	0.84	0.36	0.09

ILR													
Variance components of enteric methane emission												Cross-validation accuracy	
Country	Model	h^2	SE	m^2	SE	i^2	SE	ho^2	SE	Lower confidence bound	Upper confidence bound	mean	Standard deviation
Spain	GBLUP	0.11	0.10	0.00	0.00	0.00	0.00	0.00	0.00	0.00	0.31	0.07	0.09
Spain	MBLUP	0.00	0.00	0.09	0.06	0.00	0.00	0.00	0.00	0.00	0.21	0.17	0.12
Spain	HBLUP	0.09	0.10	0.10	0.06	0.00	0.00	0.19	0.11	0.00	0.41	0.17	0.10
Spain	HiBLUP	0.10	0.10	0.08	0.05	0.16	0.13	0.34	0.16	0.03	0.65	0.18	0.12
Australia	GBLUP	0.27	0.13	0.00	0.00	0.00	0.00	0.00	0.00	0.02	0.52	0.15	0.11
Australia	MBLUP	0.00	0.00	0.31	0.09	0.00	0.00	0.00	0.00	0.13	0.49	0.39	0.08
Australia	HBLUP	0.08	0.09	0.30	0.09	0.00	0.00	0.38	0.12	0.14	0.62	0.39	0.10
Australia	HiBLUP	0.08	0.09	0.27	0.09	0.24	0.13	0.59	0.15	0.30	0.88	0.40	0.09

Difference between ILR and CLR													
Variance components of enteric methane emission												Cross-validation accuracy	
**Country**	**Model**	**h²**		**m²**		**i²**						**mean**	**Standard deviation**
Spain	MBLUP	0		0.01		0.00						0.01	
Spain	HBLUP	-0.01		0.01		0.00						0.02	
Spain	HiBLUP	0		0.01		0.01						0.03	
Australia	MBLUP	0		0.04		0.00						0.04	
Australia	HBLUP	-0.05		0.03		0.00						0.04	
Australia	HiBLUP	-0.06		0.03		0.07						0.04	

GBLUP: Host genomic BLUP obtained from a host genomic relationship matrix (GRM). MBLUP: Rumen microbiomic BLUP obtained from the microbiome relationship matrix (MRM) constructed with the Kyoto Encyclopedia of Genes and Genomes (KEGG) orthology groups (KOs) presented in the rumen metagenome. HBLUP: Hologenomic BLUP that combines the GRM and MRM. HiBLUP: Hologenomic BLUP with interaction, fitting the GRM, MRM and a third matrix accounting for the interaction between GRM and MRM. The heritability (h^2) was estimated with all models, except for MBLUP, as the proportion of the phenotypic variance explained by additive genetic variance. The microbiability (m^2), was estimated with all models, except for GBLUP, as the proportion of the phenotypic variance explained by the ruminal microbiota. The portion of the phenotypic variance explained by the interaction between the host additive genetic effect and the ruminal microbiota effect (i^2) was estimated with HiBLUP. The holobiability (ho^2), was estimated with HBLUP and with HiBLUP as the proportion of the phenotypic variance explained by the host additive genetics, the ruminal microbiota effect and the interaction between these two effects. The columns of h^2, m^2, i^2, and ho^2 are followed by their correspond standard error (SE). The lower and upper confidence bounds of the 95% confidence interval of the variance components was calculated as follows: Estimate − Z × Standard Error, where Z is the critical value for the 95% confidence level (approximately 1.96). The accuracy of the estimated values was estimated with a 10 repetition 5-fold cross-validation within population. CLR: center log-ratio transformation. ILR: isometric log-ratio transformation. 'Difference between ILR and CLR' shows the values of h^2, m^2 and i^2 obtained with ILR minus the respective value obtained with CLR. Therefore, positive numbers mean that ILR's values are higher and negative values show that CLR's values are higher.
