# Supplementary material for: Reliable enteric methane prediction from the cattle (Bos taurus) rumen microbiome
Source: Commun Biol. 2026 Apr 13;9:810. doi: 10.1038/s42003-026-10048-8 (PMC13265817; doi:10.1038/s42003-026-10048-8)
Supplement: Supplementary file 3 — Description of Additional Supplementary Files [file 42003_2026_10048_MOESM3_ESM.pdf]

Description of Additional Supplementary Files

File name: Supplementary Data 1
Description: Classification of KEGG orthologs (KO) from the rumen metagenome based on their associated microbial biological processes, as delineated in the KEGG database consulted in May 2024.

File name: Supplementary Data 2
Description: Top 100 KEGG orthologs (KO) from the rumen metagenome with higher effect on enteric methane emissions using microbiomic best linear unbiased prediction (MBLUP) in two dairy cattle populations located in Australia and Spain.

File name: Supplementary Data 3
Description: KEGG pathways significantly represented by the 1,032 KEGG orthologs (KO) identifiers used to estimate the variance of enteric methane emissions (Benjamini–Hochberg adjusted $p < 0.001$).

File name: Supplementary Data 4
Description: Numerical values used to generate the graphs presented in Figure 2.

File name: Supplementary Data 5
Description: Numerical values used to generate the graphs presented in Figure 3.

File name: Supplementary Data 6
Description: Numerical values used to generate the graphs presented in Figure 4.

File name: Supplementary Code 1
Description: code to generate descriptive figures, estimate variance components and prediction accuracy of enteric methane emissions with BLUP models, and estimation of reference population size. Due to commercial restrictions, the input data for **Supplementary Code 1** has the animal and sample IDs masked, and the genotypes, EME, metadata and fixed effects simulated, and therefore the results are not the same as those reported in the manuscript.

File name: Supplementary Code 2
Description: code to identify KOs that could be from *Bos taurus*.

File name: Supplementary Code 3
Description: example of the parameter file used as input in BayesR3

Unofficial
