# Supplementary material for: Reliable enteric methane prediction from the cattle (Bos taurus) rumen microbiome
Source: Commun Biol. 2026 Apr 13;9:810. doi: 10.1038/s42003-026-10048-8 (PMC13265817; doi:10.1038/s42003-026-10048-8)
Supplement: Supplementary file 10 — Supplementary Code 1 [file 42003_2026_10048_MOESM10_ESM.zip › Supplementary Code 1.nb.html]

Reliable enteric methane predictor from the cattle rumen microbiome


Code 

- Show All Code
- Hide All Code
- Download Rmd

# Reliable enteric methane predictor from the cattle rumen microbiome

# Define parameters manually


```
# Define "Australia" or "Spain"
population = "Australia"

# Define the type of logratio transformation
log_transformation = 'ilr' # 'clr', 'alr', 'ilr'

# To run the code including the cross-validation, run the line below.
# It takes a long time to run
# rm(corsVal)
```

# Load data and dependencies

# Differences in methane emissions across farms


```
has_asreml <- requireNamespace("asreml", quietly = TRUE)
if (!has_asreml) {
  warning("The package 'asreml' is not installed. Some functions may not work properly.")
}

# Australia
data_tmp = metadata[metadata$population == 'australia',
                    which(colnames(metadata) %in% c('MeP.x' , 'Cohort' , 'dmi' , 'DIM' , 'mECM' , 'LWTdelta'))]
data_tmp = data_tmp[complete.cases(data_tmp),]
if (has_asreml) {
  fixed_effect_model_australia <-
    asreml::asreml(MeP.x ~ 1 + as.factor(Cohort) + dmi + DIM + mECM + LWTdelta, data = data_tmp)
  asreml::wald.asreml(fixed_effect_model_australia)
}

# Spain
data_tmp = metadata[metadata$population == 'spain',
                    which(colnames(metadata) %in% c('MeC' , 'lactation_number' , 'stage_of_lactation' , 'farm' , 'robot'))]
data_tmp$farm <- as.factor(data_tmp$farm)
data_tmp$robot <- as.factor(data_tmp$robot)
data_tmp$stage_of_lactation <- as.factor(data_tmp$stage_of_lactation)
if (has_asreml) {
  fixed_effect_model_spain <-
    asreml::asreml(MeC ~ 1 + lactation_number + stage_of_lactation + farm + robot,
                   data = data_tmp)
  asreml::wald.asreml(fixed_effect_model_spain)
}

# ////////////////////////////
# Plots 
# ////////////////////////////

# Subset relevant columns
data_tmp <- metadata %>% 
  select(population, MeP.x, MeC, farm)

# Long format and relabel traits
data_long <- data_tmp %>%
  pivot_longer(cols = c(MeP.x, MeC), names_to = "trait", values_to = "value") %>%
  filter(!is.na(value)) %>%
  mutate(
    trait_label = case_when(
      trait == "MeP.x" ~ "Methane production (g/d) in Australia",
      trait == "MeC"   ~ "Methane concentration (ppm) in Spain"
    ),
    trait_label = factor(trait_label, levels = c(
      "Methane production (g/d) in Australia",
      "Methane concentration (ppm) in Spain"
    ))
  )

# Create separate subsets explicitly for plots
data_aus <- data_long %>%
  filter(population == "australia", trait == "MeP.x") %>%
  mutate(farm = factor(farm, levels = str_sort(unique(farm), numeric = TRUE)))

data_spain <- data_long %>%
  filter(population == "spain", trait == "MeC") %>%
  mutate(farm = factor(farm, levels = str_sort(unique(farm), numeric = TRUE)))

# Plot Methane Production in Australia (Only Ellinbank)
p1 <- ggplot(data_aus, aes(x = farm, y = value)) +
  geom_boxplot(outlier.alpha = 0.3, fill = "blue") +
  theme_minimal() +
  theme(
    axis.text.x = element_text(angle = 45, hjust = 1)
  ) +
  labs(
    y = "Methane production (g/d)",
    x = "Farm",
    title = "Methane production (g/d) in Australia"
  )

# Plot: Methane Concentration in Spain
p2 <- ggplot(data_spain, aes(x = farm, y = value)) +
  geom_boxplot(outlier.alpha = 0.3, fill = "red") +
  theme_minimal() +
  theme(
    axis.text.x = element_text(angle = 45, hjust = 1)
  ) +
  labs(
    y = "Methane concentration (ppm)",
    x = "Farm",
    title = "Methane concentration (ppm) in Spain"
  )

# Combine plots
combined_p1_p2 <- p1 + p2 + plot_layout(ncol = 2)

# Plot Coefficient of Variation 
cv_df <- bind_rows(data_aus, data_spain) %>%
  group_by(population, farm, trait_label) %>%
  summarise(
    mean = mean(value, na.rm = TRUE),
    sd = sd(value, na.rm = TRUE),
    cv = sd / mean,
    .groups = "drop"
  ) %>%
  mutate(
    farm_pop = paste(farm, population, sep = "_"),
    farm_pop = factor(farm_pop, levels = str_sort(unique(farm_pop), numeric = TRUE))
  )

p3 <- ggplot(cv_df, aes(x = farm_pop, y = cv, fill = trait_label)) +
  geom_col() +
  scale_fill_manual(values = c(
    "Methane production (g/d) in Australia" = "blue",
    "Methane concentration (ppm) in Spain" = "red"
  )) +
  theme_minimal() +
  theme(
    axis.text.x = element_text(angle = 45, hjust = 1),
    legend.position = "none"
  ) +
  labs(
    y = "Coefficient of Variation (CV)",
    x = "Farm + Population",
    title = "CV of methane traits across farms"
  )

# Combine all plots
final_plot <- combined_p1_p2 / p3
final_plot
```


```
NA
```

# Genomic relationship matrix (GRM) and its inverse


```
if(!exists("inverseGRM")){
  grmAndInv <- genos2ginv(genotypes = genotype, method = "Yang", maf = 0.05)
  grm <- grmAndInv$GRM
  inverseGRM <- grmAndInv$ginv
}
```

# Microbial relationship matrix (MRM)

Metagenome data transformation


```
# Prevalence (P) filter.
prev.aus <- aa.aus[apply(aa.aus, 1, function(x) is_in_most_samples(x, p = 100)), ]
prev.spa <- aa.spa[apply(aa.spa, 1, function(x) is_in_most_samples(x, p = 100)), ]

# Remove KOs no presented in all animals of both populations
common_KOs <- rownames(prev.aus)[rownames(prev.aus)%in%rownames(prev.spa)]
prev.aus <- prev.aus[common_KOs,]
prev.spa <- prev.spa[common_KOs,]

# Relative abundance
ra.aus <- sweep(prev.aus, 2, colSums(prev.aus), FUN = "/") 
ra.spa <- sweep(prev.spa, 2, colSums(prev.spa), FUN = "/") 

# Transpose of RA
B.aus <- t(ra.aus)
B.spa <- t(ra.spa)

# ILR transformation
if(log_transformation == 'clr'){
  lt.aus <- easyCODA::CLR(B.aus, weight = FALSE)
  lt.aus <- lt.aus$LR
  lt.spa <- easyCODA::CLR(B.spa, weight = FALSE)
  lt.spa <- lt.spa$LR
}
if(log_transformation == 'alr'){
  lt.aus <- easyCODA::ALR(B.aus, weight = FALSE)
  lt.aus <- lt.aus$LR
  lt.spa <- easyCODA::ALR(B.spa, weight = FALSE)
  lt.spa <- lt.spa$LR
}
if(log_transformation == 'ilr'){
  lt.aus <- as.matrix(compositions::ilr(B.aus))
  lt.spa <- as.matrix(compositions::ilr(B.spa))
}

  
# Scale and center KOs across animals
lt.aus <- scale(lt.aus) 
lt.spa <- scale(lt.spa)
```


Comparison of KO abundances between countries


```
# CLR transformation
clr.aus <- easyCODA::CLR(B.aus, weight = FALSE)
clr.aus <- clr.aus$LR
clr.spa <- easyCODA::CLR(B.spa, weight = FALSE)
clr.spa <- clr.spa$LR

# Ensure both matrices have the same KOs and in the same order
kos <- intersect(colnames(clr.aus), colnames(clr.spa))
n_kos <- length(kos)

#####################################
# Estimate significance of differences between countries in terms of CLR-transformed KOs
#####################################
results <- data.frame(
  KO        = kos,
  p_value   = NA_real_,
  mean_AUS  = NA_real_,
  mean_SPA  = NA_real_,
  diff      = NA_real_,
  stringsAsFactors = FALSE
)

for (i in seq_len(n_kos)) {
  ko <- kos[i]

  # Extract CLR-transformed values as vectors
  x <- clr.aus[, ko]  
  y <- clr.spa[, ko]  

  # Means
  results$mean_AUS[i] <- mean(x, na.rm = TRUE)
  results$mean_SPA[i] <- mean(y, na.rm = TRUE)
  results$diff[i]     <- results$mean_AUS[i] - results$mean_SPA[i]

  # Wilcoxon rank-sum test
  wt <- wilcox.test(x, y, exact = FALSE) 
  results$p_value[i] <- wt$p.value
}

# FDR correction
results$q_value <- p.adjust(results$p_value, method = "fdr")

# Order by q-value
results <- results[order(results$q_value), ]

# ABSOLUTE ABUNDANCE OF KOs IN AUSTRALIA
write.table(results, file = "./ILR-transformed_KOs_differences.txt", row.names = F, col.names = T, quote = F, sep = "\t") 


#####################################
# Correlation CLR-abundances between countries
#####################################
mean_aus <- colMeans(clr.aus)
mean_spa <- colMeans(clr.spa)

plot_df <- data.frame(
  KO = names(mean_aus),
  AUS = mean_aus,
  SPA = mean_spa
)

fit <- lm(SPA ~ AUS, data = plot_df)
R2 <- round(summary(fit)$r.squared,2)

library(ggplot2)

ggplot(plot_df, aes(x = AUS, y = SPA)) +
  geom_point(alpha = 0.5, size = 2, color = "steelblue") +
  geom_abline(slope = 1, intercept = 0, linetype = "dashed", color = "gray50") +
  geom_smooth(method = "lm", se = FALSE, color = "firebrick", linewidth = 1) +
  annotate(
    "text",
    x = min(plot_df$AUS),
    y = max(plot_df$SPA),
    hjust = 0, vjust = 1,
    label = paste0("R² = ", round(R2, 3)),
    size = 5, color = "firebrick"
  ) +
  labs(
    x = "Mean CLR abundance (Australia)",
    y = "Mean CLR abundance (Spain)",
    title = "Comparison of KO abundances between countries"
  ) +
  theme_bw(base_size = 14)
```


Use the selected population


```
if(population == "Australia"){ 
 lt <- lt.aus
 aa.pop <- aa.aus
 B = B.aus
} else if(population == "Spain"){
 lt <- lt.spa
 aa.pop <- aa.spa
 B = B.spa
}
```


Create the microbial relationship matrix (MRM) and its inverse


```
# Cross-product matrix
crpr <- lt %*% t(lt)

mrm <- (1 / nrow(aa.pop)) * crpr

# Add a small value to the main diagonal because it's singular
mrm <- mrm + diag(1e-8, nrow(mrm))
rownames(mrm) <- rownames(crpr)
colnames(mrm) <- colnames(crpr)

# Inverse of MRM
inverseMRM <- mrmInvFunc(mrm)

# Add a identifier of the rumen metagenomes (MEID) to the metadata
B_for_addMEID2phen <- data.table(row.names(B), B)
row.names(B_for_addMEID2phen) <- c(1:nrow(B_for_addMEID2phen))
metagenome_sample_in_phen <- which(colnames(metadata) == "Animal_ID")
new_col_order <- c(metagenome_sample_in_phen, setdiff(1:ncol(metadata), metagenome_sample_in_phen))
metadata <- metadata[, new_col_order]
metadata <- addMEID2phen(metagenomes = B_for_addMEID2phen, phenotype_and_metadata = metadata) #VA! Issue adding MEID
```

# Interaction between GRM and MRM


```
hadGrmMrm <- adamardGrmMrm(GRM = grm, MRM = mrm, metadata = metadata, genotype = genotype)[[1]]
```


```
Error in data.frame(row = row.names(tmp_data_model_geid_meid), Hadamard = c(1:nrow(tmp_data_model_geid_meid))) : 
  arguments imply differing number of rows: 0, 2
```

# Run prediction models


```
metadata$GEID <- as.factor(metadata$GEID)
metadata$MEID <- as.factor(metadata$MEID)
metadata$hadGrmMrm <- as.factor(metadata$hadGrmMrm)
metadata$farm <- as.factor(metadata$farm)
metadata$robot <- as.factor(metadata$robot)
metadata$stage_of_lactation <- as.factor(metadata$stage_of_lactation)
if(population == 'Australia'){EME = 'MeP.x'}
if(population == 'Spain'){EME = 'MeC'}
if (has_asreml) {
  asremlModels <- list()
  for (model in c("HiBLUP", "HBLUP", "MBLUP", "GBLUP")) {
    print(paste0(
      "Start population ",
      population,
      ", trait: ",
      EME,
      ", and model ",
      model
    ))
    asremlModels[[population]][[model]] <-
      do.call(asreml,
              asreml.args(
                population = population,
                y = EME,
                model = model
              ))
  }
} else{
  message("Skipping ASReml models because 'asreml' is not installed.")
}
```

# Estimation of variance components


```
if (has_asreml) {
  if (population == "Australia") {
    varcomp_Australia <- data.frame(matrix(data <- NA, nrow = 4, ncol = 4))
    rownames(varcomp_Australia) <- c("HiBLUP", "HBLUP", "MBLUP", "GBLUP")
    colnames(varcomp_Australia) <- c("h2", "m2", "i2", "ho2")
    
    # HiBLUP Australia
    summary(asremlModels$Australia$HiBLUP)$varcomp # variance components of HiBLUP Australia
    varcomp_Australia["HiBLUP", "m2"] <-
      pasteStatsAndSE_pin(round(
        nadiv::pin(asremlModels$Australia$HiBLUP, m2 ~ V1 / (V1 + V2 + V3 + V4)),
        digits = 2
      ))
    varcomp_Australia["HiBLUP", "i2"] <-
      pasteStatsAndSE_pin(round(
        nadiv::pin(asremlModels$Australia$HiBLUP, i2 ~ V2 / (V1 + V2 + V3 + V4)),
        digits = 2
      ))
    varcomp_Australia["HiBLUP", "h2"] <-
      pasteStatsAndSE_pin(round(
        nadiv::pin(asremlModels$Australia$HiBLUP, h2 ~ V3 / (V1 + V2 + V3 + V4)),
        digits = 2
      ))
    varcomp_Australia["HiBLUP", "ho2"] <-
      pasteStatsAndSE_pin(round(
        nadiv::pin(
          asremlModels$Australia$HiBLUP,
          ho2 ~ (V1 + V2 + V3) / (V1 + V2 + V3 + V4)
        ),
        digits = 2
      ))
    
    # HBLUP Australia
    summary(asremlModels$Australia$HBLUP)$varcomp # variance components of HBLUP Australia
    varcomp_Australia["HBLUP", "m2"] <-
      pasteStatsAndSE_pin(round(
        nadiv::pin(asremlModels$Australia$HBLUP, m2 ~ V1 / (V1 + V2 + V3)),
        digits = 2
      ))
    varcomp_Australia["HBLUP", "h2"] <-
      pasteStatsAndSE_pin(round(
        nadiv::pin(asremlModels$Australia$HBLUP, h2 ~ V2 / (V1 + V2 + V3)),
        digits = 2
      ))
    varcomp_Australia["HBLUP", "ho2"] <-
      pasteStatsAndSE_pin(round(
        nadiv::pin(asremlModels$Australia$HBLUP, ho2 ~ (V1 + V2) / (V1 + V2 + V3)),
        digits = 2
      ))
    
    # MBLUP Australia
    summary(asremlModels$Australia$MBLUP)$varcomp # variance components of MBLUP Australia
    varcomp_Australia["MBLUP", "m2"] <-
      pasteStatsAndSE_pin(round(
        nadiv::pin(asremlModels$Australia$MBLUP, m2 ~ V1 / (V1 + V2)),
        digits = 2
      ))
    
    # GBLUP Australia
    summary(asremlModels$Australia$GBLUP)$varcomp # variance components of GBLUP Australia
    varcomp_Australia["GBLUP", "h2"] <-
      pasteStatsAndSE_pin(round(
        nadiv::pin(asremlModels$Australia$GBLUP, h2 ~ V1 / (V1 + V2)),
        digits = 2
      ))
    
    # Print varcomps in Australia
    varcomp_Australia
    
  } else if (population == "Spain") {
    varcomp_Spain <- data.frame(matrix(data <- NA, nrow = 4, ncol = 4))
    rownames(varcomp_Spain) <- c("HiBLUP", "HBLUP", "MBLUP", "GBLUP")
    colnames(varcomp_Spain) <- c("h2", "m2", "i2", "ho2")
    
    # HiBLUP Spain
    summary(asremlModels$Spain$HiBLUP)$varcomp # variance components of HiBLUP Spain
    varcomp_Spain["HiBLUP", "m2"] <-
      pasteStatsAndSE_pin(round(nadiv::pin(
        asremlModels$Spain$HiBLUP, m2 ~ V2 / (V1 + V2 + V3 + V4 + V5)
      ), digits = 2))
    varcomp_Spain["HiBLUP", "i2"] <-
      pasteStatsAndSE_pin(round(nadiv::pin(
        asremlModels$Spain$HiBLUP, i2 ~ V3 / (V1 + V2 + V3 + V4 + V5)
      ), digits = 2))
    varcomp_Spain["HiBLUP", "h2"] <-
      pasteStatsAndSE_pin(round(nadiv::pin(
        asremlModels$Spain$HiBLUP, h2 ~ V4 / (V1 + V2 + V3 + V4 + V5)
      ), digits = 2))
    varcomp_Spain["HiBLUP", "ho2"] <-
      pasteStatsAndSE_pin(round(nadiv::pin(
        asremlModels$Spain$HiBLUP,
        ho2 ~ (V2 + V3 + V4) / (V1 + V2 + V3 + V4 + V5)
      ), digits = 2))
    
    # HBLUP Spain
    summary(asremlModels$Spain$HBLUP)$varcomp # variance components of HBLUP Spain
    varcomp_Spain["HBLUP", "m2"] <-
      pasteStatsAndSE_pin(round(nadiv::pin(
        asremlModels$Spain$HBLUP, m2 ~ V2 / (V1 + V2 + V3 + V4)
      ), digits = 2))
    varcomp_Spain["HBLUP", "h2"] <-
      pasteStatsAndSE_pin(round(nadiv::pin(
        asremlModels$Spain$HBLUP, h2 ~ V3 / (V1 + V2 + V3 + V4)
      ), digits = 2))
    varcomp_Spain["HBLUP", "ho2"] <-
      pasteStatsAndSE_pin(round(nadiv::pin(
        asremlModels$Spain$HBLUP, ho2 ~ (V2 + V3) / (V1 + V2 + V3 + V4)
      ), digits = 2))
    
    # MBLUP Spain
    summary(asremlModels$Spain$MBLUP)$varcomp # variance components of MBLUP Spain
    varcomp_Spain["MBLUP", "m2"] <-
      pasteStatsAndSE_pin(round(nadiv::pin(
        asremlModels$Spain$MBLUP, m2 ~ V2 / (V1 + V2 + V3)
      ), digits = 2))
    
    # GBLUP Spain
    summary(asremlModels$Spain$GBLUP)$varcomp # variance components of GBLUP Spain
    varcomp_Spain["GBLUP", "h2"] <-
      pasteStatsAndSE_pin(round(nadiv::pin(
        asremlModels$Spain$GBLUP, h2 ~ V2 / (V1 + V2 + V3)
      ), digits = 2))
    
    # Print varcomps in Spain
    varcomp_Spain
  }
} else{
  message("Skipping variance components estimation because 'asreml' is not installed.")
}
```

# Estimation of prediction accuracy as a 10-repeated 5-fold cross-validation


```
if (has_asreml) {
  if (!exists("corsVal")) {
    corsVal <- list()
    for (model in c("HiBLUP", "HBLUP", "MBLUP", "GBLUP")) {
      for (repetition in 1:10) {
        print(
          paste0(
            "Start population ",
            population,
            "; model ",
            model,
            "; repetition ",
            repetition,
            " of 10"
          )
        )
        corsVal[[population]][[model]][[repetition]] <- xvalRandomAsreml.v02(population = population, model = model)
      }
    }
    
    # Average between repetitions
    m <- c()
    sd <- c()
    accuracy <- data.frame(matrix(
      data = NA,
      nrow = 8,
      ncol = 4
    ))
    colnames(accuracy) <- c("population", "model", "mean", "sd")
    accuracy$population <- rep(c("Australia", "Spain"), each = 4)
    accuracy$model <- rep(c("HiBLUP", "HBLUP", "MBLUP", "GBLUP"), 2)
    for (model in c("HiBLUP", "HBLUP", "MBLUP", "GBLUP")) {
      for (r in 1:10) {
        m[r] <- splitStatsAndDeviation(corsVal[[population]][[model]][[r]])[[1]]
        sd[r] <- splitStatsAndDeviation(corsVal[[population]][[model]][[r]])[[2]]
      }
      accuracy[accuracy$population == population &
                 accuracy$model == model, "mean"] <-
        round(mean(unlist(m)), 2)
      accuracy[accuracy$population == population &
                 accuracy$model == model, "sd"] <-
        round(mean(unlist(sd)), 2)
    }
    
    print(accuracy)
  } else {
    print(
      paste(
        "Cross-validation has not been executed to avoid overwriting a previous result, which likely required a significant amount of time to complete. If you wish to overwrite a previous cross-validation result, please remove it using rm(corsVal) and attempt this section again."
      )
    )
  }
} else{
    message("Skipping prediction accuracy estimation because 'asreml' is not installed.")
}
```

# Estimation of Reference Population Size


```
# Estimation of reference population for both populations
rEHV_Australia <- data.frame(Np = integer(), Accuracy = numeric(), SE = numeric())
rEHV_Spain <- data.frame(Np = integer(), Accuracy = numeric(), SE = numeric())

# Loop through reference population sizes
for (Np in 1:10000) {
  result_Australia <- theoreticalHolobionPredicitonAccuracy2(P = 1032, ho2 = 0.59, Np = Np)
  rEHV_Australia <- rbind(rEHV_Australia, data.frame(Np = Np, Accuracy = result_Australia$Accuracy, SE = result_Australia$SE))
}
for (Np in 1:10000) {
  result_Spain <- theoreticalHolobionPredicitonAccuracy2(P = 1032, ho2 = 0.34, Np = Np)
  rEHV_Spain <- rbind(rEHV_Spain, data.frame(Np = Np, Accuracy = result_Spain$Accuracy, SE = result_Spain$SE))
}

# Add population labels
rEHV_Australia$Population <- "Australia"
rEHV_Spain$Population <- "Spain"
rEHV_both <- rbind(rEHV_Australia, rEHV_Spain)

# Plot with error ribbons
ggplot(data = rEHV_both, aes(x = Np, y = Accuracy, color = Population)) +
  geom_line(size = 0.1) +
  geom_ribbon(aes(ymin = Accuracy - 1.96 * SE, ymax = Accuracy + 1.96 * SE, fill = Population), alpha = 0.2) +
  geom_vline(xintercept = 400,  linetype = "dashed") +
  geom_vline(xintercept = 6000, linetype = "dashed") +
  geom_hline(yintercept = 0.45, linetype = "dashed", color = "blue") +
  geom_hline(yintercept = 0.35, linetype = "dashed", color = "red") +
  scale_color_manual(values = c("blue", "red")) +
  scale_fill_manual(values = c("blue", "red")) +
  labs(x = "Reference population size", y = "Prediction accuracy",
       title = "Theoretical accuracy of estimated holobiont value with error bars") +
  scale_y_continuous(
    breaks = unique(c(0, 0.10, 0.20, 0.35, 0.45, 0.60, 0.70, 0.80, 0.90, 1)),
    limits = c(0, 1)
  ) +
  scale_x_continuous(
    breaks = unique(c(400, 2500, 5000, 6000, 7500, 10000)),
    limits = c(0, 10000),
    labels = scales::comma
  ) +
  theme_minimal()
```


```
NA
NA
```

LS0tDQp0aXRsZTogIlJlbGlhYmxlIGVudGVyaWMgbWV0aGFuZSBwcmVkaWN0b3IgZnJvbSB0aGUgY2F0dGxlIHJ1bWVuIG1pY3JvYmlvbWUiDQpvdXRwdXQ6DQogIHBkZl9kb2N1bWVudDogZGVmYXVsdA0KICBodG1sX25vdGVib29rOiBkZWZhdWx0DQotLS0NCg0KIyBEZWZpbmUgcGFyYW1ldGVycyBtYW51YWxseQ0KYGBge3J9DQojIERlZmluZSAiQXVzdHJhbGlhIiBvciAiU3BhaW4iDQpwb3B1bGF0aW9uID0gIkF1c3RyYWxpYSINCg0KIyBEZWZpbmUgdGhlIHR5cGUgb2YgbG9ncmF0aW8gdHJhbnNmb3JtYXRpb24NCmxvZ190cmFuc2Zvcm1hdGlvbiA9ICdpbHInICMgJ2NscicsICdhbHInLCAnaWxyJw0KDQojIFRvIHJ1biB0aGUgY29kZSBpbmNsdWRpbmcgdGhlIGNyb3NzLXZhbGlkYXRpb24sIHJ1biB0aGUgbGluZSBiZWxvdy4NCiMgSXQgdGFrZXMgYSBsb25nIHRpbWUgdG8gcnVuDQojIHJtKGNvcnNWYWwpDQpgYGANCg0KDQojIExvYWQgZGF0YSBhbmQgZGVwZW5kZW5jaWVzDQpgYGB7cn0NCg0KIyBXb3JraW5nIGRpcmVjdG9yeQ0Kc2V0d2QoZGlybmFtZShyc3R1ZGlvYXBpOjpnZXRBY3RpdmVEb2N1bWVudENvbnRleHQoKSRwYXRoKSkNCg0KIyBNRVRBREFUQSBBTkQgTUVUSEFORSBFTUlTU0lPTlMNCiMNCiMgKiBDb21tb24gY29sdW1ucyBpbiBBdXN0cmFsaWEgYW5kIFNwYWluDQojIEFuaW1hbF9JRA0KIyBzYW1wbGVfSUQ6IEhvc3QgZ2VuZXRpYyBzYW1wbGUgSUQNCiMgcG9wdWxhdGlvbg0KIyBHRUlEOiBBbmltYWwgSUQgaW4gdGhlIGdlbm9taWMgcmVsYXRpb25zaGlwIG1hdHJpeCAoR1JNKQ0KIyBNRUlEOiBBbmltYWwgSUQgaW4gdGhlIG1pY3JvYmlhbCByZWxhdGlvbnNoaXAgbWF0cml4IChNUk0pDQojIGhhZEdybU1ybTogQW5pbWFsIElEIGluIHRoZSBtYXRyaXggb2YgdGhlIGludGVyYWN0aW9uIGJldHdlZW4gR1JNIGFuZCBNUk0NCiMgDQojICogQ29sdW1ucyBzcGVjaWZpYyB0byBBdXN0cmFsaWENCiMgTWVQLng6IE1ldGhhbmUgcHJvZHVjdGlvbg0KIyBDb2hvcnQNCiMgZG1pOiBkcnkgbWF0dGVyIGludGFrZQ0KIyBESU06IGRheXMgaW4gbWlsaw0KIyBtRUNNOiBlbmVyZ3kgY29ycmVjdGVkIG1pbGsNCiMgTFdUZGVsdGE6IGRhaWx5IGJvZHkgd2VpZ2h0IGNoYW5nZSBkdXJpbmcgdGhlIGV4cGVyaW1lbnQNCiMgDQojIA0KIyAqIENvbHVtbnMgc3BlY2lmaWMgdG8gU3BhaW4NCiMgTWVDOiBNZXRoYW5lIGNvbmNlbnRyYXRpb24NCiMgZmFybQ0KIyByb2JvdA0KIyBOTEFDVEE6IExhY3RhdGlvbiBudW1iZXINCiMgRElNX2NhdGVnb3JpY2FsOiBTdGFnZSBvZiBsYWN0YXRpb24NCmlmKCFleGlzdHMoIm1ldGFkYXRhIikpew0KICBsb2FkKCIuL21ldGFkYXRhX3NpbS5SRGF0YSIpDQogIG1ldGFkYXRhIDwtIG1ldGFfc2ltDQp9DQoNCg0KIyBIT1NUIEdORU9UWVBFUw0KIyBGb3JtYXQ6IFR3byBzcGFjZS1kZWxpbWl0ZWQgY29sdW1ucy4gDQojIEZpcnN0IGNvbHVtbjogYW5pbWFsIElEDQojIFNlY29uZCBjb2x1bW46IGduZW90eXBlIGluIDAxMiBmb3JtYXQNCmlmKCFleGlzdHMoImdlbm90eXBlIikpew0KICBsb2FkKCIuL2dlbm90eXBlX3NpbS5SRGF0YSIpIA0KICBnZW5vdHlwZSA8LSBnZW5vX3NpbQ0KfQ0KDQoNCiMgTWF0cmljZXMgd2l0aCBtZXRhZ2Vub21lIGFic29sdXRlIGFidW5kYW5jZXMgb2YgS09zIHdpdGhvdXQgS09zIHByZXNlbnRlZCBpbiBCb3MgdGF1cnVzLiBPbmUgbWF0cml4IHBlciBwb3B1bGF0aW9uIChBdXN0cmFsaWEgYW5kIFNwYWluKQ0KIyMgQXVzdHJhbGlhDQppZighZXhpc3RzKCJhYnNvbHV0ZS5hYnVuZGFuY2UuQXVzdHJhbGlhIikpew0KICBsb2FkKCIuL2Fic29sdXRlX2FidW5kYW5jZV9BdXN0cmFsaWFfc2ltLlJEYXRhIikNCiAgYWEuYXVzIDwtIGFhQV9zaW0NCn0NCiMjIFNwYWluDQppZighZXhpc3RzKCJhYnNvbHV0ZS5hYnVuZGFuY2UuU3BhaW4iKSl7DQogIGxvYWQoIi4vYWJzb2x1dGVfYWJ1bmRhbmNlX1NwYWluX3NpbS5SRGF0YSIpDQogIGFhLnNwYSA8LSBhYVNfc2ltDQp9DQoNCg0KIyBGVU5DVElPTlMgQU5EIFBBQ0tBR0VTDQpzb3VyY2UoIi4vU3VwcGxlbWVudGFyeSBDb2RlIDEgLSBmdW5jdGlvbnMuUiIsIGVjaG89VFJVRSkNCmBgYA0KDQoNCiMgRGlmZmVyZW5jZXMgaW4gbWV0aGFuZSBlbWlzc2lvbnMgYWNyb3NzIGZhcm1zDQpgYGB7cn0NCmhhc19hc3JlbWwgPC0gcmVxdWlyZU5hbWVzcGFjZSgiYXNyZW1sIiwgcXVpZXRseSA9IFRSVUUpDQppZiAoIWhhc19hc3JlbWwpIHsNCiAgd2FybmluZygiVGhlIHBhY2thZ2UgJ2FzcmVtbCcgaXMgbm90IGluc3RhbGxlZC4gU29tZSBmdW5jdGlvbnMgbWF5IG5vdCB3b3JrIHByb3Blcmx5LiIpDQp9DQoNCiMgQXVzdHJhbGlhDQpkYXRhX3RtcCA9IG1ldGFkYXRhW21ldGFkYXRhJHBvcHVsYXRpb24gPT0gJ2F1c3RyYWxpYScsDQogICAgICAgICAgICAgICAgICAgIHdoaWNoKGNvbG5hbWVzKG1ldGFkYXRhKSAlaW4lIGMoJ01lUC54JyAsICdDb2hvcnQnICwgJ2RtaScgLCAnRElNJyAsICdtRUNNJyAsICdMV1RkZWx0YScpKV0NCmRhdGFfdG1wID0gZGF0YV90bXBbY29tcGxldGUuY2FzZXMoZGF0YV90bXApLF0NCmlmIChoYXNfYXNyZW1sKSB7DQogIGZpeGVkX2VmZmVjdF9tb2RlbF9hdXN0cmFsaWEgPC0NCiAgICBhc3JlbWw6OmFzcmVtbChNZVAueCB+IDEgKyBhcy5mYWN0b3IoQ29ob3J0KSArIGRtaSArIERJTSArIG1FQ00gKyBMV1RkZWx0YSwgZGF0YSA9IGRhdGFfdG1wKQ0KICBhc3JlbWw6OndhbGQuYXNyZW1sKGZpeGVkX2VmZmVjdF9tb2RlbF9hdXN0cmFsaWEpDQp9DQoNCiMgU3BhaW4NCmRhdGFfdG1wID0gbWV0YWRhdGFbbWV0YWRhdGEkcG9wdWxhdGlvbiA9PSAnc3BhaW4nLA0KICAgICAgICAgICAgICAgICAgICB3aGljaChjb2xuYW1lcyhtZXRhZGF0YSkgJWluJSBjKCdNZUMnICwgJ2xhY3RhdGlvbl9udW1iZXInICwgJ3N0YWdlX29mX2xhY3RhdGlvbicgLCAnZmFybScgLCAncm9ib3QnKSldDQpkYXRhX3RtcCRmYXJtIDwtIGFzLmZhY3RvcihkYXRhX3RtcCRmYXJtKQ0KZGF0YV90bXAkcm9ib3QgPC0gYXMuZmFjdG9yKGRhdGFfdG1wJHJvYm90KQ0KZGF0YV90bXAkc3RhZ2Vfb2ZfbGFjdGF0aW9uIDwtIGFzLmZhY3RvcihkYXRhX3RtcCRzdGFnZV9vZl9sYWN0YXRpb24pDQppZiAoaGFzX2FzcmVtbCkgew0KICBmaXhlZF9lZmZlY3RfbW9kZWxfc3BhaW4gPC0NCiAgICBhc3JlbWw6OmFzcmVtbChNZUMgfiAxICsgbGFjdGF0aW9uX251bWJlciArIHN0YWdlX29mX2xhY3RhdGlvbiArIGZhcm0gKyByb2JvdCwNCiAgICAgICAgICAgICAgICAgICBkYXRhID0gZGF0YV90bXApDQogIGFzcmVtbDo6d2FsZC5hc3JlbWwoZml4ZWRfZWZmZWN0X21vZGVsX3NwYWluKQ0KfQ0KDQojIC8vLy8vLy8vLy8vLy8vLy8vLy8vLy8vLy8vLy8NCiMgUGxvdHMgDQojIC8vLy8vLy8vLy8vLy8vLy8vLy8vLy8vLy8vLy8NCg0KIyBTdWJzZXQgcmVsZXZhbnQgY29sdW1ucw0KZGF0YV90bXAgPC0gbWV0YWRhdGEgJT4lIA0KICBzZWxlY3QocG9wdWxhdGlvbiwgTWVQLngsIE1lQywgZmFybSkNCg0KIyBMb25nIGZvcm1hdCBhbmQgcmVsYWJlbCB0cmFpdHMNCmRhdGFfbG9uZyA8LSBkYXRhX3RtcCAlPiUNCiAgcGl2b3RfbG9uZ2VyKGNvbHMgPSBjKE1lUC54LCBNZUMpLCBuYW1lc190byA9ICJ0cmFpdCIsIHZhbHVlc190byA9ICJ2YWx1ZSIpICU+JQ0KICBmaWx0ZXIoIWlzLm5hKHZhbHVlKSkgJT4lDQogIG11dGF0ZSgNCiAgICB0cmFpdF9sYWJlbCA9IGNhc2Vfd2hlbigNCiAgICAgIHRyYWl0ID09ICJNZVAueCIgfiAiTWV0aGFuZSBwcm9kdWN0aW9uIChnL2QpIGluIEF1c3RyYWxpYSIsDQogICAgICB0cmFpdCA9PSAiTWVDIiAgIH4gIk1ldGhhbmUgY29uY2VudHJhdGlvbiAocHBtKSBpbiBTcGFpbiINCiAgICApLA0KICAgIHRyYWl0X2xhYmVsID0gZmFjdG9yKHRyYWl0X2xhYmVsLCBsZXZlbHMgPSBjKA0KICAgICAgIk1ldGhhbmUgcHJvZHVjdGlvbiAoZy9kKSBpbiBBdXN0cmFsaWEiLA0KICAgICAgIk1ldGhhbmUgY29uY2VudHJhdGlvbiAocHBtKSBpbiBTcGFpbiINCiAgICApKQ0KICApDQoNCiMgQ3JlYXRlIHNlcGFyYXRlIHN1YnNldHMgZXhwbGljaXRseSBmb3IgcGxvdHMNCmRhdGFfYXVzIDwtIGRhdGFfbG9uZyAlPiUNCiAgZmlsdGVyKHBvcHVsYXRpb24gPT0gImF1c3RyYWxpYSIsIHRyYWl0ID09ICJNZVAueCIpICU+JQ0KICBtdXRhdGUoZmFybSA9IGZhY3RvcihmYXJtLCBsZXZlbHMgPSBzdHJfc29ydCh1bmlxdWUoZmFybSksIG51bWVyaWMgPSBUUlVFKSkpDQoNCmRhdGFfc3BhaW4gPC0gZGF0YV9sb25nICU+JQ0KICBmaWx0ZXIocG9wdWxhdGlvbiA9PSAic3BhaW4iLCB0cmFpdCA9PSAiTWVDIikgJT4lDQogIG11dGF0ZShmYXJtID0gZmFjdG9yKGZhcm0sIGxldmVscyA9IHN0cl9zb3J0KHVuaXF1ZShmYXJtKSwgbnVtZXJpYyA9IFRSVUUpKSkNCg0KIyBQbG90IE1ldGhhbmUgUHJvZHVjdGlvbiBpbiBBdXN0cmFsaWEgKE9ubHkgRWxsaW5iYW5rKQ0KcDEgPC0gZ2dwbG90KGRhdGFfYXVzLCBhZXMoeCA9IGZhcm0sIHkgPSB2YWx1ZSkpICsNCiAgZ2VvbV9ib3hwbG90KG91dGxpZXIuYWxwaGEgPSAwLjMsIGZpbGwgPSAiYmx1ZSIpICsNCiAgdGhlbWVfbWluaW1hbCgpICsNCiAgdGhlbWUoDQogICAgYXhpcy50ZXh0LnggPSBlbGVtZW50X3RleHQoYW5nbGUgPSA0NSwgaGp1c3QgPSAxKQ0KICApICsNCiAgbGFicygNCiAgICB5ID0gIk1ldGhhbmUgcHJvZHVjdGlvbiAoZy9kKSIsDQogICAgeCA9ICJGYXJtIiwNCiAgICB0aXRsZSA9ICJNZXRoYW5lIHByb2R1Y3Rpb24gKGcvZCkgaW4gQXVzdHJhbGlhIg0KICApDQoNCiMgUGxvdDogTWV0aGFuZSBDb25jZW50cmF0aW9uIGluIFNwYWluDQpwMiA8LSBnZ3Bsb3QoZGF0YV9zcGFpbiwgYWVzKHggPSBmYXJtLCB5ID0gdmFsdWUpKSArDQogIGdlb21fYm94cGxvdChvdXRsaWVyLmFscGhhID0gMC4zLCBmaWxsID0gInJlZCIpICsNCiAgdGhlbWVfbWluaW1hbCgpICsNCiAgdGhlbWUoDQogICAgYXhpcy50ZXh0LnggPSBlbGVtZW50X3RleHQoYW5nbGUgPSA0NSwgaGp1c3QgPSAxKQ0KICApICsNCiAgbGFicygNCiAgICB5ID0gIk1ldGhhbmUgY29uY2VudHJhdGlvbiAocHBtKSIsDQogICAgeCA9ICJGYXJtIiwNCiAgICB0aXRsZSA9ICJNZXRoYW5lIGNvbmNlbnRyYXRpb24gKHBwbSkgaW4gU3BhaW4iDQogICkNCg0KIyBDb21iaW5lIHBsb3RzDQpjb21iaW5lZF9wMV9wMiA8LSBwMSArIHAyICsgcGxvdF9sYXlvdXQobmNvbCA9IDIpDQoNCiMgUGxvdCBDb2VmZmljaWVudCBvZiBWYXJpYXRpb24gDQpjdl9kZiA8LSBiaW5kX3Jvd3MoZGF0YV9hdXMsIGRhdGFfc3BhaW4pICU+JQ0KICBncm91cF9ieShwb3B1bGF0aW9uLCBmYXJtLCB0cmFpdF9sYWJlbCkgJT4lDQogIHN1bW1hcmlzZSgNCiAgICBtZWFuID0gbWVhbih2YWx1ZSwgbmEucm0gPSBUUlVFKSwNCiAgICBzZCA9IHNkKHZhbHVlLCBuYS5ybSA9IFRSVUUpLA0KICAgIGN2ID0gc2QgLyBtZWFuLA0KICAgIC5ncm91cHMgPSAiZHJvcCINCiAgKSAlPiUNCiAgbXV0YXRlKA0KICAgIGZhcm1fcG9wID0gcGFzdGUoZmFybSwgcG9wdWxhdGlvbiwgc2VwID0gIl8iKSwNCiAgICBmYXJtX3BvcCA9IGZhY3RvcihmYXJtX3BvcCwgbGV2ZWxzID0gc3RyX3NvcnQodW5pcXVlKGZhcm1fcG9wKSwgbnVtZXJpYyA9IFRSVUUpKQ0KICApDQoNCnAzIDwtIGdncGxvdChjdl9kZiwgYWVzKHggPSBmYXJtX3BvcCwgeSA9IGN2LCBmaWxsID0gdHJhaXRfbGFiZWwpKSArDQogIGdlb21fY29sKCkgKw0KICBzY2FsZV9maWxsX21hbnVhbCh2YWx1ZXMgPSBjKA0KICAgICJNZXRoYW5lIHByb2R1Y3Rpb24gKGcvZCkgaW4gQXVzdHJhbGlhIiA9ICJibHVlIiwNCiAgICAiTWV0aGFuZSBjb25jZW50cmF0aW9uIChwcG0pIGluIFNwYWluIiA9ICJyZWQiDQogICkpICsNCiAgdGhlbWVfbWluaW1hbCgpICsNCiAgdGhlbWUoDQogICAgYXhpcy50ZXh0LnggPSBlbGVtZW50X3RleHQoYW5nbGUgPSA0NSwgaGp1c3QgPSAxKSwNCiAgICBsZWdlbmQucG9zaXRpb24gPSAibm9uZSINCiAgKSArDQogIGxhYnMoDQogICAgeSA9ICJDb2VmZmljaWVudCBvZiBWYXJpYXRpb24gKENWKSIsDQogICAgeCA9ICJGYXJtICsgUG9wdWxhdGlvbiIsDQogICAgdGl0bGUgPSAiQ1Ygb2YgbWV0aGFuZSB0cmFpdHMgYWNyb3NzIGZhcm1zIg0KICApDQoNCiMgQ29tYmluZSBhbGwgcGxvdHMNCmZpbmFsX3Bsb3QgPC0gY29tYmluZWRfcDFfcDIgLyBwMw0KZmluYWxfcGxvdCANCiANCmBgYA0KDQoNCiMgR2Vub21pYyByZWxhdGlvbnNoaXAgbWF0cml4IChHUk0pIGFuZCBpdHMgaW52ZXJzZQ0KYGBge3J9DQppZighZXhpc3RzKCJpbnZlcnNlR1JNIikpew0KICBncm1BbmRJbnYgPC0gZ2Vub3MyZ2ludihnZW5vdHlwZXMgPSBnZW5vdHlwZSwgbWV0aG9kID0gIllhbmciLCBtYWYgPSAwLjA1KQ0KICBncm0gPC0gZ3JtQW5kSW52JEdSTQ0KICBpbnZlcnNlR1JNIDwtIGdybUFuZEludiRnaW52DQp9DQoNCmBgYA0KDQoNCiMgTWljcm9iaWFsIHJlbGF0aW9uc2hpcCBtYXRyaXggKE1STSkgDQoNCk1ldGFnZW5vbWUgZGF0YSB0cmFuc2Zvcm1hdGlvbg0KYGBge3J9DQojIFByZXZhbGVuY2UgKFApIGZpbHRlci4NCnByZXYuYXVzIDwtIGFhLmF1c1thcHBseShhYS5hdXMsIDEsIGZ1bmN0aW9uKHgpIGlzX2luX21vc3Rfc2FtcGxlcyh4LCBwID0gMTAwKSksIF0NCnByZXYuc3BhIDwtIGFhLnNwYVthcHBseShhYS5zcGEsIDEsIGZ1bmN0aW9uKHgpIGlzX2luX21vc3Rfc2FtcGxlcyh4LCBwID0gMTAwKSksIF0NCg0KIyBSZW1vdmUgS09zIG5vIHByZXNlbnRlZCBpbiBhbGwgYW5pbWFscyBvZiBib3RoIHBvcHVsYXRpb25zDQpjb21tb25fS09zIDwtIHJvd25hbWVzKHByZXYuYXVzKVtyb3duYW1lcyhwcmV2LmF1cyklaW4lcm93bmFtZXMocHJldi5zcGEpXQ0KcHJldi5hdXMgPC0gcHJldi5hdXNbY29tbW9uX0tPcyxdDQpwcmV2LnNwYSA8LSBwcmV2LnNwYVtjb21tb25fS09zLF0NCg0KIyBSZWxhdGl2ZSBhYnVuZGFuY2UNCnJhLmF1cyA8LSBzd2VlcChwcmV2LmF1cywgMiwgY29sU3VtcyhwcmV2LmF1cyksIEZVTiA9ICIvIikgDQpyYS5zcGEgPC0gc3dlZXAocHJldi5zcGEsIDIsIGNvbFN1bXMocHJldi5zcGEpLCBGVU4gPSAiLyIpIA0KDQojIFRyYW5zcG9zZSBvZiBSQQ0KQi5hdXMgPC0gdChyYS5hdXMpDQpCLnNwYSA8LSB0KHJhLnNwYSkNCg0KIyBJTFIgdHJhbnNmb3JtYXRpb24NCmlmKGxvZ190cmFuc2Zvcm1hdGlvbiA9PSAnY2xyJyl7DQogIGx0LmF1cyA8LSBlYXN5Q09EQTo6Q0xSKEIuYXVzLCB3ZWlnaHQgPSBGQUxTRSkNCiAgbHQuYXVzIDwtIGx0LmF1cyRMUg0KICBsdC5zcGEgPC0gZWFzeUNPREE6OkNMUihCLnNwYSwgd2VpZ2h0ID0gRkFMU0UpDQogIGx0LnNwYSA8LSBsdC5zcGEkTFINCn0NCmlmKGxvZ190cmFuc2Zvcm1hdGlvbiA9PSAnYWxyJyl7DQogIGx0LmF1cyA8LSBlYXN5Q09EQTo6QUxSKEIuYXVzLCB3ZWlnaHQgPSBGQUxTRSkNCiAgbHQuYXVzIDwtIGx0LmF1cyRMUg0KICBsdC5zcGEgPC0gZWFzeUNPREE6OkFMUihCLnNwYSwgd2VpZ2h0ID0gRkFMU0UpDQogIGx0LnNwYSA8LSBsdC5zcGEkTFINCn0NCmlmKGxvZ190cmFuc2Zvcm1hdGlvbiA9PSAnaWxyJyl7DQogIGx0LmF1cyA8LSBhcy5tYXRyaXgoY29tcG9zaXRpb25zOjppbHIoQi5hdXMpKQ0KICBsdC5zcGEgPC0gYXMubWF0cml4KGNvbXBvc2l0aW9uczo6aWxyKEIuc3BhKSkNCn0NCg0KICANCiMgU2NhbGUgYW5kIGNlbnRlciBLT3MgYWNyb3NzIGFuaW1hbHMNCmx0LmF1cyA8LSBzY2FsZShsdC5hdXMpIA0KbHQuc3BhIDwtIHNjYWxlKGx0LnNwYSkgDQoNCmBgYA0KDQoNCkNvbXBhcmlzb24gb2YgS08gYWJ1bmRhbmNlcyBiZXR3ZWVuIGNvdW50cmllcw0KYGBge3J9DQojIENMUiB0cmFuc2Zvcm1hdGlvbg0KY2xyLmF1cyA8LSBlYXN5Q09EQTo6Q0xSKEIuYXVzLCB3ZWlnaHQgPSBGQUxTRSkNCmNsci5hdXMgPC0gY2xyLmF1cyRMUg0KY2xyLnNwYSA8LSBlYXN5Q09EQTo6Q0xSKEIuc3BhLCB3ZWlnaHQgPSBGQUxTRSkNCmNsci5zcGEgPC0gY2xyLnNwYSRMUg0KDQojIEVuc3VyZSBib3RoIG1hdHJpY2VzIGhhdmUgdGhlIHNhbWUgS09zIGFuZCBpbiB0aGUgc2FtZSBvcmRlcg0Ka29zIDwtIGludGVyc2VjdChjb2xuYW1lcyhjbHIuYXVzKSwgY29sbmFtZXMoY2xyLnNwYSkpDQpuX2tvcyA8LSBsZW5ndGgoa29zKQ0KDQojIyMjIyMjIyMjIyMjIyMjIyMjIyMjIyMjIyMjIyMjIyMjIyMjDQojIEVzdGltYXRlIHNpZ25pZmljYW5jZSBvZiBkaWZmZXJlbmNlcyBiZXR3ZWVuIGNvdW50cmllcyBpbiB0ZXJtcyBvZiBDTFItdHJhbnNmb3JtZWQgS09zDQojIyMjIyMjIyMjIyMjIyMjIyMjIyMjIyMjIyMjIyMjIyMjIyMjDQpyZXN1bHRzIDwtIGRhdGEuZnJhbWUoDQogIEtPICAgICAgICA9IGtvcywNCiAgcF92YWx1ZSAgID0gTkFfcmVhbF8sDQogIG1lYW5fQVVTICA9IE5BX3JlYWxfLA0KICBtZWFuX1NQQSAgPSBOQV9yZWFsXywNCiAgZGlmZiAgICAgID0gTkFfcmVhbF8sDQogIHN0cmluZ3NBc0ZhY3RvcnMgPSBGQUxTRQ0KKQ0KDQpmb3IgKGkgaW4gc2VxX2xlbihuX2tvcykpIHsNCiAga28gPC0ga29zW2ldDQoNCiAgIyBFeHRyYWN0IENMUi10cmFuc2Zvcm1lZCB2YWx1ZXMgYXMgdmVjdG9ycw0KICB4IDwtIGNsci5hdXNbLCBrb10gIA0KICB5IDwtIGNsci5zcGFbLCBrb10gIA0KDQogICMgTWVhbnMNCiAgcmVzdWx0cyRtZWFuX0FVU1tpXSA8LSBtZWFuKHgsIG5hLnJtID0gVFJVRSkNCiAgcmVzdWx0cyRtZWFuX1NQQVtpXSA8LSBtZWFuKHksIG5hLnJtID0gVFJVRSkNCiAgcmVzdWx0cyRkaWZmW2ldICAgICA8LSByZXN1bHRzJG1lYW5fQVVTW2ldIC0gcmVzdWx0cyRtZWFuX1NQQVtpXQ0KDQogICMgV2lsY294b24gcmFuay1zdW0gdGVzdA0KICB3dCA8LSB3aWxjb3gudGVzdCh4LCB5LCBleGFjdCA9IEZBTFNFKSANCiAgcmVzdWx0cyRwX3ZhbHVlW2ldIDwtIHd0JHAudmFsdWUNCn0NCg0KIyBGRFIgY29ycmVjdGlvbg0KcmVzdWx0cyRxX3ZhbHVlIDwtIHAuYWRqdXN0KHJlc3VsdHMkcF92YWx1ZSwgbWV0aG9kID0gImZkciIpDQoNCiMgT3JkZXIgYnkgcS12YWx1ZQ0KcmVzdWx0cyA8LSByZXN1bHRzW29yZGVyKHJlc3VsdHMkcV92YWx1ZSksIF0NCg0KIyBBQlNPTFVURSBBQlVOREFOQ0UgT0YgS09zIElOIEFVU1RSQUxJQQ0Kd3JpdGUudGFibGUocmVzdWx0cywgZmlsZSA9ICIuL0NMUi10cmFuc2Zvcm1lZF9LT3NfZGlmZmVyZW5jZXMudHh0Iiwgcm93Lm5hbWVzID0gRiwgY29sLm5hbWVzID0gVCwgcXVvdGUgPSBGLCBzZXAgPSAiXHQiKSANCg0KDQojIyMjIyMjIyMjIyMjIyMjIyMjIyMjIyMjIyMjIyMjIyMjIyMjDQojIENvcnJlbGF0aW9uIENMUi1hYnVuZGFuY2VzIGJldHdlZW4gY291bnRyaWVzDQojIyMjIyMjIyMjIyMjIyMjIyMjIyMjIyMjIyMjIyMjIyMjIyMjDQptZWFuX2F1cyA8LSBjb2xNZWFucyhjbHIuYXVzKQ0KbWVhbl9zcGEgPC0gY29sTWVhbnMoY2xyLnNwYSkNCg0KcGxvdF9kZiA8LSBkYXRhLmZyYW1lKA0KICBLTyA9IG5hbWVzKG1lYW5fYXVzKSwNCiAgQVVTID0gbWVhbl9hdXMsDQogIFNQQSA9IG1lYW5fc3BhDQopDQoNCmZpdCA8LSBsbShTUEEgfiBBVVMsIGRhdGEgPSBwbG90X2RmKQ0KUjIgPC0gcm91bmQoc3VtbWFyeShmaXQpJHIuc3F1YXJlZCwyKQ0KDQpsaWJyYXJ5KGdncGxvdDIpDQoNCmdncGxvdChwbG90X2RmLCBhZXMoeCA9IEFVUywgeSA9IFNQQSkpICsNCiAgZ2VvbV9wb2ludChhbHBoYSA9IDAuNSwgc2l6ZSA9IDIsIGNvbG9yID0gInN0ZWVsYmx1ZSIpICsNCiAgZ2VvbV9hYmxpbmUoc2xvcGUgPSAxLCBpbnRlcmNlcHQgPSAwLCBsaW5ldHlwZSA9ICJkYXNoZWQiLCBjb2xvciA9ICJncmF5NTAiKSArDQogIGdlb21fc21vb3RoKG1ldGhvZCA9ICJsbSIsIHNlID0gRkFMU0UsIGNvbG9yID0gImZpcmVicmljayIsIGxpbmV3aWR0aCA9IDEpICsNCiAgYW5ub3RhdGUoDQogICAgInRleHQiLA0KICAgIHggPSBtaW4ocGxvdF9kZiRBVVMpLA0KICAgIHkgPSBtYXgocGxvdF9kZiRTUEEpLA0KICAgIGhqdXN0ID0gMCwgdmp1c3QgPSAxLA0KICAgIGxhYmVsID0gcGFzdGUwKCJSwrIgPSAiLCByb3VuZChSMiwgMykpLA0KICAgIHNpemUgPSA1LCBjb2xvciA9ICJmaXJlYnJpY2siDQogICkgKw0KICBsYWJzKA0KICAgIHggPSAiTWVhbiBDTFIgYWJ1bmRhbmNlIChBdXN0cmFsaWEpIiwNCiAgICB5ID0gIk1lYW4gQ0xSIGFidW5kYW5jZSAoU3BhaW4pIiwNCiAgICB0aXRsZSA9ICJDb21wYXJpc29uIG9mIEtPIGFidW5kYW5jZXMgYmV0d2VlbiBjb3VudHJpZXMiDQogICkgKw0KICB0aGVtZV9idyhiYXNlX3NpemUgPSAxNCkNCg0KYGBgDQoNCg0KVXNlIHRoZSBzZWxlY3RlZCBwb3B1bGF0aW9uDQpgYGB7cn0NCmlmKHBvcHVsYXRpb24gPT0gIkF1c3RyYWxpYSIpeyANCiBsdCA8LSBsdC5hdXMNCiBhYS5wb3AgPC0gYWEuYXVzDQogQiA9IEIuYXVzDQp9IGVsc2UgaWYocG9wdWxhdGlvbiA9PSAiU3BhaW4iKXsNCiBsdCA8LSBsdC5zcGENCiBhYS5wb3AgPC0gYWEuc3BhDQogQiA9IEIuc3BhDQp9DQpgYGANCg0KDQoNCkNyZWF0ZSB0aGUgbWljcm9iaWFsIHJlbGF0aW9uc2hpcCBtYXRyaXggKE1STSkgYW5kIGl0cyBpbnZlcnNlDQpgYGB7cn0NCiMgQ3Jvc3MtcHJvZHVjdCBtYXRyaXgNCmNycHIgPC0gbHQgJSolIHQobHQpDQoNCm1ybSA8LSAoMSAvIG5yb3coYWEucG9wKSkgKiBjcnByDQoNCiMgQWRkIGEgc21hbGwgdmFsdWUgdG8gdGhlIG1haW4gZGlhZ29uYWwgYmVjYXVzZSBpdCdzIHNpbmd1bGFyDQptcm0gPC0gbXJtICsgZGlhZygxZS04LCBucm93KG1ybSkpDQpyb3duYW1lcyhtcm0pIDwtIHJvd25hbWVzKGNycHIpDQpjb2xuYW1lcyhtcm0pIDwtIGNvbG5hbWVzKGNycHIpDQoNCiMgSW52ZXJzZSBvZiBNUk0NCmludmVyc2VNUk0gPC0gbXJtSW52RnVuYyhtcm0pDQoNCiMgQWRkIGEgaWRlbnRpZmllciBvZiB0aGUgcnVtZW4gbWV0YWdlbm9tZXMgKE1FSUQpIHRvIHRoZSBtZXRhZGF0YQ0KQl9mb3JfYWRkTUVJRDJwaGVuIDwtIGRhdGEudGFibGUocm93Lm5hbWVzKEIpLCBCKQ0Kcm93Lm5hbWVzKEJfZm9yX2FkZE1FSUQycGhlbikgPC0gYygxOm5yb3coQl9mb3JfYWRkTUVJRDJwaGVuKSkNCm1ldGFnZW5vbWVfc2FtcGxlX2luX3BoZW4gPC0gd2hpY2goY29sbmFtZXMobWV0YWRhdGEpID09ICJBbmltYWxfSUQiKQ0KbmV3X2NvbF9vcmRlciA8LSBjKG1ldGFnZW5vbWVfc2FtcGxlX2luX3BoZW4sIHNldGRpZmYoMTpuY29sKG1ldGFkYXRhKSwgbWV0YWdlbm9tZV9zYW1wbGVfaW5fcGhlbikpDQptZXRhZGF0YSA8LSBtZXRhZGF0YVssIG5ld19jb2xfb3JkZXJdDQptZXRhZGF0YSA8LSBhZGRNRUlEMnBoZW4obWV0YWdlbm9tZXMgPSBCX2Zvcl9hZGRNRUlEMnBoZW4sIHBoZW5vdHlwZV9hbmRfbWV0YWRhdGEgPSBtZXRhZGF0YSkgDQpgYGANCg0KDQojIEludGVyYWN0aW9uIGJldHdlZW4gR1JNIGFuZCBNUk0gDQpgYGB7cn0NCmhhZEdybU1ybSA8LSBhZGFtYXJkR3JtTXJtKEdSTSA9IGdybSwgTVJNID0gbXJtLCBtZXRhZGF0YSA9IG1ldGFkYXRhLCBnZW5vdHlwZSA9IGdlbm90eXBlKVtbMV1dDQptZXRhZGF0YSA8LSBhZGFtYXJkR3JtTXJtKEdSTSA9IGdybSwgTVJNID0gbXJtLCBtZXRhZGF0YSA9IG1ldGFkYXRhLCBnZW5vdHlwZSA9IGdlbm90eXBlKVtbMl1dDQppbnZlcnNlSGFkR3JtTXJtIDwtIG1ybUludkZ1bmMoaGFkR3JtTXJtKQ0KYGBgDQoNCg0KIyBSdW4gcHJlZGljdGlvbiBtb2RlbHMNCmBgYHtyfQ0KbWV0YWRhdGEkR0VJRCA8LSBhcy5mYWN0b3IobWV0YWRhdGEkR0VJRCkNCm1ldGFkYXRhJE1FSUQgPC0gYXMuZmFjdG9yKG1ldGFkYXRhJE1FSUQpDQptZXRhZGF0YSRoYWRHcm1Ncm0gPC0gYXMuZmFjdG9yKG1ldGFkYXRhJGhhZEdybU1ybSkNCm1ldGFkYXRhJGZhcm0gPC0gYXMuZmFjdG9yKG1ldGFkYXRhJGZhcm0pDQptZXRhZGF0YSRyb2JvdCA8LSBhcy5mYWN0b3IobWV0YWRhdGEkcm9ib3QpDQptZXRhZGF0YSRzdGFnZV9vZl9sYWN0YXRpb24gPC0gYXMuZmFjdG9yKG1ldGFkYXRhJHN0YWdlX29mX2xhY3RhdGlvbikNCmlmKHBvcHVsYXRpb24gPT0gJ0F1c3RyYWxpYScpe0VNRSA9ICdNZVAueCd9DQppZihwb3B1bGF0aW9uID09ICdTcGFpbicpe0VNRSA9ICdNZUMnfQ0KaWYgKGhhc19hc3JlbWwpIHsNCiAgYXNyZW1sTW9kZWxzIDwtIGxpc3QoKQ0KICBmb3IgKG1vZGVsIGluIGMoIkhpQkxVUCIsICJIQkxVUCIsICJNQkxVUCIsICJHQkxVUCIpKSB7DQogICAgcHJpbnQocGFzdGUwKA0KICAgICAgIlN0YXJ0IHBvcHVsYXRpb24gIiwNCiAgICAgIHBvcHVsYXRpb24sDQogICAgICAiLCB0cmFpdDogIiwNCiAgICAgIEVNRSwNCiAgICAgICIsIGFuZCBtb2RlbCAiLA0KICAgICAgbW9kZWwNCiAgICApKQ0KICAgIGFzcmVtbE1vZGVsc1tbcG9wdWxhdGlvbl1dW1ttb2RlbF1dIDwtDQogICAgICBkby5jYWxsKGFzcmVtbCwNCiAgICAgICAgICAgICAgYXNyZW1sLmFyZ3MoDQogICAgICAgICAgICAgICAgcG9wdWxhdGlvbiA9IHBvcHVsYXRpb24sDQogICAgICAgICAgICAgICAgeSA9IEVNRSwNCiAgICAgICAgICAgICAgICBtb2RlbCA9IG1vZGVsDQogICAgICAgICAgICAgICkpDQogIH0NCn0gZWxzZXsNCiAgbWVzc2FnZSgiU2tpcHBpbmcgQVNSZW1sIG1vZGVscyBiZWNhdXNlICdhc3JlbWwnIGlzIG5vdCBpbnN0YWxsZWQuIikNCn0NCmBgYA0KDQoNCiMgRXN0aW1hdGlvbiBvZiB2YXJpYW5jZSBjb21wb25lbnRzDQpgYGB7cn0NCmlmIChoYXNfYXNyZW1sKSB7DQogIGlmIChwb3B1bGF0aW9uID09ICJBdXN0cmFsaWEiKSB7DQogICAgdmFyY29tcF9BdXN0cmFsaWEgPC0gZGF0YS5mcmFtZShtYXRyaXgoZGF0YSA8LSBOQSwgbnJvdyA9IDQsIG5jb2wgPSA0KSkNCiAgICByb3duYW1lcyh2YXJjb21wX0F1c3RyYWxpYSkgPC0gYygiSGlCTFVQIiwgIkhCTFVQIiwgIk1CTFVQIiwgIkdCTFVQIikNCiAgICBjb2xuYW1lcyh2YXJjb21wX0F1c3RyYWxpYSkgPC0gYygiaDIiLCAibTIiLCAiaTIiLCAiaG8yIikNCiAgICANCiAgICAjIEhpQkxVUCBBdXN0cmFsaWENCiAgICBzdW1tYXJ5KGFzcmVtbE1vZGVscyRBdXN0cmFsaWEkSGlCTFVQKSR2YXJjb21wICMgdmFyaWFuY2UgY29tcG9uZW50cyBvZiBIaUJMVVAgQXVzdHJhbGlhDQogICAgdmFyY29tcF9BdXN0cmFsaWFbIkhpQkxVUCIsICJtMiJdIDwtDQogICAgICBwYXN0ZVN0YXRzQW5kU0VfcGluKHJvdW5kKA0KICAgICAgICBuYWRpdjo6cGluKGFzcmVtbE1vZGVscyRBdXN0cmFsaWEkSGlCTFVQLCBtMiB+IFYxIC8gKFYxICsgVjIgKyBWMyArIFY0KSksDQogICAgICAgIGRpZ2l0cyA9IDINCiAgICAgICkpDQogICAgdmFyY29tcF9BdXN0cmFsaWFbIkhpQkxVUCIsICJpMiJdIDwtDQogICAgICBwYXN0ZVN0YXRzQW5kU0VfcGluKHJvdW5kKA0KICAgICAgICBuYWRpdjo6cGluKGFzcmVtbE1vZGVscyRBdXN0cmFsaWEkSGlCTFVQLCBpMiB+IFYyIC8gKFYxICsgVjIgKyBWMyArIFY0KSksDQogICAgICAgIGRpZ2l0cyA9IDINCiAgICAgICkpDQogICAgdmFyY29tcF9BdXN0cmFsaWFbIkhpQkxVUCIsICJoMiJdIDwtDQogICAgICBwYXN0ZVN0YXRzQW5kU0VfcGluKHJvdW5kKA0KICAgICAgICBuYWRpdjo6cGluKGFzcmVtbE1vZGVscyRBdXN0cmFsaWEkSGlCTFVQLCBoMiB+IFYzIC8gKFYxICsgVjIgKyBWMyArIFY0KSksDQogICAgICAgIGRpZ2l0cyA9IDINCiAgICAgICkpDQogICAgdmFyY29tcF9BdXN0cmFsaWFbIkhpQkxVUCIsICJobzIiXSA8LQ0KICAgICAgcGFzdGVTdGF0c0FuZFNFX3Bpbihyb3VuZCgNCiAgICAgICAgbmFkaXY6OnBpbigNCiAgICAgICAgICBhc3JlbWxNb2RlbHMkQXVzdHJhbGlhJEhpQkxVUCwNCiAgICAgICAgICBobzIgfiAoVjEgKyBWMiArIFYzKSAvIChWMSArIFYyICsgVjMgKyBWNCkNCiAgICAgICAgKSwNCiAgICAgICAgZGlnaXRzID0gMg0KICAgICAgKSkNCiAgICANCiAgICAjIEhCTFVQIEF1c3RyYWxpYQ0KICAgIHN1bW1hcnkoYXNyZW1sTW9kZWxzJEF1c3RyYWxpYSRIQkxVUCkkdmFyY29tcCAjIHZhcmlhbmNlIGNvbXBvbmVudHMgb2YgSEJMVVAgQXVzdHJhbGlhDQogICAgdmFyY29tcF9BdXN0cmFsaWFbIkhCTFVQIiwgIm0yIl0gPC0NCiAgICAgIHBhc3RlU3RhdHNBbmRTRV9waW4ocm91bmQoDQogICAgICAgIG5hZGl2OjpwaW4oYXNyZW1sTW9kZWxzJEF1c3RyYWxpYSRIQkxVUCwgbTIgfiBWMSAvIChWMSArIFYyICsgVjMpKSwNCiAgICAgICAgZGlnaXRzID0gMg0KICAgICAgKSkNCiAgICB2YXJjb21wX0F1c3RyYWxpYVsiSEJMVVAiLCAiaDIiXSA8LQ0KICAgICAgcGFzdGVTdGF0c0FuZFNFX3Bpbihyb3VuZCgNCiAgICAgICAgbmFkaXY6OnBpbihhc3JlbWxNb2RlbHMkQXVzdHJhbGlhJEhCTFVQLCBoMiB+IFYyIC8gKFYxICsgVjIgKyBWMykpLA0KICAgICAgICBkaWdpdHMgPSAyDQogICAgICApKQ0KICAgIHZhcmNvbXBfQXVzdHJhbGlhWyJIQkxVUCIsICJobzIiXSA8LQ0KICAgICAgcGFzdGVTdGF0c0FuZFNFX3Bpbihyb3VuZCgNCiAgICAgICAgbmFkaXY6OnBpbihhc3JlbWxNb2RlbHMkQXVzdHJhbGlhJEhCTFVQLCBobzIgfiAoVjEgKyBWMikgLyAoVjEgKyBWMiArIFYzKSksDQogICAgICAgIGRpZ2l0cyA9IDINCiAgICAgICkpDQogICAgDQogICAgIyBNQkxVUCBBdXN0cmFsaWENCiAgICBzdW1tYXJ5KGFzcmVtbE1vZGVscyRBdXN0cmFsaWEkTUJMVVApJHZhcmNvbXAgIyB2YXJpYW5jZSBjb21wb25lbnRzIG9mIE1CTFVQIEF1c3RyYWxpYQ0KICAgIHZhcmNvbXBfQXVzdHJhbGlhWyJNQkxVUCIsICJtMiJdIDwtDQogICAgICBwYXN0ZVN0YXRzQW5kU0VfcGluKHJvdW5kKA0KICAgICAgICBuYWRpdjo6cGluKGFzcmVtbE1vZGVscyRBdXN0cmFsaWEkTUJMVVAsIG0yIH4gVjEgLyAoVjEgKyBWMikpLA0KICAgICAgICBkaWdpdHMgPSAyDQogICAgICApKQ0KICAgIA0KICAgICMgR0JMVVAgQXVzdHJhbGlhDQogICAgc3VtbWFyeShhc3JlbWxNb2RlbHMkQXVzdHJhbGlhJEdCTFVQKSR2YXJjb21wICMgdmFyaWFuY2UgY29tcG9uZW50cyBvZiBHQkxVUCBBdXN0cmFsaWENCiAgICB2YXJjb21wX0F1c3RyYWxpYVsiR0JMVVAiLCAiaDIiXSA8LQ0KICAgICAgcGFzdGVTdGF0c0FuZFNFX3Bpbihyb3VuZCgNCiAgICAgICAgbmFkaXY6OnBpbihhc3JlbWxNb2RlbHMkQXVzdHJhbGlhJEdCTFVQLCBoMiB+IFYxIC8gKFYxICsgVjIpKSwNCiAgICAgICAgZGlnaXRzID0gMg0KICAgICAgKSkNCiAgICANCiAgICAjIFByaW50IHZhcmNvbXBzIGluIEF1c3RyYWxpYQ0KICAgIHZhcmNvbXBfQXVzdHJhbGlhDQogICAgDQogIH0gZWxzZSBpZiAocG9wdWxhdGlvbiA9PSAiU3BhaW4iKSB7DQogICAgdmFyY29tcF9TcGFpbiA8LSBkYXRhLmZyYW1lKG1hdHJpeChkYXRhIDwtIE5BLCBucm93ID0gNCwgbmNvbCA9IDQpKQ0KICAgIHJvd25hbWVzKHZhcmNvbXBfU3BhaW4pIDwtIGMoIkhpQkxVUCIsICJIQkxVUCIsICJNQkxVUCIsICJHQkxVUCIpDQogICAgY29sbmFtZXModmFyY29tcF9TcGFpbikgPC0gYygiaDIiLCAibTIiLCAiaTIiLCAiaG8yIikNCiAgICANCiAgICAjIEhpQkxVUCBTcGFpbg0KICAgIHN1bW1hcnkoYXNyZW1sTW9kZWxzJFNwYWluJEhpQkxVUCkkdmFyY29tcCAjIHZhcmlhbmNlIGNvbXBvbmVudHMgb2YgSGlCTFVQIFNwYWluDQogICAgdmFyY29tcF9TcGFpblsiSGlCTFVQIiwgIm0yIl0gPC0NCiAgICAgIHBhc3RlU3RhdHNBbmRTRV9waW4ocm91bmQobmFkaXY6OnBpbigNCiAgICAgICAgYXNyZW1sTW9kZWxzJFNwYWluJEhpQkxVUCwgbTIgfiBWMiAvIChWMSArIFYyICsgVjMgKyBWNCArIFY1KQ0KICAgICAgKSwgZGlnaXRzID0gMikpDQogICAgdmFyY29tcF9TcGFpblsiSGlCTFVQIiwgImkyIl0gPC0NCiAgICAgIHBhc3RlU3RhdHNBbmRTRV9waW4ocm91bmQobmFkaXY6OnBpbigNCiAgICAgICAgYXNyZW1sTW9kZWxzJFNwYWluJEhpQkxVUCwgaTIgfiBWMyAvIChWMSArIFYyICsgVjMgKyBWNCArIFY1KQ0KICAgICAgKSwgZGlnaXRzID0gMikpDQogICAgdmFyY29tcF9TcGFpblsiSGlCTFVQIiwgImgyIl0gPC0NCiAgICAgIHBhc3RlU3RhdHNBbmRTRV9waW4ocm91bmQobmFkaXY6OnBpbigNCiAgICAgICAgYXNyZW1sTW9kZWxzJFNwYWluJEhpQkxVUCwgaDIgfiBWNCAvIChWMSArIFYyICsgVjMgKyBWNCArIFY1KQ0KICAgICAgKSwgZGlnaXRzID0gMikpDQogICAgdmFyY29tcF9TcGFpblsiSGlCTFVQIiwgImhvMiJdIDwtDQogICAgICBwYXN0ZVN0YXRzQW5kU0VfcGluKHJvdW5kKG5hZGl2OjpwaW4oDQogICAgICAgIGFzcmVtbE1vZGVscyRTcGFpbiRIaUJMVVAsDQogICAgICAgIGhvMiB+IChWMiArIFYzICsgVjQpIC8gKFYxICsgVjIgKyBWMyArIFY0ICsgVjUpDQogICAgICApLCBkaWdpdHMgPSAyKSkNCiAgICANCiAgICAjIEhCTFVQIFNwYWluDQogICAgc3VtbWFyeShhc3JlbWxNb2RlbHMkU3BhaW4kSEJMVVApJHZhcmNvbXAgIyB2YXJpYW5jZSBjb21wb25lbnRzIG9mIEhCTFVQIFNwYWluDQogICAgdmFyY29tcF9TcGFpblsiSEJMVVAiLCAibTIiXSA8LQ0KICAgICAgcGFzdGVTdGF0c0FuZFNFX3Bpbihyb3VuZChuYWRpdjo6cGluKA0KICAgICAgICBhc3JlbWxNb2RlbHMkU3BhaW4kSEJMVVAsIG0yIH4gVjIgLyAoVjEgKyBWMiArIFYzICsgVjQpDQogICAgICApLCBkaWdpdHMgPSAyKSkNCiAgICB2YXJjb21wX1NwYWluWyJIQkxVUCIsICJoMiJdIDwtDQogICAgICBwYXN0ZVN0YXRzQW5kU0VfcGluKHJvdW5kKG5hZGl2OjpwaW4oDQogICAgICAgIGFzcmVtbE1vZGVscyRTcGFpbiRIQkxVUCwgaDIgfiBWMyAvIChWMSArIFYyICsgVjMgKyBWNCkNCiAgICAgICksIGRpZ2l0cyA9IDIpKQ0KICAgIHZhcmNvbXBfU3BhaW5bIkhCTFVQIiwgImhvMiJdIDwtDQogICAgICBwYXN0ZVN0YXRzQW5kU0VfcGluKHJvdW5kKG5hZGl2OjpwaW4oDQogICAgICAgIGFzcmVtbE1vZGVscyRTcGFpbiRIQkxVUCwgaG8yIH4gKFYyICsgVjMpIC8gKFYxICsgVjIgKyBWMyArIFY0KQ0KICAgICAgKSwgZGlnaXRzID0gMikpDQogICAgDQogICAgIyBNQkxVUCBTcGFpbg0KICAgIHN1bW1hcnkoYXNyZW1sTW9kZWxzJFNwYWluJE1CTFVQKSR2YXJjb21wICMgdmFyaWFuY2UgY29tcG9uZW50cyBvZiBNQkxVUCBTcGFpbg0KICAgIHZhcmNvbXBfU3BhaW5bIk1CTFVQIiwgIm0yIl0gPC0NCiAgICAgIHBhc3RlU3RhdHNBbmRTRV9waW4ocm91bmQobmFkaXY6OnBpbigNCiAgICAgICAgYXNyZW1sTW9kZWxzJFNwYWluJE1CTFVQLCBtMiB+IFYyIC8gKFYxICsgVjIgKyBWMykNCiAgICAgICksIGRpZ2l0cyA9IDIpKQ0KICAgIA0KICAgICMgR0JMVVAgU3BhaW4NCiAgICBzdW1tYXJ5KGFzcmVtbE1vZGVscyRTcGFpbiRHQkxVUCkkdmFyY29tcCAjIHZhcmlhbmNlIGNvbXBvbmVudHMgb2YgR0JMVVAgU3BhaW4NCiAgICB2YXJjb21wX1NwYWluWyJHQkxVUCIsICJoMiJdIDwtDQogICAgICBwYXN0ZVN0YXRzQW5kU0VfcGluKHJvdW5kKG5hZGl2OjpwaW4oDQogICAgICAgIGFzcmVtbE1vZGVscyRTcGFpbiRHQkxVUCwgaDIgfiBWMiAvIChWMSArIFYyICsgVjMpDQogICAgICApLCBkaWdpdHMgPSAyKSkNCiAgICANCiAgICAjIFByaW50IHZhcmNvbXBzIGluIFNwYWluDQogICAgdmFyY29tcF9TcGFpbg0KICB9DQp9IGVsc2V7DQogIG1lc3NhZ2UoIlNraXBwaW5nIHZhcmlhbmNlIGNvbXBvbmVudHMgZXN0aW1hdGlvbiBiZWNhdXNlICdhc3JlbWwnIGlzIG5vdCBpbnN0YWxsZWQuIikNCn0NCmBgYA0KDQoNCiMgRXN0aW1hdGlvbiBvZiBwcmVkaWN0aW9uIGFjY3VyYWN5IGFzIGEgMTAtcmVwZWF0ZWQgNS1mb2xkIGNyb3NzLXZhbGlkYXRpb24gDQpgYGB7cn0NCmlmIChoYXNfYXNyZW1sKSB7DQogIGlmICghZXhpc3RzKCJjb3JzVmFsIikpIHsNCiAgICBjb3JzVmFsIDwtIGxpc3QoKQ0KICAgIGZvciAobW9kZWwgaW4gYygiSGlCTFVQIiwgIkhCTFVQIiwgIk1CTFVQIiwgIkdCTFVQIikpIHsNCiAgICAgIGZvciAocmVwZXRpdGlvbiBpbiAxOjEwKSB7DQogICAgICAgIHByaW50KA0KICAgICAgICAgIHBhc3RlMCgNCiAgICAgICAgICAgICJTdGFydCBwb3B1bGF0aW9uICIsDQogICAgICAgICAgICBwb3B1bGF0aW9uLA0KICAgICAgICAgICAgIjsgbW9kZWwgIiwNCiAgICAgICAgICAgIG1vZGVsLA0KICAgICAgICAgICAgIjsgcmVwZXRpdGlvbiAiLA0KICAgICAgICAgICAgcmVwZXRpdGlvbiwNCiAgICAgICAgICAgICIgb2YgMTAiDQogICAgICAgICAgKQ0KICAgICAgICApDQogICAgICAgIGNvcnNWYWxbW3BvcHVsYXRpb25dXVtbbW9kZWxdXVtbcmVwZXRpdGlvbl1dIDwtIHh2YWxSYW5kb21Bc3JlbWwudjAyKHBvcHVsYXRpb24gPSBwb3B1bGF0aW9uLCBtb2RlbCA9IG1vZGVsKQ0KICAgICAgfQ0KICAgIH0NCiAgICANCiAgICAjIEF2ZXJhZ2UgYmV0d2VlbiByZXBldGl0aW9ucw0KICAgIG0gPC0gYygpDQogICAgc2QgPC0gYygpDQogICAgYWNjdXJhY3kgPC0gZGF0YS5mcmFtZShtYXRyaXgoDQogICAgICBkYXRhID0gTkEsDQogICAgICBucm93ID0gOCwNCiAgICAgIG5jb2wgPSA0DQogICAgKSkNCiAgICBjb2xuYW1lcyhhY2N1cmFjeSkgPC0gYygicG9wdWxhdGlvbiIsICJtb2RlbCIsICJtZWFuIiwgInNkIikNCiAgICBhY2N1cmFjeSRwb3B1bGF0aW9uIDwtIHJlcChjKCJBdXN0cmFsaWEiLCAiU3BhaW4iKSwgZWFjaCA9IDQpDQogICAgYWNjdXJhY3kkbW9kZWwgPC0gcmVwKGMoIkhpQkxVUCIsICJIQkxVUCIsICJNQkxVUCIsICJHQkxVUCIpLCAyKQ0KICAgIGZvciAobW9kZWwgaW4gYygiSGlCTFVQIiwgIkhCTFVQIiwgIk1CTFVQIiwgIkdCTFVQIikpIHsNCiAgICAgIGZvciAociBpbiAxOjEwKSB7DQogICAgICAgIG1bcl0gPC0gc3BsaXRTdGF0c0FuZERldmlhdGlvbihjb3JzVmFsW1twb3B1bGF0aW9uXV1bW21vZGVsXV1bW3JdXSlbWzFdXQ0KICAgICAgICBzZFtyXSA8LSBzcGxpdFN0YXRzQW5kRGV2aWF0aW9uKGNvcnNWYWxbW3BvcHVsYXRpb25dXVtbbW9kZWxdXVtbcl1dKVtbMl1dDQogICAgICB9DQogICAgICBhY2N1cmFjeVthY2N1cmFjeSRwb3B1bGF0aW9uID09IHBvcHVsYXRpb24gJg0KICAgICAgICAgICAgICAgICBhY2N1cmFjeSRtb2RlbCA9PSBtb2RlbCwgIm1lYW4iXSA8LQ0KICAgICAgICByb3VuZChtZWFuKHVubGlzdChtKSksIDIpDQogICAgICBhY2N1cmFjeVthY2N1cmFjeSRwb3B1bGF0aW9uID09IHBvcHVsYXRpb24gJg0KICAgICAgICAgICAgICAgICBhY2N1cmFjeSRtb2RlbCA9PSBtb2RlbCwgInNkIl0gPC0NCiAgICAgICAgcm91bmQobWVhbih1bmxpc3Qoc2QpKSwgMikNCiAgICB9DQogICAgDQogICAgcHJpbnQoYWNjdXJhY3kpDQogIH0gZWxzZSB7DQogICAgcHJpbnQoDQogICAgICBwYXN0ZSgNCiAgICAgICAgIkNyb3NzLXZhbGlkYXRpb24gaGFzIG5vdCBiZWVuIGV4ZWN1dGVkIHRvIGF2b2lkIG92ZXJ3cml0aW5nIGEgcHJldmlvdXMgcmVzdWx0LCB3aGljaCBsaWtlbHkgcmVxdWlyZWQgYSBzaWduaWZpY2FudCBhbW91bnQgb2YgdGltZSB0byBjb21wbGV0ZS4gSWYgeW91IHdpc2ggdG8gb3ZlcndyaXRlIGEgcHJldmlvdXMgY3Jvc3MtdmFsaWRhdGlvbiByZXN1bHQsIHBsZWFzZSByZW1vdmUgaXQgdXNpbmcgcm0oY29yc1ZhbCkgYW5kIGF0dGVtcHQgdGhpcyBzZWN0aW9uIGFnYWluLiINCiAgICAgICkNCiAgICApDQogIH0NCn0gZWxzZXsNCiAgICBtZXNzYWdlKCJTa2lwcGluZyBwcmVkaWN0aW9uIGFjY3VyYWN5IGVzdGltYXRpb24gYmVjYXVzZSAnYXNyZW1sJyBpcyBub3QgaW5zdGFsbGVkLiIpDQp9DQpgYGANCg0KDQojIEVzdGltYXRpb24gb2YgUmVmZXJlbmNlIFBvcHVsYXRpb24gU2l6ZQ0KYGBge3J9DQojIEVzdGltYXRpb24gb2YgcmVmZXJlbmNlIHBvcHVsYXRpb24gZm9yIGJvdGggcG9wdWxhdGlvbnMNCnJFSFZfQXVzdHJhbGlhIDwtIGRhdGEuZnJhbWUoTnAgPSBpbnRlZ2VyKCksIEFjY3VyYWN5ID0gbnVtZXJpYygpLCBTRSA9IG51bWVyaWMoKSkNCnJFSFZfU3BhaW4gPC0gZGF0YS5mcmFtZShOcCA9IGludGVnZXIoKSwgQWNjdXJhY3kgPSBudW1lcmljKCksIFNFID0gbnVtZXJpYygpKQ0KDQojIExvb3AgdGhyb3VnaCByZWZlcmVuY2UgcG9wdWxhdGlvbiBzaXplcw0KZm9yIChOcCBpbiAxOjEwMDAwKSB7DQogIHJlc3VsdF9BdXN0cmFsaWEgPC0gdGhlb3JldGljYWxIb2xvYmlvblByZWRpY2l0b25BY2N1cmFjeTIoUCA9IDEwMzIsIGhvMiA9IDAuNTksIE5wID0gTnApDQogIHJFSFZfQXVzdHJhbGlhIDwtIHJiaW5kKHJFSFZfQXVzdHJhbGlhLCBkYXRhLmZyYW1lKE5wID0gTnAsIEFjY3VyYWN5ID0gcmVzdWx0X0F1c3RyYWxpYSRBY2N1cmFjeSwgU0UgPSByZXN1bHRfQXVzdHJhbGlhJFNFKSkNCn0NCmZvciAoTnAgaW4gMToxMDAwMCkgew0KICByZXN1bHRfU3BhaW4gPC0gdGhlb3JldGljYWxIb2xvYmlvblByZWRpY2l0b25BY2N1cmFjeTIoUCA9IDEwMzIsIGhvMiA9IDAuMzQsIE5wID0gTnApDQogIHJFSFZfU3BhaW4gPC0gcmJpbmQockVIVl9TcGFpbiwgZGF0YS5mcmFtZShOcCA9IE5wLCBBY2N1cmFjeSA9IHJlc3VsdF9TcGFpbiRBY2N1cmFjeSwgU0UgPSByZXN1bHRfU3BhaW4kU0UpKQ0KfQ0KDQojIEFkZCBwb3B1bGF0aW9uIGxhYmVscw0KckVIVl9BdXN0cmFsaWEkUG9wdWxhdGlvbiA8LSAiQXVzdHJhbGlhIg0KckVIVl9TcGFpbiRQb3B1bGF0aW9uIDwtICJTcGFpbiINCnJFSFZfYm90aCA8LSByYmluZChyRUhWX0F1c3RyYWxpYSwgckVIVl9TcGFpbikNCg0KIyBQbG90IHdpdGggZXJyb3IgcmliYm9ucw0KZ2dwbG90KGRhdGEgPSByRUhWX2JvdGgsIGFlcyh4ID0gTnAsIHkgPSBBY2N1cmFjeSwgY29sb3IgPSBQb3B1bGF0aW9uKSkgKw0KICBnZW9tX2xpbmUoc2l6ZSA9IDAuMSkgKw0KICBnZW9tX3JpYmJvbihhZXMoeW1pbiA9IEFjY3VyYWN5IC0gMS45NiAqIFNFLCB5bWF4ID0gQWNjdXJhY3kgKyAxLjk2ICogU0UsIGZpbGwgPSBQb3B1bGF0aW9uKSwgYWxwaGEgPSAwLjIpICsNCiAgZ2VvbV92bGluZSh4aW50ZXJjZXB0ID0gNDAwLCAgbGluZXR5cGUgPSAiZGFzaGVkIikgKw0KICBnZW9tX3ZsaW5lKHhpbnRlcmNlcHQgPSA2MDAwLCBsaW5ldHlwZSA9ICJkYXNoZWQiKSArDQogIGdlb21faGxpbmUoeWludGVyY2VwdCA9IDAuNDUsIGxpbmV0eXBlID0gImRhc2hlZCIsIGNvbG9yID0gImJsdWUiKSArDQogIGdlb21faGxpbmUoeWludGVyY2VwdCA9IDAuMzUsIGxpbmV0eXBlID0gImRhc2hlZCIsIGNvbG9yID0gInJlZCIpICsNCiAgc2NhbGVfY29sb3JfbWFudWFsKHZhbHVlcyA9IGMoImJsdWUiLCAicmVkIikpICsNCiAgc2NhbGVfZmlsbF9tYW51YWwodmFsdWVzID0gYygiYmx1ZSIsICJyZWQiKSkgKw0KICBsYWJzKHggPSAiUmVmZXJlbmNlIHBvcHVsYXRpb24gc2l6ZSIsIHkgPSAiUHJlZGljdGlvbiBhY2N1cmFjeSIsDQogICAgICAgdGl0bGUgPSAiVGhlb3JldGljYWwgYWNjdXJhY3kgb2YgZXN0aW1hdGVkIGhvbG9iaW9udCB2YWx1ZSB3aXRoIGVycm9yIGJhcnMiKSArDQogIHNjYWxlX3lfY29udGludW91cygNCiAgICBicmVha3MgPSB1bmlxdWUoYygwLCAwLjEwLCAwLjIwLCAwLjM1LCAwLjQ1LCAwLjYwLCAwLjcwLCAwLjgwLCAwLjkwLCAxKSksDQogICAgbGltaXRzID0gYygwLCAxKQ0KICApICsNCiAgc2NhbGVfeF9jb250aW51b3VzKA0KICAgIGJyZWFrcyA9IHVuaXF1ZShjKDQwMCwgMjUwMCwgNTAwMCwgNjAwMCwgNzUwMCwgMTAwMDApKSwNCiAgICBsaW1pdHMgPSBjKDAsIDEwMDAwKSwNCiAgICBsYWJlbHMgPSBzY2FsZXM6OmNvbW1hDQogICkgKw0KICB0aGVtZV9taW5pbWFsKCkNCg0KDQpgYGANCg0K
