# Supplementary material for: Reliable enteric methane prediction from the cattle (Bos taurus) rumen microbiome
Source: Commun Biol. 2026 Apr 13;9:810. doi: 10.1038/s42003-026-10048-8 (PMC13265817; doi:10.1038/s42003-026-10048-8)
Supplement: Supplementary file 13 — Reporting Summary [file 42003_2026_10048_MOESM13_ESM.pdf]

nature portfolio

Corresponding author(s): Boris J. Sepulveda

Last updated by author(s): Mar 8, 2026

Reporting Summary

Nature Portfolio wishes to improve the reproducibility of the work that we publish. This form provides structure for consistency and transparency in reporting. For further information on Nature Portfolio policies, see our Editorial Policies and the Editorial Policy Checklist.

Statistics

For all statistical analyses, confirm that the following items are present in the figure legend, table legend, main text, or Methods section.

n/a	Confirmed	
☐	☒	The exact sample size (*n*) for each experimental group/condition, given as a discrete number and unit of measurement
☐	☒	A statement on whether measurements were taken from distinct samples or whether the same sample was measured repeatedly
☐	☒	The statistical test(s) used AND whether they are one- or two-sided<br>*Only common tests should be described solely by name; describe more complex techniques in the Methods section.*
☐	☒	A description of all covariates tested
☐	☒	A description of any assumptions or corrections, such as tests of normality and adjustment for multiple comparisons
☐	☒	A full description of the statistical parameters including central tendency (e.g. means) or other basic estimates (e.g. regression coefficient) AND variation (e.g. standard deviation) or associated estimates of uncertainty (e.g. confidence intervals)
☐	☒	For null hypothesis testing, the test statistic (e.g. *F*, *t*, *r*) with confidence intervals, effect sizes, degrees of freedom and *P* value noted<br>*Give P values as exact values whenever suitable.*
☐	☒	For Bayesian analysis, information on the choice of priors and Markov chain Monte Carlo settings
☒	☐	For hierarchical and complex designs, identification of the appropriate level for tests and full reporting of outcomes
☐	☒	Estimates of effect sizes (e.g. Cohen's *d*, Pearson's *r*), indicating how they were calculated

Our web collection on statistics for biologists contains articles on many of the points above.

Software and code

Policy information about availability of computer code

Data collection	no software was used
Data analysis	Basecalling was conducted using the software Guppy (Oxford Nanopore Technologies, Oxford, United Kingdom) with high accuracy mode (HAC) using versions 5.0.16 and 4.2.2.<br>Genotypes were imputed with FImpute (Sargolzaei, Chesnais, & Schenkel, 2014) and BEAGLE (Browning, Zhou, & Browning, 2018)<br>Sequencing quality control was evaluated with NanoPlot (De Coster & Rademakers, 2023) version 1.44.1.<br>The long reads were annotated with the script SQM_longreads.pl of SqueezeMeta pipeline (version 1.4) (Tamames & Puente-Sánchez, 2019) using the euk option to yield more eukaryotic annotations.<br>The CLR transformation was implemented with the unweighted option of the CLR function of the easyCODA R package version 0.40.2 (Greenacre, 2018)<br>The ILR transformation was implemented with the function ilr of the compositions R package (v 2.0-8) (van den Boogaart & Tolosana-Delgado, 2008)<br>CLR-transformed KO abundances were compared between Australia and Spain using the Wilcoxon rank-sum test implemented in the wilcox.test function of the R package stats version 4.4.3 (R Core Team, 2022), with the argument exact as false.<br>False-discovery rate (FDR) correction was applied across all KOs using the p.adjust function from the R package stats version 4.4.3 (R Core Team, 2022).<br>A genomic relationship matrix (GRM) was created with the Gmatrix function from the R package AGHmatrix version 2.1.4 (Amadeu et al., 2016)<br>scaling and centring the ruminal metagenome features (KOs in our case) across animals using the function scale of the R programming language version 4.4.3 (R Core Team, 2022).<br>GRM and MRM were inverted with the function solve of R version 4.4.3 (R Core Team, 2022).

To estimate the effects of the fixed effects on EME and the rumen metagenome features, we fitted linear models fitting EME and the KOs as the response variable linear models explained by the same fixed effects used in the prediction models using the functions asreml and wald.asreml of the R package ASReml-R (version 3) (Butler, Cullis, Gilmour, & Gogel, 2009).
The prediction models were conducted with the function asreml of the R package ASReml-R (version 3) (Butler et al., 2009).
The EME microbiability obtained in the Bayesian approach was obtained with BayesR3 (Breen et al., 2022) version released on May 28, 2025.
Enrichment analysis was performed using the enrichKEGG function of the clusterProfiler R package (v4.14.6) (Xu et al., 2024) with the organism parameter set to "ko" to query against the full KEGG Orthology database.
Bash and R scripts that were provided to the reviewers and will be available in GitHub upon manuscript acceptance.
References
Amadeu, R. R., Cellon, C., Olmstead, J. W., Garcia, A. A., Resende, M. F., & Munoz, P. R. (2016). AGHmatrix: R Package to Construct Relationship Matrices for Autotetraploid and Diploid Species: A Blueberry Example. The Plant Genome, 9(3), 1–10. doi:10.3835/plantgenome2016.01.0009
Breen, E. J., Macleod, I. M., Ho, P. N., Haile-Mariam, M., Pryce, J. E., Thomas, C. D., . . . Goddard, M. E. (2022). BayesR3 enables fast MCMC blocked processing for largescale multi-trait genomic prediction and QTN mapping analysis. Communications Biology, 5(1). doi:10.1038/s42003-022-03624-1
Browning, B. L., Zhou, Y., & Browning, S. R. (2018). A one-penny imputed genome from next-generation reference panels. The American Journal of Human Genetics, 103(3), 338–348.
Butler, D., Cullis, B. R., Gilmour, A., & Gogel, B. (2009). ASReml-R Reference Manual. Release 3.0.: Department of Primary Industries and Fisheries, Queensland Government, Australia. Retrieved from https://asreml.kb.vsni.co.uk/asreml-r-3-reference-manual/
De Coster, W., & Rademakers, R. (2023). NanoPack2: population-scale evaluation of long-read sequencing data. Bioinformatics, 39(5), btad311. doi:10.1093/bioinformatics/btad311
Greenacre, M. (2018). Compositional Data Analysis in Practice: Chapman & Hall / CRC Press.
R Core Team. (2022). R: A Language and Environment for Statistical Computing. Vienna, Austria: R Foundation for Statistical Computing. Retrieved from https://www.R-project.org/
Sargolzaei, M., Chesnais, J. P., & Schenkel, F. S. (2014). A new approach for efficient genotype imputation using information from relatives. BMC Genomics, 15(1), 478. doi:10.1186/1471-2164-15-478
Tamames, J., & Puente-Sánchez, F. (2019). SqueezeMeta, A Highly Portable, Fully Automatic Metagenomic Analysis Pipeline. Frontiers in Microbiology, 9(3349). doi:10.3389/fmicb.2018.03349
van den Boogaart, K. G., & Tolosana-Delgado, R. (2008). “compositions”: A unified R package to analyze compositional data. Computers & Geosciences, 34(4), 320–338. doi:https://doi.org/10.1016/j.cageo.2006.11.017
Xu, S., Hu, E., Cai, Y., Xie, Z., Luo, X., Zhan, L., . . . Yu, G. (2024). Using clusterProfiler to characterize multiomics data. Nature Protocols, 19(11), 3292–3320. doi:10.1038/s41596-024-01020-z

For manuscripts utilizing custom algorithms or software that are central to the research but not yet described in published literature, software must be made available to editors and reviewers. We strongly encourage code deposition in a community repository (e.g. GitHub). See the Nature Portfolio guidelines for submitting code & software for further information.

Data

Policy information about availability of data

All manuscripts must include a data availability statement. This statement should provide the following information, where applicable:

- Accession codes, unique identifiers, or web links for publicly available datasets
- A description of any restrictions on data availability
- For clinical datasets or third party data, please ensure that the statement adheres to our policy

The rumen metagenome sequence reads, and associated metadata of the Australian dataset are publicly available at the National Center for Biotechnology's Sequence Read Archive, Bioproject accession PRJNA1162230. Requests for the records of enteric methane emissions, metadata, host genotypes, and any other extra material of the Australian dairy cattle population should be addressed to Prof. Jennie E. Pryce, AgriBio, 5 Ring Rd, Bundoora VIC 3083, Australia; E-mail: jennie.pryce@agriculture.vic.gov.au. The rumen metagenome, methane measurements, and metadata of the Spanish population are available at the locations described by López-García, et al. 29. The genotypes of the Spanish dairy cattle should be addressed to Dr. Óscar González-Recio, CSIC, Dpt Mejora Genética Animal, Crta. de la Coruña km 7.5, 28040 Madrid, Spain; E-mail: (gonzalez.oscar@inia.csic.es).

Research involving human participants, their data, or biological material

Policy information about studies with human participants or human data. See also policy information about sex, gender (identity/presentation), and sexual orientation and race, ethnicity and racism.

Reporting on sex and gender	NA
Reporting on race, ethnicity, or other socially relevant groupings	NA
Population characteristics	NA
Recruitment	NA
Ethics oversight	NA

Note that full information on the approval of the study protocol must also be provided in the manuscript.

Field-specific reporting

Please select the one below that is the best fit for your research. If you are not sure, read the appropriate sections before making your selection.

☒ Life sciences ☐ Behavioural & social sciences ☐ Ecological, evolutionary & environmental sciences

For a reference copy of the document with all sections, see nature.com/documents/nr-reporting-summary-flat.pdf

Life sciences study design

All studies must disclose on these points even when the disclosure is negative.

Sample size	We analysed all animals we had with methane emissions (EME) and rumen fluid samples.<br>The Australian population included 403 Holstein lactating cows located at the Ellinbank SmartFarm (Ellinbank, Victoria, Australia). These cows were measured for intake and enteric EME in 11 cohorts between 2013 and 2017.<br>The Spanish population included 426 Holstein cows, either in their first or second lactation, from 14 commercial farms across four Northern Spanish regions (Cantabria, País Vasco, Navarra, and Gerona).<br>This sample size were sufficient for the prediction models to converge and estimate variance components.
Data exclusions	Animals without methane emissions records or without rumen metagenome were excluded.
Replication	The Australian were measured for intake using feed bins equipped with load cells and electronic monitoring linked to individual cow identification (Gallagher Animal Management Systems, Hamilton, New Zealand). Each cow's daily dry matter intake was recorded over the 32 days. Daily enteric methane production in the Australian population as grams per day (MePAustralia; g/d) was obtained with the sulphur hexafluoride (SF6) tracer method.<br>Ruminal fluid samples from Australian cows were collected via an oesophageal probe placed into the rumen via the mouth. An oro-ruminal sampling probe and a vacuum pump was used to collect samples. The oesophageal probe was a smooth-polished stainless-steel device comprising two parts: (1) a 170 mm long bolus of 42 mm diameter with a 2 mm screen and (2) a 4-meter-long flexible tube of 20 mm diameter. The collected samples were allowed to drain freely through a cheesecloth layer, separating rumen solids from the ruminal fluid, and then frozen at -80 °C.<br>In the Spanish cows, EME were measured using a non-dispersive infrared methane detector (The Guardian® NG) from Edinburgh Sensors (Livingston, Scotland, UK), also termed “sniffer”, installed in the feed bin of an automatic milking system. Individual methane concentration in Spain as parts per million (MeCSpain; ppm) was recorded for each cow during milking over a period of two to three weeks.<br>In Spain, a custom-built mechanical device was used to elevate the cow’s snout and approximately 100 ml of ruminal content from each cow was extracted by inserting a tube connected to a mechanical pump (Vacubrand ME 2SI, Wertheim, Germany) through the oesophagus. The obtained samples were then secured in sterilized containers. The hose and equipment were washed after each use. The samples were filtered using four layers of sterile cheesecloth to separate the solid elements, and the liquid portion was immediately frozen using liquid nitrogen vapours.<br>The frozen samples were conveyed to the lab in containers filled with liquid nitrogen and kept at -80 °C until analysis.
Randomization	This is not relevant for the study because we performed analysed on available data sets.
Blinding	This is not relevant for the study because we performed analysed on available data sets.

Reporting for specific materials, systems and methods

We require information from authors about some types of materials, experimental systems and methods used in many studies. Here, indicate whether each material, system or method listed is relevant to your study. If you are not sure if a list item applies to your research, read the appropriate section before selecting a response.

Materials & experimental systems

n/a	Involved in the study	
☒	☐	Antibodies
☒	☐	Eukaryotic cell lines
☒	☐	Palaeontology and archaeology
☐	☒	Animals and other organisms
☒	☐	Clinical data
☒	☐	Dual use research of concern
☒	☐	Plants

Methods

n/a	Involved in the study	
☒	☐	ChIP-seq
☒	☐	Flow cytometry
☒	☐	MRI-based neuroimaging

April 2023

Animals and other research organisms

Policy information about studies involving animals; ARRIVE guidelines recommended for reporting animal research, and Sex and Gender in Research

Laboratory animals	The study did not involve laboratory animals.
Wild animals	The study did not involve wild animals.
Reporting on sex	The study involved female cattle only (cows). However, the general tendency of the finding do not apply to only females as male cattle (bulls) share with cows the same variance components analysed (genomics and rumen metagenomics) and also produce enteric methane emissions.
Field-collected samples	For the Australian cows, over a 32-day period in an experimental facility, they had continuous access to feed, water, and a bare paddock (loafing area) for rest. The cows were outside except for twice-daily milking. Cows were fed with the diet described by Moate et al. (2021)<br>In Spain, cows' data were collected from 14 commercial farms across four Northern Spanish regions (Cantabria, País Vasco, Navarra, and Gerona).<br>References<br>Moate, P. J., Pryce, J. E., Marett, L. C., Garner, J. B., Deighton, M. H., Ribaux, B. E., . . . Williams, S. R. O. (2021). Measurement of Enteric Methane Emissions by the SF6 Technique Is Not Affected by Ambient Weather Conditions. Animals, 11(2), 528. doi:10.3390/ani11020528
Ethics oversight	The experiments in Australia included in this study were approved and undertaken in accordance with the Australian Code of Practice for the Care and Use of Animals for Scientific Purposes (NHMRC, 2013). Approval to proceed was granted by the Agricultural Research and Extension Animal Ethics Committee of the Department of Energy, Environment and Climate Action (application number 2013-14 was approved on August 22nd, 2013, and application number 2016-12 was approved on August 22nd, 2016). The experiments in Spain included in this study were conducted in accordance with Spanish Royal Decree 53/2013 for the protection of animals used for experimental and other scientific purposes and were approved by the Basque Institute for Agricultural Research and Development Ethics Committee (Neiker-OEBA-2017-004) on March 28, 2017.

Note that full information on the approval of the study protocol must also be provided in the manuscript.

Plants

Seed stocks	NA
Novel plant genotypes	NA
Authentication	NA
