# Supplementary material for: Reliable enteric methane prediction from the cattle (Bos taurus) rumen microbiome
Source: Commun Biol. 2026 Apr 13;9:810. doi: 10.1038/s42003-026-10048-8 (PMC13265817; doi:10.1038/s42003-026-10048-8)
Supplement: Supplementary file 1 — Transparent Peer Review file [file 42003_2026_10048_MOESM1_ESM.pdf]

nature portfolio

Peer Review File

Reliable enteric methane prediction from the cattle (Bos taurus) rumen microbiome

Corresponding Author: Dr Boris J Sepulveda

This file contains all reviewer reports in order by version, followed by all author rebuttals in order by version.

Version 0:

Reviewer comments:

Reviewer #1

(Remarks to the Author)

It is a very valuable dataset including animal microbiome with their host genome sequenced. The results are very interesting, they found animal microbiome explained much higher heritiability of enteric methane emissions than host genome. But, I think authors should add more analysis in MS to make the results convincible.

1. I suggest adding other statistical methods to predict enteric methane emissions (such as the Bayesian methods) and using animal microbiome (1,032 KOs) as predictor (similar to SNP).
2. Since the variance of host genomic explained much lower genetic variant of methane emissions than animal microbiome, this indicates that the variance of enteric methane emissions explained by host genome is small, while most of it is caused by environment and error. Thus, it can be predicted that the host genome explains small fraction of genetic variance for KOs, please use genomic SNPs to calculate the heritability of each animal microbiome in order to better explain why the use of animal microbiome improves predictive power.

3. Line 31, author says “By applying a new methodology…”, what kind of method has been develope，author should mention. Do you mean you use a rumen microbiome core to build an animal microbiome relationship matrix (MRM).
4. Figure 4. The prediction error bar should be added on the graph.

Reviewer #2

(Remarks to the Author)
Comments on “Reliable enteric methane predictor from the cattle rumen microbiome” by Sepulveda et al.

I congratulate the authors on developing variance component analysis that describes the contributions made by different important variables to EME productions. I like the approach, which is straight forward and yet important. I always wondered, for a given disease, what percent of the variation is really explained by the microbiome and what percent is explained by genetics and the external environment. Thus, their approach is broadly applicable to other contexts and the authors should expand the discussion section and highlight this aspect of the paper. I have a few comments regarding the methodology as follows. These are picky comments and should be addressable.

1. CLR transformation on page 12: Since the problem of interest is to perform variance component analysis, I was surprised why the authors did not consider ILR instead of CLR transformation. If there is no specific reason, then I suggest the authors to also consider ILR and see if this results in a different result. Hopefully the results are similar, otherwise how to interpret the differences.

2. Design matrix X on page 12: I am glad that the authors have considered design matrix without scaling or centring the CLRs. I agree with the authors regarding the effects of scaling the design matrix. However, the authors were concerned about near singularity of MRM matrices and added a small positive constant to the diagonal elements of the matrix. While this method (commonly known as ridging) is reasonable, why not perform singular value decomposition and Winsorize the smallest singular value. There is some recent literature on Winsorization based methods to work well. Anyway, this is very minor suggestion to consider. Alternatively, the authors can comment about it in the discussion section.

3. Confidence interval estimation: While the point estimator of the percent variation explained is important, it is equally important to derive asymptotic confidence intervals for this parameter. Under normality, one can derive the asymptotic confidence intervals, or perform Jackknife or bootstrap estimation. These are all available in standard packages.

4. Assumptions: A fundamental assumption made in this paper is that the outcome variable is normally distributed, and the errors are homoscedastic. In practice this may not be true. I am assuming that the authors have used restricted maximum likelihood estimators under normality assumption, which may not be robust to departures from normality and heteroscedasticity. Why not consider Rao's MINQUE or MINQE type estimators which are distribution free? There is a well-developed theory for such approaches. Of course, when the data are normally distributed then RMLE is optimal.

Reviewer #3

(Remarks to the Author)
The manuscript by Sepulveda et al provides a new tool to predict enteric methane emissions (EME) from ruminants. This new predictor is based on features of the ruminal microbiota, as well as on host genetics. As potential novelties distinguishing this predictor from others previously published, the authors in this work do not rely on taxonomic features in the rumen microbiome, but on the functional potential of the microbiome communities, as determined by the KEGG Orthologs (KOs) detected by long-read metagenomic sequencing in these samples. In addition, they opt for a novel strategy which does not scale the values of these microbiome features, arguing that this strategy may better highlight features with large contributions to the observed EMEs.
To develop this new predictor tool, they perform their analyses in two different cattle cohorts, from two countries: Spain and Australia. First, they identify the contribution to the total variance in EME from both the genetic variation, the microbial KOs and the combination of both (with or without interaction) using a linear mixed effects model. Then, the coefficients of the model are used to predict EME values, first using all the dataset and then using 10x 5-fold cross-validation analyses. Finally, they use their results to estimate the population size required to achieve a model predictive accuracy between 0.7 and 0.9, which can be used to guide future experiments.
While, as a reviewer, I see the potential interest of such a predictor and its innovative value, I have a few points to raise about this work, that I would like to see further clarified before recommending publication of this manuscript. These points are enumerated below
1-The model uses two independent cohorts. In these, samples were collected using different methods, EMEs were measured using different approaches (and, as far as I understand, these are not comparable), and these differences lead to two different models. However, the core microbiome functions are calculated for the entire population (i.e. both cohorts altogether). Since the differences in methodology may lead to potential batch effects and detection of KOs that are population-specific, it would be interesting to consider a specific core for each population, which may potentially lead to the inclusion of many more KOs. Do the model results improve when considering core KOs per population?
2-Related to the previous issue: considering only the KOs present in all animals of the study leads to a substantial reduction in the available information. The inclusion of KOs present in all animals is due to the usage of the CLR transformation. However, there are many strategies to circumvent the present of zeros in microbiome data. There are some studies, as mentioned in the manuscript, which add a pseudocount to zeros, but other alternatives involve zero imputation, or the use of transformation methods specific for sparse (microbiome) data, such as GMPR (Chen et al, PeerJ 2018; https://peerj.com/articles/4600/). It would be interesting to see if the use of any of these alternatives that allow dealing with zeros improves the current accuracy of the models. To avoid including too many zeros which can distort the analyses, a threshold on KO prevalence can be established in advance (i.e. 20%, 50% or similar prevalence to include a KO in the analysis).
3-Regarding the model interpretation, it would be interesting to discuss more the reasons underlying the differences in performance between the models using scaled and unscaled MRM matrices. This is interesting, since the differences in variance explained by scaled and unscaled matrices are quite large, but the accuracy of the predictions in the cross-validation scenario is very similar (Spain-HiBLUP: 0.17 for the unscaled MRM and 0.17 for the scaled MRM; Australia-HiBLUP: 0.41 for the unscaled MRM and 0.39 for the scaled MRM). Could this mean that there would be overfitting when using the unscaled matrices to estimate the variance explained?
4-Related to the previous question. The differences in one-time accuracy and the cross-validation approach are quite

striking, pointing to potential overfitting of the model? Could a feature selection approach (with the extended set of KOs for instance) before building the model help improve the performance without overfitting?
5-Related to the previous question. In general, the manuscript discusses the variance explained by the whole MRM matrix, but it would be very interesting to discern which KOs contribute the most to this variance explained. Are there any specific pathways of interest that could become targets for intervention?
6-The differences between the two populations are quite striking, with the one from Australia leading to much better prediction accuracies. This, as mentioned in the discussion, may be due to a higher variability within the Spanish population (14 farms vs 1 in Australia). However, a more detailed explanation would be appreciated. Is this higher variation the result of batch effects in the metagenomic sequencing of the different farms? Are there significant differences in the methane emissions across farms? Is the methane measurement less accurate with the technique used either in Spain or Australia? Additional (supplementary) plots showing the data variation across farms and populations would help clarifying the origin of the differences.

7-Figure 2 presents the results from Supplementary Table 3, showing the variance explained in the different models and populations, using the unscaled MRM. However, the numbers in the table do not match exactly the ones represented in the figure. For instance, Australia – HiBLUP. The microbiome explains 84% of the variance in EME according to the manuscript text and the data in Supplementary Table 3, but in the figure 2 it seems that the microbiome contribution is slower (around 81%). A similar issue is observed with the genetics contribution in the HBLUP model for Australia.

8-In Supplementary Table 2, the ho2 value corresponds to the sum of the the genetic heritability (Genetics column), microbiability (Microbiome column) and the interaction, for the HBLUP and HiBLUP models in both populations. However, in the Supplementary table 3, with the unscaled values, these numbers do not match. Aren’t they supposed to match?

9-In line 135, it is specified that the highest “one-time” accuracy value is 0.83, but in the corresponding table it is supposed to be 0.83. Also, it should be specified in the text that his is only in one of the two populations studied, the other being much lower.

10-In line 161, it is mentioned that the cross-validation accuracy in the HiBLUP model in Spain is 0.19, but it is 0.17 according to the Supplementary Tables 2 and 3. Here, again there are some discrepancies between the data shown in the supplementary tables and in Figure 3 and the numbers should be revised.

11-The calculation of the prediction accuracy is also misleading in the results. With the Australian ho2 values reported in Supplementary table 3 (0.86 in the HiBLUP model) and following Equation 1, the rEHV is of 0.5, not 0.45 (as reported in Figure 4). The value of ~0.45 corresponds to the calculation with the Spanish ho2 values. So even though the difference between the predicted and the observed accuracy is still small, it is larger than the one reported. This concerns results reported in lines 158-160 and 237-238.

12-In the Supplementary Data 1, the KO abundance tables are shown. However, these are supposed to represent absolute KO abundances. If so, why some KOs have non-integer values? I.e. K08483.

13-In lines 434-438, I believe two different notations are used for the vector of random additive genetic effects, g and also u (and their corresponding distributions and their interactions), please correct or indicate what is the difference. In Supplementary table 4 the “u” notation is used.

14-Lastly, even though the level of detail of the methods is remarkable, it would be beneficial to have access to the R code used to further evaluate the methodologies and results reported, especially when some of the numbers do not match with the figures, to assess the reason.

Version 1:

Reviewer comments:

Reviewer #1

(Remarks to the Author)
Authors have been well responsed all my questions, I have no other comments.

Reviewer #2

(Remarks to the Author)
The authors have addressed all my comments to my satisfaction. I am glad they considered ILR transformation and compared the results with CLR. Thus, a substory of this paper is the choice Aitchison's transformation to use. Perhaps this should be highlighted in the abstract as well. Also, many of the confidence intervals described in the paragraph after line 171 suggest that the some variance components are zero. Consequently, it does not make sense to say one "zero" reduced to another "zero". Perhaps they can highlight the consequence zero heritability coefficients in these cases. Otherwise, I have no further comments.

Reviewer #3

(Remarks to the Author)
After a first round of review, the authors have made an effort to address the issues raised and have improved the quality of the manuscript. Noteworthy, I personally appreciate the inclusion of the code to reproduce their analyses as part of the supplementary files.

I have only a couple more questions regarding the new version of the manuscript, that perhaps could help improve clarity in some sections:

1. I understand that the new BayesR model only uses the MRM for its estimation of the variance explained by KOs and microbiability, as opposed to the BLUP models, where different models use the MRM and/or the GRM. Is that correct? Is this also true for the accuracy predictions? One concern that I have is that the accuracy of predictions by the BayesR3 model is higher than the variance explained by the same model in both cohorts (for instance, the accuracy of prediction is of 0.62 in the Australian cohort while the microbiability is 0.34). How is this possible?
2. Some of the acronyms used (DIM, DMI, MeCSpain, MePAustralia, BLUP) shall be defined the first time they appear in the manuscript, as it can be confusing for the reader to look for the definitions elsewhere in the manuscript

Version 2:

Reviewer comments:

Reviewer #3

(Remarks to the Author)
Authors have addressed all my comments and I have no further requests concerning this manuscript

Open Access This Peer Review File is licensed under a Creative Commons Attribution 4.0 International License, which permits use, sharing, adaptation, distribution and reproduction in any medium or format, as long as you give appropriate credit to the original author(s) and the source, provide a link to the Creative Commons license, and indicate if changes were made.
In cases where reviewers are anonymous, credit should be given to 'Anonymous Referee' and the source.
The images or other third party material in this Peer Review File are included in the article’s Creative Commons license, unless indicated otherwise in a credit line to the material. If material is not included in the article’s Creative Commons license and your intended use is not permitted by statutory regulation or exceeds the permitted use, you will need to obtain permission directly from the copyright holder.
To view a copy of this license, visit https://creativecommons.org/licenses/by/4.0/

Reviewer 1

It is a very valuable dataset including animal microbiome with their host genome sequenced. The results are very interesting, they found animal microbiome explained much higher heritiability of enteric methane emissions than host genome. But, I think authors should add more analysis in MS to make the results convincible.

1. I suggest adding other statistical methods to predict enteric methane emissions (such as the Bayesian methods) and using animal microbiome (1,032 KOs) as predictor (similar to SNP).

AU: Thank you for this important suggestion. We have included a Bayesian analysis using BayesR3 in the manuscript now that predicts methane emissions from the KOs which directly treats explanatory variables as random effects. We found agreement in the partitioning of variances in EME between BayesR and BLUP. The variance explained by the KOs (microbiability) ranged from 0.27 to 0.31 in Australia and from 0.08 to 0.10 in Spain using BLUP, and was 0.34 in Australia and 0.15 in Spain using BayesR. Please see the paragraph "In Australia, the EME heritability…" .

2. Since the variance of host genomic explained much lower genetic variant of methane emissions than animal microbiome, this indicates that the variance of enteric methane emissions explained by host genome is small, while most of it is caused by environment and error. Thus, it can be predicted that the host genome explains small fraction of genetic variance for KOs, please use genomic SNPs to calculate the heritability of each animal microbiome in order to better explain why the use of animal microbiome improves predictive power.

AU: Agreeing with your point, the host genome explains a relatively small fraction of genetic variance for most KOs. We included the heritability of the KOs and more than 50% of them have a heritability close to zero, despite the heritability of some of them is higher than 0.5 (**Supplementary Figure 3**).

3. Line 31, author says "By applying a new methodology...", what kind of method has been develope, author should mention. Do you mean you use a rumen microbiome core to build an animal microbiome relationship matrix (MRM).

AU: Thank you for the message. We clarified the contribution in the abstract. Please see 'Here, we perform comprehensive analyses on host genetics, rumen microbiome, ….

4. Figure 4. The prediction error bar should be added on the graph.
Done. The methods were also updated with how the standard error was obtained

Reviewer 2

I congratulate the authors on developing variance component analysis that describes the contributions made by different important variables to EME productions. I like the approach, which is straight forward and yet important. I always wondered, for a given disease, what percent of the variation is really explained by the microbiome and what percent is explained by genetics and the external environment. Thus, their approach is broadly applicable to other contexts and the authors should expand the discussion section and highlight this aspect of the paper. I have a few comments regarding the methodology as follows. These are picky comments and should be addressable.

1. **CLR transformation on page 12:** Since the problem of interest is to perform variance component analysis, I was surprised why the authors did not consider ILR instead of CLR transformation. If there is no specific reason, then I suggest the authors to also consider ILR and see if this results in a different result. Hopefully the results are similar, otherwise how to interpret the differences.

AU: Thank you for the suggestion. As recommended, we tested the ILR transformation in addition to CLR. The results obtained with ILR were better than with CLR. Please see “Using ILR rather than CLR to construct the MRM led to higher microbiability estimates with up to 11% higher prediction accuracy…”. Also, please see the last sheet of **Supplementary Table 3** . Thank you very much for the suggestion.

2. **Design matrix X on page 12:** I am glad that the authors have considered design matrix without scaling or centring the CLRs. I agree with the authors regarding the effects of scaling the design matrix. However, the authors were concerned about near singularity of MRM matrices and added a small positive constant to the diagonal elements of the matrix. While this method (commonly known as ridging) is reasonable, why not perform singular value decomposition and Winsorize the smallest singular value. There is some recent literature on Winsorization based methods to work well. Anyway, this is very minor suggestion to consider. Alternatively, the authors can comment about it in the discussion section.

AU: Thank you for the recommendation. We included it in the discussion section. Please see: “To address near-singularity in the relationship matrices, we applied ridge regularization by adding a small constant to the diagonal elements. Alternative methods such as applying singular value decomposition (SVD) 32 followed by Winsorizing the smallest singular value are encouraged to be tested. Using the same metagenomic dataset, we found that the first two principal components of KO abundance within each population were correlated with EME 27, suggesting that the dominant axes of variation in the KO matrix are biologically informative. This supports the idea that SVD-based dimensionality reduction can retain relevant biological signal, and such approaches may warrant further exploration of EME

variance explained by the KOs. Additionally, future approaches that retain more microbial KOs and combine them with other taxonomic and functional metagenomic features may better explain EME variance. Finally, validation of our findings across larger datasets that span diverse environmental and management conditions is essential to assess the generalizability of our results."

3. **Confidence interval estimation:** While the point estimator of the percent variation explained is important, it is equally important to derive asymptotic confidence intervals for this parameter. Under normality, one can derive the asymptotic confidence intervals, or perform Jackknife or bootstrap estimation. These are all available in standard packages.

AU: Thank you for the suggestion. We have now included 95% confidence intervals to assess the uncertainty around the estimates of percent variance explained. This has been added to the Materials and Methods section (see: "We calculated 95% confidence intervals using the standard normal approximation. This approach relies on the estimate and its standard error…") and in the results (see **Supplementary Table 3**).

4. Assumptions: A fundamental assumption made in this paper is that the outcome variable is normally distributed, and the errors are homoscedastic. In practice this may not be true. I am assuming that the authors have used restricted maximum likelihood estimators under normality assumption, which may not be robust to departures from normality and heteroscedasticity. Why not consider Rao's MINQUE or MINQE type estimators which are distribution free? There is a well-developed theory for such approaches. Of course, when the data are normally distributed then RMLE is optimal.

AU:

We appreciate the reviewer's insightful comment. EME in Australia was approximately normally distributed (Shapiro–Wilk P-value = 0.11; Kolmogorov–Smirnov P-value = 0.65; **Supplementary Image 1**). EME in Spain showed deviations from normality with Shapiro–Wilk ($p = 5.7 \times 10^{-7}$), but the Kolmogorov–Smirnov test did not reject normality ($p = 0.16$). These results support that our outcome data are approximately normal, so the results are less likely to be biased by the choice of methods. Nevertheless, to complement REML, we also fitted BayesR, a Bayesian mixture method that has more flexibility than linear mixed model and the overall trend of the results from BayesR is similar to the results from REML. We have added this into the discussion along with a sentence to acknowledge the potential of distribution-free methods such as Rao's MINQUE as a future direction for variance component estimation of EME.

Reviewer 3

The manuscript by Sepulveda et al provides a new tool to predict enteric methane emissions (EME) from ruminants. This new predictor is based on features of the ruminal microbiota, as well as on host genetics. As potential novelties distinguishing this predictor from others previously published, the authors in this work do not rely on taxonomic features in the rumen microbiome, but on the functional potential of the microbiome communities, as determined by the KEGG Orthologs (KOs) detected by long-read metagenomic sequencing in these samples. In addition, they opt for a novel strategy which does not scale the values of these microbiome features, arguing that this strategy may better highlight features with large contributions to the observed EMEs.
To develop this new predictor tool, they perform their analyses in two different cattle cohorts, from two countries: Spain and Australia. First, they identify the contribution to the total variance in EME from both the genetic variation, the microbial KOs and the combination of both (with or without interaction) using a linear mixed effects model. Then, the coefficients of the model are used to predict EME values, first using all the dataset and then using 10x 5-fold cross-validation analyses. Finally, they use their results to estimate the population size required to achieve a model predictive accuracy between 0.7 and 0.9, which can be used to guide future experiments.

While, as a reviewer, I see the potential interest of such a predictor and its innovative value, I have a few points to raise about this work, that I would like to see further clarified before recommending publication of this manuscript. These points are enumerated below

1. The model uses two independent cohorts. In these, samples were collected using different methods, EMEs were measured using different approaches (and, as far as I understand, these are not comparable), and these differences lead to two different models. However, the core microbiome functions are calculated for the entire population (i.e. both cohorts altogether). Since the differences in methodology may lead to potential batch effects and detection of KOs that are population-specific, it would be interesting to consider a specific core for each population, which may potentially lead to the inclusion of many more KOs. Do the model results improve when considering core KOs per population?

   AU: Thank you for the comment. We understood your suggestion as referring to core microbiomes specific to the Australian and Spanish populations. The 1,032 KOs we used are indeed the core sets identified separately for each population, defined by 100% prevalence and after removing any KOs that could originate from the host.

2. Related to the previous issue: considering only the KOs present in all animals of the study leads to a substantial reduction in the available information. The inclusion of KOs present in all animals is due to the usage of the CLR transformation. However, there are many strategies to circumvent the present of zeros in microbiome data. There are some studies, as mentioned in the manuscript, which add a pseudocount to zeros, but other alternatives involve zero imputation, or the use of transformation methods specific for sparse (microbiome) data, such as GMPR (Chen et al, PeerJ 2018; https://peerj.com/articles/4600/). It would be interesting to see if the use of any of these alternatives that allow dealing with zeros improves the current accuracy of the models. To avoid including too many zeros which can distort the analyses, a threshold on KO prevalence can be established in advance (i.e. 20%, 50% or similar prevalence to include a KO in the analysis).

   AU: We appreciate the reviewer's insightful comment. However, after filtering out KOs reported in the host (bovine), all remaining KOs had a prevalence of 100% in both populations, circumventing the need to use lower prevalence thresholds for cohort-specific cores. Respect the population-specific cores, please refer to the previous point.
   However, we appreciate the reviewer point, and therefore, we have also discussed the point of including more KOs in future analyses. Please see "Additionally, future approaches that retain more microbial KOs and combine them with other taxonomic and functional metagenomic features may better explain EME variance….".

3. Regarding the model interpretation, it would be interesting to discuss more the reasons underlying the differences in performance between the models using scaled and unscaled MRM matrices. This is interesting, since the differences in variance explained by scaled and unscaled matrices are quite large, but the accuracy of the predictions in the cross-validation scenario is very similar (Spain-HiBLUP: 0.17 for the unscaled MRM and 0.17 for the scaled MRM; Australia-HiBLUP: 0.41 for the unscaled MRM and 0.39 for the scaled MRM). Could this mean that there would be overfitting when using the unscaled matrices to estimate the variance explained?

   AU: Thank you for the comment. After several tests and further validation, we found the analysis using scaled data is more reliable and therefore, we have removed the results of the MRM obtained with KOs unscaled across animals. We have updated this in the revised manuscript.

4. Related to the previous question. The differences in one-time accuracy and the cross-validation approach are quite striking, pointing to potential overfitting of the model? Could a feature selection approach (with the extended set of KOs for instance) before building the model help improve the performance without overfitting?

   AU: Thank you for the comment. Considering your point, in the revised manuscript, we have removed results regarding the one-time accuracy.

5. Related to the previous question. In general, the manuscript discusses the variance explained by the whole MRM matrix, but it would be very interesting to discern which KOs contribute the most to this variance explained. Are there any specific pathways of interest that could become targets for intervention?

   AU: Thank you very much for this helpful comment. We respectfully note that partitioning the variance explained by individual KOs is beyond the scope of the present manuscript. However, we now report the KOs with the largest effects on EME from the MBLUP analysis. Please see: "Of the 100 KOs with the largest effects on EME in the MBLUP analysis, 48 were shared between Australia and Spain (**Supplementary Table 4**)."
   Additionally, an enrichment analysis is now included in the manuscript and KEGG pathways are proposed as targets for intervention with the aim of reducing these EME. Please see section "KEGG Enrichment Analysis" in results.

6. The differences between the two populations are quite striking, with the one from Australia leading to much better prediction accuracies. This, as mentioned in the discussion, may be due to a higher variability within the Spanish population (14 farms vs 1 in Australia). However, a more detailed explanation would be appreciated. Is this higher variation the result of batch effects in the metagenomic sequencing of the different farms? Are there significant differences in the methane emissions across farms? Is the methane measurement less accurate with the technique used either in Spain or Australia? Additional (supplementary) plots showing the data variation across farms and populations would help clarifying the origin of the differences.

   AU: Thank you for the comment. Three reasons could explain differences between countries. First, EME from Spain were not adjusted for feed intake and body weight, which are highly associated with EME. Second, possible variable number of sequencing reads, and discrepancies introduced by sample preparation and sequencing techniques. Third, a higher environmental effect in the Spanish dataset could have not been captured by the model,

where an average of 30 animals were analysed in each farm (426 animals in 14 farms), while all animals in Australia (>400) were from the same farm under the same productive system and similar feeding conditions. Supporting this third reason, the effect of the farm was significant in the abundance of most KOs (p-value < 0.05; **Supplementary Table 2**). We also tested the variation of EME across farms in and Australia showed less variation (**Supplementary Figure 2**). These results suggest that the methane emissions are affected by different farm factors in the two countries (see paragraph “Apart from the higher EME microbiability in Australia compared to Spain previously discussed”).

7. Figure 2 presents the results from Supplementary Table 3, showing the variance explained in the different models and populations, using the unscaled MRM. However, the numbers in the table do not match exactly the ones represented in the figure. For instance, Australia – HiBLUP. The microbiome explains 84% of the variance in EME according to the manuscript text and the data in Supplementary Table 3, but in the figure 2 it seems that the microbiome contribution is slower (around 81%). A similar issue is observed with the genetics contribution in the HBLUP model for Australia.

AU: Thank you for comments 7 to 10 regarding the mismatch between the numerical values presented in the main text and the figures. This discrepancy arose due to the inadvertent use of different data versions in the supplementary materials and the manuscript body. We have now corrected this issue, and the manuscript and supplementary materials are aligned and consistent.

8. In Supplementary Table 2, the ho2 value corresponds to the sum of the the genetic heritability (Genetics column), microbiability (Microbiome column) and the interaction, for the HBLUP and HiBLUP models in both populations. However, in the Supplementary table 3, with the unscaled values, these numbers do not match. Aren’t they supposed to match?

AU: Please see answer to the previous question.

9. In line 135, it is specified that the highest ″one-time″ accuracy value is 0.83, but in the corresponding table it is supposed to be 0.83. Also, it should be specified in the text that his is only in one of the two populations studied, the other being much lower.

AU: Please see answer to the previous question.

10. In line 161, it is mentioned that the cross-validation accuracy in the HiBLUP model in Spain is 0.19, but it is 0.17 according to the Supplementary Tables 2 and 3. Here, again there are some discrepancies between the data shown in the supplementary tables and in Figure 3 and the numbers should be revised.

AU: Please see answer to the previous question.

11. The calculation of the prediction accuracy is also misleading in the results. With the Australian ho2 values reported in Supplementary table 3 (0.86 in the HiBLUP model) and following Equation 1, the rEHV is of 0.5, not 0.45 (as reported in Figure 4). The value of ~0.45 corresponds to the calculation with the Spanish ho2 values. So even though the difference between the predicted and the observed accuracy is still small, it is larger than the one reported. This concerns results reported in lines 158-160 and 237-238.

AU: Thank you for the comment. The new Bayesian model offers improved accuracy, and the document has been updated accordingly.

12. In the Supplementary Data 1, the KO abundance tables are shown. However, these are supposed to represent absolute KO abundances. If so, why some KOs have non-integer values? I.e. K08483.

    AU: Thank you for the comment. The non-integer values are generated by SqueezeMeta (the program used to annotate the reads). In SqueezeMeta, KO abundance tables show non-integer values because ORF counts are fractionally split among multiple annotations when database references have multiple KEGG labels. Please see this issue in the program's GitHub where the developers explain it: https://github.com/jtamames/SqueezeMeta/issues/115 .

13. In lines 434-438, I believe two different notations are used for the vector of random additive genetic effects, g and also u (and their corresponding distributions and their interactions), please correct or indicate what is the difference. In Supplementary table 5 the "u" notation is used.

AU: Thank you for the comment. We corrected it and used now g in all cases to notate random additive genetic effects. .

14. Lastly, even though the level of detail of the methods is remarkable, it would be beneficial to have access to the R code used to further evaluate the methodologies and results reported, especially when some of the numbers do not match with the figures, to assess the reason.

    AU: Thank you very much for the interest. The code is now provided for the revision and will be available in GitHub upon manuscript acceptance. **Supplementary Code 1** has the code to generate descriptive figures, estimate variance components and prediction accuracy of EME with BLUP models, and estimation of reference population size. Please notice that due to

commercial restrictions, the input data for **Supplementary Code 1** has the animal and sample IDs masked, and the genotypes, EME, metadata and fixed effects simulated, and therefore the results are not the same as those reported in the manuscript. However, the metagenome data is real.

Supplementary Code 2 is the code to identify KOs that could be from Bos taurus.

Supplementary Code 3 is an example of the parameter file used as input in BayesR3.

Reviewer 1

Authors have been well responsed all my questions, I have no other comments.

AU: we thank you again for reviewing our manuscript.

Reviewer 2

The authors have addressed all my comments to my satisfaction. I am glad they considered ILR transformation and compared the results with CLR. Thus, a substory of this paper is the choice Aitchison's transformation to use. Perhaps this should be highlighted in the abstract as well. Also, many of the confidence intervals described in the paragraph after line 171 suggest that the some variance components are zero. Consequently, it does not make sense to say one "zero" reduced to another "zero". Perhaps they can highlight the consequence zero heritability coefficients in these cases. Otherwise, I have no further comments.

AU: We appreciate the important comments from the reviewer. In consequence, we have updated the abstract to include the comparison between the two Aitchison's transformation used (ILR and CLR). Please see: ‘The isometric log-ratio transformation of KOs may better capture relationships between host genetics and the rumen microbiome than the centered log-ratio transformation, and BayesR yielded slightly higher microbe-explained EME variance than BLUP..’

Additionally, we agree that when confidence intervals for variance components include zero, these effects cannot be considered statistically different from zero. To address this, we have revised the results section to report estimates without implying comparative reductions when the CI includes zero. Please see: ‘In Australia, EME heritability was estimated as 0.27 ± 0.13 (SE)…’. Additionally, have added a statement in the discussion acknowledging this uncertainty. Please see: ‘Although the confidence intervals for most variance components in Spain include zero (**Supplementary Table 3**), meaning that we cannot rule out…’

Reviewer 3

After a first round of review, the authors have made an effort to address the issues raised and have improved the quality of the manuscript. Noteworthy, I personally appreciate the inclusion of the code to reproduce their analyses as part of the supplementary files.

I have only a couple more questions regarding the new version of the manuscript,

that perhaps could help improve clarity in some sections:

1. I understand that the new BayesR model only uses the MRM for its estimation of the variance explained by KOs and microbiability, as opposed to the BLUP models, where different models use the MRM and/or the GRM. Is that correct? Is this also true for the accuracy predictions? One concern that I have is that the accuracy of predictions by the BayesR3 model is higher than the variance explained by the same model in both cohorts (for instance, the accuracy of prediction is of 0.62 in the Australian cohort while the microbiability is 0.34). How is this possible?

AU: We appreciate the reviewer's insightful comments. BayesR does not use the MRM for variance estimation or prediction accuracy. Instead, BayesR estimates microbiability directly from the KO abundances included as explanatory variables in the model. Similarly, prediction accuracy is calculated based on the phenotypes predicted using these KO abundances, not from an MRM constructed from them.

Regarding the difference between prediction accuracy and microbiability, we appreciate the reviewer's comment, which prompted us to double-check our analysis. After reviewing, we found that the previous accuracy reported for BayesR was calculated as the correlation between the estimated microbial value (EMV) and the predicted values ($\hat{y}$) obtained from microbiota and fixed effects in the validation set. This was not the best metric to reflect prediction accuracy, as both EMV and $\hat{y}$ have the model predictions from the microbiota, overestimating the accuracy.

We have now corrected this by replacing $\hat{y}$ with the residual methane emissions adjusted for fixed effects (y^*), as described in the manuscript ("The accuracy of the predictions obtained with BayesR were also estimated with a 5-fold cross validation within population as the Pearson correlation between the estimated EMV of the validation set and their EME corrected for fixed effects…") in the section 'Prediction Accuracy' of Methods.

After updating the analysis, the BayesR prediction accuracies are now consistent with those obtained using BLUP: approximately 0.40 in Australia and 0.20 in Spain.

Please note that these accuracies are higher than the microbiability estimates (Australia: $r \approx 0.40$ and $m^2 \approx 0.30$ for BLUP, 0.34 for BayesR; Spain: $r \approx 0.20$ and $m^2 \approx 0.10$ for BLUP, 0.17 for BayesR) because the accuracy is calculated using y^* rather than the raw phenotype (y). Additionally, microbiability estimates are higher than the determination coefficients (Australia: $R^2 \approx 0.40^2 \approx 0.16$; Spain: $R^2 \approx 0.20^2 \approx 0.04$). Of note that microbiability is more comparable to the estimate squared r (R^2) not r.

We have updated the manuscript accordingly in the following sections:

- **Abstract:**
  "We report that the rumen microbiome explains up to 34% of the EME variance and when combined with the host genome the variance explained is up to 59% with prediction accuracies of up to 0.40."

- **Results:**
    - **Figure 4**
    - "Similar prediction accuracies were obtained with BLUP and BayesR, being 0.39 ± 0.07 (SE) with ILR in Australia and 0.20 ± 0.04 with ILR in Spain (**Figure 4**). The prediction accuracy was similar with CLR using BayesR: 0.40 ± 0.13 in Australia and 0.17 ± 0.12 CLR in Spain. Additionally, the regression slope from adjusted EME on the estimated microbial values($b_{EME,EMV}$) was 0.77 ± 0.20 (SE) in Australia and 0.90 ± 0.18 (SE) in Spain using ILR indicating possible overfitting. The $b_{EME,EMV}$ was 0.84 ± 0.40 (SE) in Australia and 0.93 ± 0.75 (SE) in Spain using CLR, which also indicates a possible overfitting"
- **Discussion:**
    - "The EME prediction accuracy of the models that used the rumen microbiome as a predictor was approximately 0.40 in Australia and between 0.17 and 0.20 in Spain (**Figure 4**; **Supplementary Table 3**; BayesR results)."
    - "Although BayesR and BLUP produced similar mean prediction accuracies, BayesR exhibited slightly lower standard errors of these accuracies, indicating more stable performance across cross-validation folds (**Figure 4; Supplementary Table 3**). BayesR also explained more of the EME variance attributable to the rumen microbiome, with microbiability estimates of 0.34 in Australia and 0.17 in Spain, compared with approximately 0.30 in Australia and 0.10 in Spain using BLUP."
    - "To complement REML, we also fitted BayesR, a Bayesian mixture method with more flexibility than REML, and the overall trend of the results from BayesR was similar to the results from REML, in terms of microbiability, prediction accuracy and a possible model overfitting."
    - "The simulated accuracy estimated for a reference population of 400 animals ranged between approximately 0.35 and 0.45 (**Figure 5**). This range is consistent with the cross-validation prediction accuracy obtained from the real dataset in Australia (approximately 0.40; **Figure 4**) and is higher than that observed in Spain (approximately 0.20). Based on the simulations and the results from Australia, the prediction accuracy of EME could reach approximately 0.80 to 0.90 with a reference population of around 6,000 animals with both ruminal metagenome and host genotype data (**Figure 5**)."

2. Some of the acronyms used (DIM, DMI, MeCSpain, MePAustralia, BLUP) shall be

defined the first time they appear in the manuscript, as it can be confusing for the reader to look for the definitions elsewhere in the manuscript

AU: We appreciate the reviewer’s insightful comments. This was now fixed.
